# Monarubins A–C from the Marine Shellfish-Associated Fungus *Monascus ruber* BB5

**DOI:** 10.3390/md18020100

**Published:** 2020-02-03

**Authors:** Yan-Qin Ran, Wen-Jian Lan, Yi Qiu, Qi Guo, Gong-Kan Feng, Rong Deng, Xiao-Feng Zhu, Hou-Jin Li, Jun Dong

**Affiliations:** 1 School of Traditional Chinese Medicine, Guangdong Pharmaceutical University, Guangzhou 510006, China; ranyq03@163.com; 2 School of Pharmaceutical Sciences, Sun Yat-sen University, Guangzhou 510006, China; lanwj@mail.sysu.edu.cn; 3 School of Chemistry, Sun Yat-sen University, Guangzhou 510275, China; qiuyi0771@163.com (Y.Q.); guoqi1228@126.com (Q.G.); 4 State Key Laboratory of Oncology in South China, Collaborative Innovation Center for Cancer Medicine, Cancer Center, Sun Yat-sen University, Guangzhou 510060, China; fenggk@sysucc.org.cn (G.-K.F.); dengrong@sysucc.org.cn (R.D.); zhuxfeng@mail.sysu.edu.cn (X.-F.Z.)

**Keywords:** *Monascus ruber*, marine shellfish, marine fungus, monarubin, cytotoxicity

## Abstract

Three new compounds, monarubins A–C (**1**, **6** and **13**), together with ten known compounds, including four alkaloids (**2**–**5**), two isocoumarins (**7** and **8**) and four polyketides (**9**–**12**), were isolated from marine shellfish-associated fungus *Monascus ruber* BB5. The structures were determined on the basis of the 1D and 2D NMR, MS, UV and IR data. The absolute configurations of compounds **3**, **6** and **13** were determined by ECD calculations. The NMR data of compounds deoxyhydroxyaspergillic acid (**3**) and 2-hydroxy-6-(1-hydroxy-1-methylpropyl)-3-*sec*-buthylpyrazine (**4**) were first reported. All of the isolated compounds were evaluated for their cytotoxic activities against human nasopharyngeal carcinoma cell lines CNE1, CNE2, SUNE1 and HONE1 and hepatocellular carcinoma cell lines QGY7701 and HepG2. Monarubin B (**6**) displayed potent cytotoxicities against the cancer cell lines HepG2 and QGY7701 with IC_50_ values of 1.72 and 0.71 μΜ, respectively; lunatinin (**7**) showed moderate cytotoxic activities against the cancer cell lines HepG2, QGY7701 and SUNE1 with the IC_50_ values of 9.60, 7.12 and 28.12 μΜ, respectively.

## 1. Introduction

Since 1990, marine aquatic production in China has ranked first in the world, among them, edible marine shellfishes are exceeding 75% of the total output [1]. From an academic perspective, the current researches on marine shellfishes mainly focus on the cultivation, breeding methods, heavy metal accumulation and shellfish toxins [2,3,4,5]. Molluscan shellfish concentrate microorganisms from surrounding waters during the filter-feeding process. Consequently, filter feeders are recognized as reservoirs for various microorganisms [6]. However, up to now, the marine shellfish-associated fungi are virtually untouched and represent new promising sources of bioactive metabolites.

Recently, a fungal strain *Monascus ruber* (collection number BB5) was isolated from the inner tissue of marine shellfish *Meretrix meretrix* collected from Hailing island, Yangjiang, China. The genera *Monascus* mainly consists of four species: *M*. *ruber, M*. *purpureus, M*. *anka* and *M. pilosus* [7]. In Asia, *Monascus* fermented rice has been used as a traditional medicine for centuries and as a healthy food to improve the color and delicacy of meat, fish and soybean products [7]. *Monascus* species can produce chemodiverse secondary metabolites including monascus pigments, monacolins, azaphilones, furanoisophthalides, amino acids, γ-lactams, pyranoindole alkaloids, benzenoids, furans, fatty acids and other structural types [8,9,10,11,12]. Some metabolites display various biological activities, such as anticancer, antibacterial, anti-inflammatory, antioxidative, enzyme inhibitor, antihypertensive and reducing plasma cholesterol activities [13,14,15,16,17]. For example, monacolin K, a polyketide first isolated from the *Monascus ruber* in 1973, is considered as the most efficacious compound for lowering plasma cholesterol and has been approved as a clinical prescription named lovastatin [8,17]. According to the data from IMS Health, over 10 billion tablets of monacolin K were distributed and more than 100 million prescriptions were written worldwide between the years 1988 and 2003 [17]. Considering the fungal biodiversity and huge metabolic potential, it is still worthwhile to investigate the secondary metabolites of *Monascus* species and their bioactivities. 

**Figure 1:**
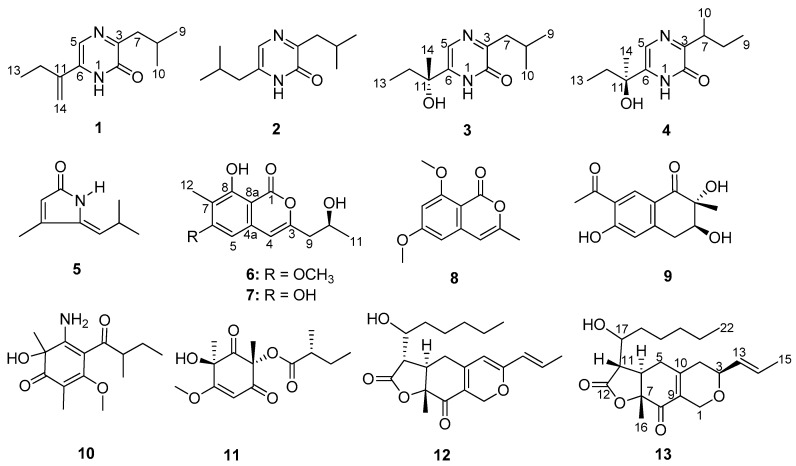
Chemical structures of compounds **1–13**.

In recent years, we have continuously conducted research on the metabolites of marine fungi associated with marine invertebrates such as soft corals, starfishes and sponges from the South China Sea. Our primary objective is to search for chemotherapeutic agents for prevalent malignancies with remarkably high incidence in South China, such as nasopharyngeal carcinoma and hepatocellular carcinoma. As a result, we obtained a series of cytotoxic metabolites, for example hirsutanol A [18], incarnal [19], chondrosterins A and J–M [20,21,22], pseudaboydin A [23], xanthocillin X dimethyl ether [24], 7-deacetylpyripyropene A [25], pyripyropene O [25], 13-dehydroxypyripyropene A [25] and 3,3′-cyclohexylidenebis(1*H*-indole) [26]. As part of our continuous project, culture of the fungus *Monascus ruber* BB5 with liquid GPY culture medium led to the isolation of three new compounds, monarubins A–C (**1**, **6** and **13**), together with ten known metabolites (**2**–**5** and **7**–**12**) (Figure 1). Here, we reported the isolation, the structure identification and cytotoxic activities of these compounds.

## 2. Results and Discussion

### 2.1. Structural Elucidation

**Figure 2:**
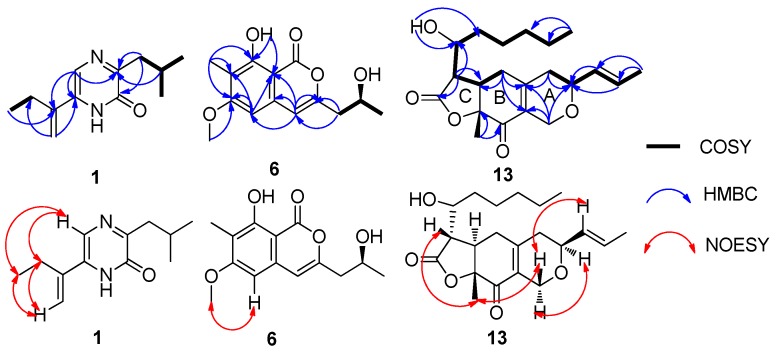
^1^H–^1^H COSY, key HMBC and key NOESY correlations of **1**, **6** and **13.**

Compound **1** was isolated as white needles. The molecular formula was determined as C_12_H_18_N_2_O by HR-(+) ESI-MS molecular ion peak at *m*/*z* 207.14923 [M + H]^+^ (calcd. for C_12_H_19_N_2_O, 207.14919) (Supplementary Figure S1), implying five degrees of unsaturation. The IR spectrum showed an amide group (3405 and 1639 cm^−1^). UV maxima at 201, 239 and 329 nm indicated the presence of a conjugated system. The ^13^C NMR and DEPT spectra showed three methyls, three methylenes, two methines and four quaternary carbons (Supplementary Figures S3 and S4). The ^1^H NMR and HMQC spectra displayed one amide proton [*δ*_H_ 10.83 (brs)], one aromatic proton [*δ*_H_ 7.41 (s), *δ*_C_ 120.9], one terminal olefinic double bond [*δ*_H_ 5.66 (s), 5.35 (s), *δ*_C_ 115.0], two methylenes [*δ*_H_ 2.69 (d, 7.0 Hz, 2H), *δ*_C_ 41.8; *δ*_H_ 2.46 (q, 7.0 Hz, 2H), *δ*_C_ 26.0] and three methyls [*δ*_H_ 1.17 (t, 7.0 Hz, 3H), *δ*_C_ 12.6; 0.98 (d, 7.0 Hz, 6H), *δ*_C_ 22.7] (Table 1 and Supplementary Figures S2 and S5). The correlations of H_2_-7/H-8/H_3_-9 and H_3_-10, and of H_2_-12/H_3_-13 in the ^1^H–^1^H COSY spectrum revealed the presence of isobutyl and −CH_2_CH_3_ fragments, respectively (Figure 2 and Supplementary Figure S6). The key HMBC correlations of H-5/C-6 (*δ*_C_ 135.1), C-11 (*δ*_C_ 140.8), and H_2_-12/C-6, C-11 and C-14 (*δ*_C_ 115.0), H_3_-13/C-11, H-14a (*δ*_H_ 5.35)/C-6, H-14b (*δ*_H_ 5.66)/C-6 and C-11 indicated the connection of the −CH_2_CH_3_ fragment with the C-11, which in turn was linked to C-6. The key HMBC correlations of H_2_-7/C-2 (*δ*_C_ 156.9), C-3 (*δ*_C_ 159.5), H-8/C-3 displayed the connection of the isobutyl to C-3 (Supplementary Figures S7 and S8). The NOESY correlations of H-5/H_2_-12, H-14a (*δ*_H_ 5.35)/H_2_-12, and H-14a/H_3_-13 suggested that the H-5, H_2_-12 and H_3_-13 were oriented on the same side of the molecule, H-14a being close to the ethyl and away from the pyrazinone ring (Figure 2). Therefore, the chemical structure of compound **1** was unambiguously established, and it was named monarubin A.

Compound **6** was obtained as a white solid. The HR-(+)ESI-MS spectrum displayed a strong quasi-molecular ion peak at *m*/*z* 265.10703 [M + H]^+^, corresponding to the molecular formula C_14_H_16_O_5_ (Supplementary Figure S19), requiring seven degrees of unsaturation. IR absorptions at 3380, 1678 and 1633 cm^−1^ indicated the existence of hydroxyl and ester carbonyl groups. The UV absorptions at λ_max_ 241, 280 and 331 nm displayed the conjugated system containing a benzene ring. The ^13^C NMR and DEPT spectra showed three methyls, one methylene, three methines and seven quaternary carbons (Supplementary Figures S21 and S22). The ^1^H NMR and HMQC spectra revealed one phenolic hydroxyl group [*δ*_H_ 11.15 (brs)], two double-bond protons [*δ*_H_ 6.32 (s), *δ*_C_ 97.2; *δ*_H_ 6.30 (s), *δ*_C_ 106.3], one oxygen-bearing methine multiplet [*δ*_H_ 4.26 (m), *δ*_C_ 65.5], one methoxy group [*δ*_H_ 3.90 (s), *δ*_C_ 55.8], one methylene group [*δ*_H_ 2.59 (dd, 14.4, 8.0 Hz), 2.66 (dd, 14.4, 4.4 Hz), *δ*_C_ 43.0], a methyl group on the benzene ring [*δ*_H_ 2.12 (s), *δ*_C_ 7.9] and the other methyl group [*δ*_H_ 1.30 (d, 6.0 Hz), *δ*_C_ 23.2] (Table 2 and Supplementary Figures S20 and S23). The presence of the –CH_2_CH(CH_3_)– moiety was based on the ^1^H–^1^H COSY correlations of H_3_-11/H-10/H_2_-9 (Supplementary Figure S24). The HMBC correlations from the phenolic hydroxyl at *δ*_H_ 11.15 (OH-8) to C-7 (*δ*_C_ 112.6), C-8 (*δ*_C_ 159.9) and C-8a (*δ*_C_ 99.9), from the methyl group at *δ*_H_ 2.12 to C-6 (*δ*_C_ 164.6), C-7 and C-8, from the methoxy group H_3_-13 (*δ*_H_ 3.90) to C-5 (*δ*_C_ 97.2) and C-6, from H-5 to C-4 (*δ*_C_ 106.3), C-6, C-7 and C-8a, from H-4 to C-3 (*δ*_C_ 153.8), C-5 and C-8a revealed the presence of 6-methyoxy-7-methy-8-hydroxy isochromone skeleton (Figure 2 and Supplementary Figure S25). Methoxy linked to C-6 was further supported by the NOESY correlations between proton H-5 and methyl protons H_3_-13 (Figure 2 and Supplementary Figure S26). The connection of C-3 with carbons C-9 (*δ*_C_ 43.0) and C-4 was established by HMBC correlations from the methylenic protons H_2_-9 (*δ*_H_ 2.59 and 2.66) to carbons C-3 and C-4, and from the olefinic proton H-4 (*δ*_H_ 6.30) to carbons C-3 and C-9. 

**Figure 3:**
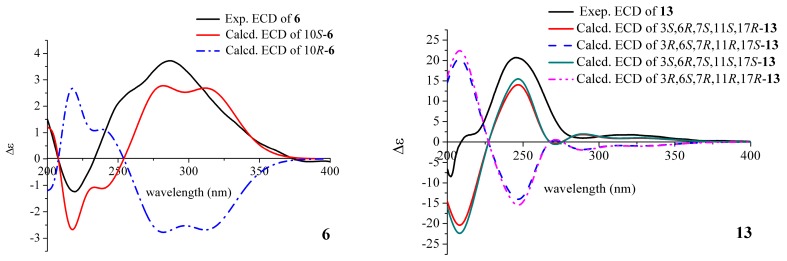
Comparison of the experimental and calculated ECD spectra of **6** and **13**.

The absolute configuration of **6** was elucidated by a comparison of the experimental and calculated electronic circular dichroism (ECD) curves (Figure 3). The calculated ECD curves were obtained by the time-dependent density functional theory (TD-DFT) at the B3LYP/6-311+G (d, p) level. The ECD curve of 10*S*-**6** was in line with the experimental curve. Based on the analysis above, the structure of compound **6** was assigned as (*S*)-8-hydroxy-3-(2-hydroxypropyl)-6-methoxy-7-methyl-1*H*-isochromen-1-one, and it was trivially named as monarubin B.

Compound **13** was isolated as a colorless oil. The molecular formula was established as C_21_H_30_O_5_ by HR-(+) ESI-MS at *m*/*z* 363.21664 [M + H]^+^ (calcd. for C_21_H_31_O_5_, 363.21664) (Supplementary Figure S39), which has seven degrees of unsaturation. The IR absorptions at 3422, 1781, 1695 and 1631 cm^−1^ and UV maxima at 243 nm indicated the presence of hydroxyl group, ketone carbonyl, ester carbonyl and a conjugated system. The ^13^C NMR, DEPT and HMQC spectra displayed three methyls, seven methylenes, six methines and five quaternary carbons. Among them, the quaternary carbons at *δ*_C_ 192.4 and 174.7 indicated the presence of one ketone carbonyl and one ester carbonyl, respectively and the four carbons at *δ*_C_ 82.9, 73.4, 69.4 and 63.6, their connection to oxygen atoms (Supplementary Figures S41–S43). The ^1^H NMR and HMQC spectra showed two trans olefinic protons [*δ*_H_ 5.78 (dqd, 15.6, 6.6, 1.2 Hz) and 5.52 (ddq, 15.6, 6.6, 1.2 Hz)], three methylenes [*δ*_H_ 4.44 (brd, 16.2 Hz), 4.37 (brd, 16.2 Hz), *δ*_C_ 63.6; 2.58 (m, 2H), *δ*_C_ 34.0; 2.35 (dd, 18.0, 9.0 Hz), 2.22 (ddd, 18.0, 3.0, 3.0 Hz), *δ*_C_ 36.2] and two oxygen-bearing methine multiplets [*δ*_H_ 4.22 (m), *δ*_C_ 69.4; 4.00 (m), *δ*_C_ 73.4] (Table 2 and Supplementary Figure S40). The ^1^H*−*^1^H COSY correlations of H_2_-4/H-3/H-13/H-14/H_3_-15 showed a partial structure –CH_2_CHCH=CHCH_3_ (Figure 2 and Supplementary Figure S44). The 1-hydroxyhexyl side chain was deduced from the ^1^H*−*^1^H COSY cross peaks of H_3_-22/H_2_-21/H_2_-20/H_2_-19/H_2_-18/H-17 and the HMBC correlations from H_3_-22 to C-21 (*δ*_C_ 22.5) and C-20 (*δ*_C_ 31.5), H-17 to C-18 (*δ*_C_ 35.0) and C-19 (*δ*_C_ 25.8) and OH-17 to C-18. The ^1^H*−*^1^H COSY cross-peaks of H-17/H-11/H-6 as well as the HMBC correlations from H-11 to C-17 (*δ*_C_ 69.4), C-5 (*δ*_C_ 34.0), C-6 (*δ*_C_ 41.3) and C-12 (*δ*_C_ 174.7) revealed that the 1-hydroxyhexyl side chain was connected with a γ-lactone (ring C). The HMBC correlations of H-1b to C-3 (*δ*_C_ 73.4), C-9 (*δ*_C_ 151.6) and C-10 (*δ*_C_ 129.6), H-3 to C-1 and C-10, H-4a to C-9 and C-10, H-4b to C-3, C-9 and C-10, H-5 to C-6 (*δ*_C_ 41.3), C-7 (*δ*_C_ 82.9), C-9 and C-10, H-6 to C-5 (*δ*_C_ 34.0), C-7 and C-8 (*δ*_C_ 192.4), H_3_-16 to C-7 and C-8 displayed the connection of ring A with ring B. Ring C was combined with ring B by the HMBC the correlations from H-6 to C-5, C-7, C-8 and C-11 (*δ*_C_ 174.7), H-11 to C-5, C-6 and C-12, H_3_-16 to C-7 and C-8 and was supported by the ^1^H*−*^1^H COSY correlations of H-11/H-6/H_2_-5 (Figure 2 and Supplementary Figures S44 and S45). Therefore, the planar structure of **13** was established and given the name monarubin C.

The relative stereochemistry of **13** was identified by the NOESY data. The large coupling constant (13.2 Hz) of the proton H-6 [*δ*_H_ 3.04 (ddd, 13.2, 9.6, 6.0 Hz)] with the proton H-11 [*δ*_H_ 2.75 (dd, 13.2, 3.0 Hz)] indicated that they were in opposite position on the ring. The NOESY cross peaks of H_3_-16 (*δ*_H_ 1.42) and H-11 (*δ*_H_ 2.75), H_3_-16 and H-1b (*δ*_H_ 4.44), H-1b and H-13 (*δ*_H_ 5.52) suggested that these protons were cofacial and assigned as *β*-orientation. The correlation between H-3 and H-1a (*δ*_H_ 4.37) suggested both of them having the *α*-orientation. The proton H-6 was determined as having an α-orientation because no NOESY correlation between H_3_-16 and H-6 was observed (Figure 2 and Supplementary Figure S46). Its absolute configuration was defined by calculated ECD. After calculation, it was found that the absolute configuration of C-17 was difficult to determine, 3*S*,6*R*,7*R*,11*S*,17*R*-**13** and 3*S*,6*R*,7*R*,11*S*,17*S*-**13** had almost the same result, which may be related to the fact that C-17 is too flexible in the branch, so only the absolute configuration of the remaining several chiral centers could be determined. By comparing the calculated curves with the experimental curve, the absolute configuration of compound **13** was defined as 3*S*,6*R*,7*R*,11*S* (Figure 3). 

**Figure 4:**
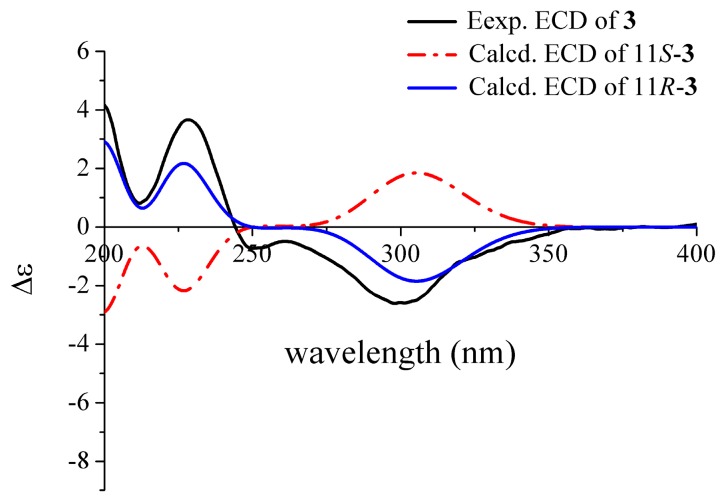
Comparison of the experimental and calculated ECD spectra of **3**.

Deoxyhydroxyaspergillic acid (**3**) was obtained as white needles. The molecular formula was established as C_12_H_20_N_2_O_2_ by HR-(+) ESI-MS molecular ion peak at *m*/*z* 225.16019 [M + H]^+^ (calcd. for C_12_H_21_N_2_O_2_, 225.15975) (Supplementary Figure S11), implying four degrees of unsaturation. This is the first report of its NMR data (Table 1 and Supplementary Figures S12 and S13) and absolute configuration, although its structure was reported by James D. Dutcher in 1957 [27]. Its absolute configuration was defined as 11*R* by comparison of the experimental and ECD curves (Figure 4). 

**Figure 5:**
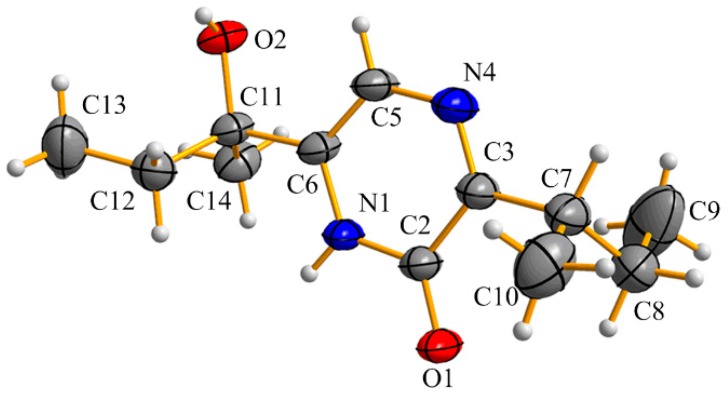
ORTEP diagram for the single-crystal X-ray structure of **4**.

2-Hydroxy-6-(1-hydroxy-1-methylpropyl)-3-*sec*-buthylpyrazine (**4**) was afforded as white needles. The molecular formula was determined as C_12_H_20_N_2_O_2_ by HR-(+) ESI-MS at *m*/*z* 225.15967 [M + H]^+^ (calcd. for C_12_H_21_N_2_O_2_, 225.15975) (Supplementary Figure S14), which has four degrees of unsaturation. Fortunately, we obtained a single crystal of **4** from a MeOH solution. The absolute configuration was unambiguously determined as 11*S* by single-crystal X-ray diffraction (Figure 5). Although its planar structure was reported by Sasaki et al. in 1968 [28], its NMR data (Table 1 and Supplementary Figures S15 and S16) and absolute configuration are reported for the first time.

Compounds **2**, **5** and **7–12** were elucidated as 3,6-diisobutyl-2(1*H*)-pyrazinone (**2**) [29], pulchellalactam (**5**) [30], lunatinin [also named 6, 8-dihydroxy-3-(2-hydroxypropyl)-7-methyl-1*H*-isochromen-1-one, **7**] [31], 6,8-dimethoxy-3-methylisocoumarin (**8**) [32], monaspurpurone (**9**) [33], 5-amino-2,6-dimethyl-6-hydroxy-4-(2′-methyl-1-oxobutyl)-3-methoxy-2,4-cyclohexadien-1-one (**10**) [34], phomaligol A (**11**) [35] and monascuspiloin (**12**) [36], respectively, by comparing their spectroscopic data (Supplementary Figures S9, S10, S17, S18 and S27–S38) with the literature values. Some of the known compounds also showed various bioactivities. For example, pulchellalactam (**5**) exhibited potent CD45 protein tyrosine phosphatase inhibitor activity [30], lunatinin (**7**) was found to have a significant antibiofilm activity [37], monaspurpurone (**9**) showed strong DPPH radical scavenging activity [17], and monascuspiloin (**12**) showed effective inhibitory activity against both androgen-dependent LNCaP and androgen-independent PC-3 human prostate cancer cells [38,39].

### 2.2. Biological Activity

Compounds **1–13** were evaluated for their cytotoxic activity against six cancer cell lines, including four human nasopharyngeal carcinoma cell lines CNE1, CNE2, SUNE1 and HONE1 and two human hepatocellular cancer cell lines HepG2 and QGY7701. As a result, compound **6** displayed potent cytotoxicities against the hepatocellular cancer cell lines HepG2 and QGY7701 with the IC_50_ values of 1.72 and 0.71 μΜ, respectively (Table 3 and Supplementary Tables S2 and S3). Compound **7** showed moderate inhibitory effects against the cancer cell lines HepG2, QGY7701 and SUNE1 with the IC_50_ values of 9.60, 7.12 and 28.12 μΜ, respectively (Table 3, Supplementary Tables S4–S6). Compound **8** exhibited weak cytotoxic activities against the cancer cell lines HepG2, QGY7701 and SUNE1 with the IC_50_ values of 46.10, 31.62 and 39.38 μΜ (Table 3). The new compounds monarubins A and C (**1** and **13**) and known compounds **2**–**5** and **9**–**12** were apparently inactive (IC_50_ values > 100 μΜ) in this assay. Hirsutanol A was used as a positive control [18,40,41].

## 3. Materials and Methods

### 3.1. General Procedures

Silica gel (SiO_2_, 200–300 mesh, Qingdao Puke parting Materials Co., Ltd. Qingdao, China) was used for the column chromatography. Preparative HPLC was performed using a Shimadzu LC-20AT HPLC pump (Shimadzu Corporation, Nakagyo*-*ku, Kyoto, Japan) equipped with a SPD-20A dual λ absorbance detector (Shimadzu Corporation, Nakagyo*-*ku, Kyoto, Japan) and a Capcell*-*Pak C18 UG80 HPLC column (250 *×* 20 mm, Shiseido Co., Ltd., Minato-ku, Tokyo, Japan). UV spectra were measured using Shimadzu UV–Vis–NIR spectrophotometer (Shimadzu Corporation, Nakagyo-ku, Kyoto, Japan). IR data were obtained on a PerkinElmer Frontier FT-IR spectrophotometer (PerkinElmer Inc., Waltham, MA, USA). CD spectra were measured on a J1700 circular dichroism spectrometer (Jasco, Kyoto, Japan). The high-resolution ESI-MS spectra were obtained on Thermo Fisher LTQ Orbitrap Elite High-Resolution liquid chromatography–mass spectrometer (Thermo Fisher Scientific Inc., Waltham, MA, USA). 1D and 2D NMR spectra were recorded on Bruker Avance Ⅲ 400, Avance 500 and Avance 600 spectrometers (Bruker Bio Spin AG, Industriestrasse 26, Fällanden, Switzerland). The chemical shifts corresponding to the residual solvent signals were the following (CDCl_3_: *δ*_H_ 7.260 and *δ*_C_ 77.000; acetone-*d*_6_: *δ*_H_ 2.050 and *δ*_C_ 29.840).

### 3.2. Fungal Strain and Culture Method

The marine fugus *Monascus ruber* BB5 was isolated from *Meretrix meretrix* collected from Hailing Island, Yangjiang, China. This fungal strain was conserved in 15% (*v*/*v*) glycerol aqueous solution at −80 °C. A voucher specimen (code name 2019FBB5) was deposited in the School of Chemistry, Sun Yat-sen University, Guangzhou, China. Analysis of the ITS rDNA by BLAST database screening provided 99.9% match with *Monascus ruber*.

The fermentation medium was glucose 20 g, peptone 10 g, yeast extract 2 g, Na_2_HPO_4_ 2 g, sea salt 25 g and water 1 L. Fungal mycelia were cut and transferred aseptically to 1000 mL conical flasks each containing 400 mL sterilized liquid medium. The flasks were static incubated at 28 °C for 30 d.

### 3.3. Extraction and Isolation

The fungal strain was fermented in total 200 L. The culture broth was filtered by cheesecloth and successively extracted three times with EtOAc. The extract was concentrated by low-temperature rotary evaporation to obtain a crude extract (46.7 g).

The extract was separated on silica gel column (diameter: 6 cm, length: 100 cm, silica gel: 200 g) with a gradient of petroleum ether–EtOAc (10:0–0:10, *v*/*v*) followed by EtOAc–MeOH (10:0–0:10) to give 15 fractions (Fr.1–Fr.15). Fr.10 was purified by silica gel column using a step gradient elution with petroleum ether–EtOAc (10:0–0:10) to get 12 subfractions (Fr.10-1–Fr.10-12). HPLC purification of Fr.10-2 with a solvent system CH_3_OH–H_2_O (70:30, *v*/*v*) gave compound **1** (RT = 55 min, 1 mg). Fr.7 was purified by C-18 reversed-phase column to get 9 subfractions (Fr.7-1–Fr.7-9). Fr.7-3 was eluted with CH_3_OH–H_2_O (60:40, *v*/*v*) by preparative HPLC to obtain compounds **2** (RT = 49 min, 11.2 mg) and **10** (RT = 44 min, 22.9 mg). Fr.11 was chromatographed by silica gel column using a step gradient elution with petroleum ether–EtOAc (10:0–0:10) to get 25 subfractions (Fr.11-1–Fr.11-25). Fr.11-11 was purified with a solvent system CH_3_OH–H_2_O (65:35, *v*/*v*) by preparation HPLC to obtain compounds **3** (RT = 57 min, 5.5 mg), **4** (RT = 61 min, 6 mg), **6** (RT = 132 min, 6.6 mg) and **8** (RT = 51 min, 5.3 mg). Compound **11** (RT = 57 min, 1 mg) was obtained from the Fr.11-13 by preparation HPLC using CH_3_OH–H_2_O (60:40, *v*/*v*) as elution. Fr.11-14 was purified by preparative HPLC (CH_3_OH–H_2_O, 70:30, *v*/*v*) to obtain compounds **7** (RT = 53 min, 1.6 mg) and **9** (RT = 35 min, 1 mg). Fr.8 was separated by silica gel column using a step gradient elution with petroleum ether EtOAc (10:0-10:0) to get 7 subfractions (Fr.8-1–Fr.8-7), and compound **5** (RT = 37 min, 23 mg) was obtained from Fr.8-2 by preparative HPLC using CH_3_OH–H_2_O (80:20, *v*/*v*) as eluent. Fr.8-4 was purified by preparative HPLC (CH_3_OH–H_2_O, 80:20, *v*/*v*) to obtain compounds **12** (RT = 47 min, 10 mg) and **13** (RT = 42 min, 1.5 mg).

Monarubin A (**1**). UV (MeOH) λ_max_ (logε) 330 (3.69), 239 (3.71), 202 (3.90). IR *υ*_max_ 3405, 2963, 1639, 1583, 1411, 1265, 1096, 1044, 801 cm^−1^. ^1^H and ^13^C NMR data, see Table 1; HR-(+) ESI-MS *m*/*z* 207.14923 [M + H]^+^ (calcd. for C_12_H_19_N_2_O, 207.14919).

Deoxyhydroxy aspergillic acid (**3**). ECD (0.4 mM, MeOH) λ_max_ (Δε) 211 (+0.51), 228 (+4.24), 309 (−2.63) nm. ^1^H and ^13^C NMR data, see Table 1; HR-(+) ESI-MS *m*/*z* 225.16019 [M + H]^+^ (calcd. for C_12_H_21_N_2_O_2_, 225.15975).

2-Hydroxy-6-(1-hydroxy-1-methylpropyl)-3-sec-buthylpyrazine (**4**). ^1^H and ^13^C NMR data, see Table 1; HR-(+) ESI-MS *m*/*z* 225.15967 [M + H]^+^ (calcd. for C_12_H_21_N_2_O_2_, 225.15975).

Monarubin B (**6**). [α]^25^_D_ + 19 (*c* = 0.001, MeOH), UV (MeOH) λ_max_ (logε) 331 (3.62), 280 (3.71), 241 (4.49). ECD (0.7 mM, MeOH) λ_max_ (Δε) 289 (+3.79) nm. IR *υ*_max_ 3380, 2927, 1673, 1634, 1566, 1520, 1423, 1348, 1252, 1124, 939, 796 cm^−1^. ^1^H and ^13^C NMR data, see Table 2; HR-(+) ESI-MS *m*/*z* 265.10703 [M + H]^+^ (calcd. for C_14_H_17_O_5_, 265.10705).

Monarubin C (**13**). [α]^25^_D_ + 43 (*c* = 0.001, MeOH), UV (MeOH) λ_max_ (logε) 243 (3.89). ECD (0.5 mM, MeOH) λ_max_ (Δε) 246 (20.99) nm. IR *υ*_max_ 3422, 2934, 1781, 1695, 1631, 1392, 1261, 1209, 1094, 1051, 965, 804 cm^−1^. ^1^H and ^13^C NMR data, see Table 3; HR-(+) ESI-MS *m*/*z* 363.21664 [M + H]^+^ (calcd. for C_21_H_31_O_5_, 363.21664). 

### 3.4. Computational Methods

The absolute configurations of compounds **3, 6** and **13** were determined by ECD calculations using Gaussian 09 software. Conformational searches were carried out by means of the Spartan’14 software using a Molecular Merck force field (MMFF). The conformers with a Boltzmann distribution over 2% were chosen for ECD calculations by TD-DFT method at the B3LYP/6-311+G (d, p) level in methanol (Supplementary Figures S47–S54). The ECD spectra were generated by the SpecDis 3.0 using a Gaussian band shape with a 0.3 eV exponential half-width from dipole-length dipolar and rotational strengths.

### 3.5. X-ray Crystallographic Analysis for **4**

Crystals of **4** were obtained from MeOH solution. X-ray diffraction data were collected on a Bruker SMART APEX CCD diffractometer with Cu *Kα* radiation (λ = 1.54184 Å). The structure was solved by direct methods using SHELXS-97, and refined through the program and full-matrix least-squares calculations. Hydrogen atoms were refined at calculated positions, and all non-hydrogen atoms were fixed anisotropically. Crystallographic data of **4** have been deposited at the Cambridge Crystallographic Data Centre (CCDC number: 1958991).

Crystal data of **4**: monoclinic, C_12_H_20_N_2_O_2_, *a* = 11.77370(10) Å, *b* = 6.91360(10) Å, *c* = 16.5776(2) Å, α = γ = 90°, β = 91.3700(10)°, *V* = 1349.01(3) Å^3^, space group P2_1_, *Z* = 2, *D*c = 1.104 g/cm^3^, μ = 0.607 mm^−1^, and *F*(000) = 488.0. Independent reflections: 5242 (*R*_int_ = 0.0525). The final *R*_1_ value was 0.0570, *wR*_2_ = 0.1619 (*I* > 2σ(I)). The goodness of fit on *F*^2^ was 1.039. Flack parameter = −0.04(7) (Supplementary Table S1).

### 3.6. Cytotoxicity Assay

Human nasopharyngeal carcinoma cell lines CNE1, CNE2, SUNE1 and HONE1 and hepatocellular carcinoma cell lines QGY-7701 and HepG2 were generously provided by Professor Mu-Sheng Zeng (Cancer Center, Sun Yat-sen University, Guangzhou, China) and conserved in the State Key Laboratory of Oncology in South China, Cancer Center, Sun Yat-sen University. The culture medium was DMEM (Gibco^®^, Suzhou, China) with 10% fetal bovine serum (FBS) (Gibco^®^). All of the cells were authenticated using short tandem repeat profiling, tested for *Mycoplasma* contamination, and cultured for less than 2 months.

The in vitro cytotoxic activities of **1**–**13** were determined by means of the colorimetric 3-(4,5-dimethylthiazol-2-yl)-2,5-diphenyl-2*H*-tetrazolium bromide (MTT) assays and the assays were performed in triplicate. The cells were maintained at 37 °C with 5% CO_2_ and were seeded in 96-well plates. The volume and number of cells seeded was 0.2 mL and 3 *×* 10^3^ per well. Compounds **1**–**13** were added to the cultures at different concentrations (1.5625–100 μM); moreover, compound **6** was tested at additional different concentrations (0.037–27 μM). Solvent DMSO was added as the control. After 72 h, 0.5 mg/mL MTT was added to the culture medium, and the plates were incubated for 4 h at 37 °C. The supernatant was removed. The formazan crystals were dissolved in DMSO (150 µL), the absorbance was measured at 570 nm by using a microplate reader (Bio-Rad, Hercules, CA, USA), and the data were analyzed with the CalcuSystem software package. Hirsutanol A, a potent anticancer agent isolated from marine fungal metabolites, was used as a positive control, and its cytotoxic activities against the tested cancer cell lines were shown in Table 3.

## 4. Conclusions

Three new metabolites, monarubins A–C, together with ten known compounds containing alkaloids, isocoumarins and polyketides were isolated from the fungal strain *Monascus ruber* BB5. Monarubin B (**6**) showed potent cytotoxic activities against the human hepatocellular cancer cell lines HepG2 and QGY7701 with IC_50_ values of 1.72 and 0.71 μΜ, and lunatinin (**7**) exhibited moderate inhibitory effects against the human hepatocellular cancer cell lines HepG2, QGY7701, and nasopharyngeal carcinoma cancer cell line SUNE1 with IC_50_ values of 9.60, 7.12 and 28.12 μΜ, respectively. Compound **8** exhibited weak cytotoxic activities against the cancer cell lines HepG2, QGY7701 and SUNE1 with IC_50_ values in the range of 30–50 μΜ. Compounds **6**–**8** have the same isocoumarin skeleton. By comparing their structures and activities, we can preliminarily conclude that 8-OH is an essential active functional group. Our finding once again demonstrates that *Monascus* are genius strains with the ability to produce chemodiverse bioactive compounds.

## Figures and Tables

**Table 1:** ^1^H and ^13^C NMR data for compounds **1, 3** and **4** in CDCl_3_ (*δ* in ppm).

Position	1 ^a^		3 ^b^		4 ^b^	
	δ_C_, type	δ_H_, mult. (*J*, Hz)	δ_C_, type	δ_H_, mult. (*J*, Hz)	δ_C_, type	δ_H_, mult. (*J*, Hz)
1-NH		10.83, brs		11.53, brs		11.50, brs
2	156.9, C		157.0, C		156.5, C	
3	159.5, C		157.6, C		161.7, C	
5	120.9, CH	7.41, s	120.0, CH	7.30, s	120.0, CH	7.32, s
6	135.1, C		142.5, C		142.0, C	
7	41.8, CH_2_	2.69, d (7.0)	41.5, CH_2_	2.68, dd (14.4, 7.6) 2.62, dd (14.4, 7.2)	36.6, CH	3.24, ddq (6.8, 6.8, 6.8)
8	26.9, CH	2.23, tqq (7.0, 7.0, 7.0)	26.9, CH	2.18, m	27.6, CH_2_	1.53, ddq (13.6, 6.8, 6.8) 1.79, ddq (13.6, 6.8, 6.8)
9	22.7, CH_3_	0.98, d (7.0)	22.6, CH_3_	0.95, d (6.6)	11.9, CH_3_	0.89, t (8.4)
10	22.7, CH_3_	0.98, d (7.0)	22.6, CH_3_	0.95, d (6.6)	17.6, CH_3_	1.21, d (6.8)
11	140.8, C		72.1, C		72.2, C	
12	26.0, CH_2_	2.46, q (7.0)	35.4, CH_2_	1.86, m	35.3, CH_2_	1.87, m
13	12.6, CH_3_	1.17, t (7.0)	8.1, CH_3_	0.90, t (7.2)	8.1, CH_3_	0.92, t (7.6)
14	115.0, CH_2_	a. 5.35, s b. 5.66, s	27.1, CH_3_	1.57, s	27.1, CH_3_	1.57, s
11-OH				3.77, brs		3.482, brs

^a 1^H (500 MHz) and ^13^C (125 MHz) NMR; ^b 1^H (400 MHz) and ^13^C (100 MHz) NMR.

**Table 2:** ^1^H and ^13^C NMR data for compounds **6** and **13** in CDCl_3_ (*δ* in ppm).

6 ^a^			13 ^b^		
Position	*δ*_C_, type	*δ*_H_, mult. (*J*, Hz)	Position	δ_C,_ type	δ_H_, mult. (*J*, Hz)
1	166.5, C		1	63.6, CH_2_	a. 4.37, brd (16.2) b. 4.44, brd (16.2)
3	153.8, C		3	73.4, CH	4.00, m
4	106.3, CH	6.30, s	4	36.2, CH_2_	a. 2.22, ddd (18.0, 3.0, 3.0) b. 2.35, dd (18.0, 9.0)
4a	136.5, C		5	34.0, CH_2_	2.58, m
5	97.2, CH	6.32, s	6	41.3, CH	3.04, ddd (13.2, 9.6, 6.0)
6	164.6, C		7	82.9, C	
7	112.6, C		8	192.4, C	
8	159.9, C		9	151.6, C	
8a	99.9, C		10	129.6, C	
9	43.0, CH_2_	2.59, dd (14.4, 8.0) 2.66, dd (14.4, 4.4)	11	48.8, CH	2.75, dd (13.2, 3.0)
10	65.5, CH	4.26, m	12	174.7, C	
11	23.2, CH_3_	1.30, d (6.0)	13	130.0, CH	5.52, ddq (15.6, 6.6, 1.2)
12	7.9, CH_3_	2.12, s	14	129.0, CH	5.78, dqd (15.6, 6.6, 1.2)
13	55.8, CH_3_	3.90, s	15	17.8, CH_3_	1.73, ddd (6.6, 1.2, 0.6)
8-OH		11.15, brs	16	16.6, CH_3_	1.42, s
10-OH		1.71, brs	17	69.4, CH	4.22, m
			18	35.0, CH_2_	1.55, m
			19	25.8, CH_2_	1.53, m 1.33, m
			20	31.5, CH_2_	1.33, m
			21	22.5, CH_2_	1.33, m
			22	14.0, CH_3_	0.91, t (7.2)
			17-OH		2.07, d (4.8)

^a 1^H (400 MHz) and ^13^C (100 MHz) NMR; ^b 1^H (600 MHz) and ^13^C (150 MHz) NMR.

**Table 3:** Cytotoxic activities of compounds **1–13** (IC_50_ ± SD, μM, *n* = 3).

Compounds	Human Nasopharyngeal Carcinoma Cell Lines				Human Hepatocellular Cancer Cell Lines	
	CNE1	CNE2	HONE1	SUNE1	HepG2	QGY7701
**1**	− ^a^	−	−	90.55 ± 1.58	−	−
**2**	−	−	−	92.53 ± 1.10	−	−
**3**	81.91 ±1.81	−	−	−	−	−
**4**	63.88 ± 1.22	−	−	92.78 ± 1.73	−	−
**5**	−	91.78 ± 1.90	−	64.35 ± 0.89	−	−
**6**	−	75.70 ± 1.09	−	72.07 ± 0.65	1.72 ± 0.35	0.71 ± 0.12
**7**	−	85.66 ± 1.69	−	28.12 ± 0.75	9.60 ± 0.46	7.12 ± 0.36
**8**	−	−	−	39.38 ± 0.58	46.10 ± 0.91	31.62 ± 1.23
**9**	70.96 ± 1.51	−	−	−	−	−
**10**	72.72 ± 1.36	−	−	−	−	−
**11**	92.87 ± 2.10	−	−	−	−	−
**12**	−	−	−	−	−	−
**13**	50.55 ± 0.88	−	−	−	−	−
Hirsutanol A	10.08 ± 0.92	12.72 ± 0.86	17.40 ± 0.52	3.50 ± 0.28	10.11 ± 0.69	21.12 ± 1.01

^a^ In the table, “−” means IC_50_ value > 100 μΜ.

## Supplementary Materials

The following are available online at https://www.mdpi.com/1660-3397/18/2/100/s1. Figures S1–S45: The HR-(+)ESI-MS and NMR spectra of compounds **1****–13**, Figures S46–S53: the most stable conformers of **3**, **6** and **13**, Table S1 and CIF file: the X-ray crystallography data of **4** and Tables S2–S6: the cytotoxic activities data of **6** and **7**.
