# Supplementary material for: Monarubins A–C from the Marine Shellfish-Associated Fungus Monascus ruber BB5
Source: Mar Drugs. 2020 Feb 3;18(2):100. doi: 10.3390/md18020100 (PMC7073648; doi:10.3390/md18020100)
Supplement: Supplementary file 1 [file marinedrugs-18-00100-s001.pdf]

Supplementary Material

Monarubins A–C from the Marine Shellfish-Associated Fungus *Monascus ruber* BB5

Yan-Qin Ran [1], Wen-Jian Lan [2], Yi Qiu [3], Qi Guo [3], Gong-Kan Feng [4], Rong Deng [4], Xiao-Feng Zhu [4], Hou-Jin Li [3,*], and Jun Dong [1,*]

[1] School of Traditional Chinese Medicine, Guangdong Pharmaceutical University, Guangzhou 510006, China; ranyq03@163.com (Y.-Q.R.)
[2] School of Pharmaceutical Sciences, Sun Yat-sen University, Guangzhou 510006, China; lanwj@mail.sysu.edu.cn (W.-J.L.)
[3] School of Chemistry, Sun Yat-sen University, Guangzhou 510275, China; qiuyi0771@163.com (Y.Q.); guoqi1228@126.com (Q.G)
[4] State Key Laboratory of Oncology in South China, Collaborative Innovation Center for Cancer Medicine, Cancer Center, Sun Yat-sen University, Guangzhou 510060, China; fenggk@sysucc.org.cn (G.-K.F.); dengrong@sysucc.org.cn (R.D.); zhuxfeng@mail.sysu.edu.cn (X.-F.Z.)
* Correspondence: ceslhj@mail.sysu.edu.cn (H.-J.L.); Dongjun@gdpu.edu.cn (J.D.); Tel: +86-20-84113698 (H.-J.L.); 86-13822726106 (J.D.)

List of contents:

Figure S1. HR-(+) ESI-MS spectrum of monarubin A (**1**). 6
Figure S2. ^{1}H NMR spectrum of monarubin A (**1**) in $CDCl_3$ (500 MHz). 7
Figure S3. ^{13}C NMR spectrum of monarubin A (**1**) in $CDCl_3$ (125 MHz).......... 8
Figure S4. DPET 135 spectrum of monarubin A (**1**) in $CDCl_3$ (125 MHz).......... 9
Figure S5. HMQC spectrum of monarubin A (**1**) in $CDCl_3$. 10
Figure S6. ^{1}H–^{1}HCOSY spectrum of monarubin A (**1**) in $CDCl_3$. 11
Figure S7. HMBC spectrum of monarubin A (**1**) in $CDCl_3$.......... 12
Figure S8. NOEY spectrum of monarubin A (**1**) in $CDCl_3$. 13

Figure S9. ^{1}H NMR spectrum of 3,6-diisobutyl-2(1*H*)-pyrazinone (**2**) in $CDCl_3$ (400 MHz). 14

Figure S10. ^{13}C NMR spectrum of 3,6-diisobutyl-2(1*H*)-pyrazinone (**2**) in $CDCl_3$ (100 MHz). 15

Figure S11. HR-(+) ESI-MS spectrum of deoxyhydroxyaspergillic acid (**3**). 16

Figure S12. ^{1}H NMR spectrum of deoxyhydroxyaspergillic acid (**3**) in $CDCl_3$ (400 MHz). 17

Figure S13. ^{13}C NMR spectrum of deoxyhydroxyaspergillic acid (**3**) in $CDCl_3$ (100 MHz). 18

Figure S14. HR-(+) ESI-MS spectrum of 2-hydroxy-6-(1-hydroxy-1-methylpropyl) 3-*sec*-buthylpyrazine (**4**). 19

Figure S15. ^{1}H NMR spectrum of 2-hydroxy-6-(1-hydroxy-1-methylpropyl) 3-*sec*-buthylpyrazine (**4**) in $CDCl_3$ (400 MHz). 20

Figure S16. ^{13}C NMR spectrum of 2-hydroxy-6-(1-hydroxy-1-methylpropyl) 3-*sec*-buthylpyrazine (**4**) in $CDCl_3$ (100 MHz). 21

Figure S17. ^{1}H NMR spectrum of pulchellalactam (**5**) in $CDCl_3$ (400 MHz). 22

Figure S18. ^{13}C NMR spectrum of pulchellalactam (**5**) in $CDCl_3$ (100 MHz). 23

Figure S19. HR-(+) ESI-MS spectrum of monarubin B (**6**). 24

Figure S20. ^{1}H NMR spectrum of monarubin B (**6**) in $CDCl_3$ (400 MHz). 25

Figure S21. ^{13}C NMR spectrum of monarubin B (**6**) in $CDCl_3$ (100 MHz). 26

Figure S22. DEPT 135 spectrum of monarubin B (**6**) in $CDCl_3$ (100 MHz). 27

Figure S23. HMQC spectrum of monarubin B (**6**) in $CDCl_3$. 28

Figure S24. ^{1}H–^{1}H COSY spectrum of monarubin B (**6**) in $CDCl_3$. 29

Figure S25. HMBC spectrum of monarubin B (**6**) in $CDCl_3$. 30

Figure S26. NOESY spectrum of monarubin B (**6**) in $CDCl_3$. 31

Figure S27. ^1H NMR spectrum of lunatinin (**7**) in actone-d_6 (400 MHz). 32

Figure S28. ^{13}C NMR spectrum of lunatinin (**7**) in actone-d_6 (100 MHz). 33

Figure S29. ^1H NMR spectrum of 6,8-dimethoxy-3-methylisocoumarin (**8**) in $CDCl_3$ (400 MHz). 34

Figure S30. ^{13}C NMR spectrum of 6,8-dimethoxy-3-methylisocoumarin (**8**) in $CDCl_3$ (100 MHz). 35

Figure S31. ^1H NMR spectrum of monaspurpurone (**9**) in $CDCl_3$ (400 MHz). 36

Figure S32. ^{13}C NMR spectrum of monaspurpurone (**9**) in $CDCl_3$ (100 MHz). 37

Figure S33. ^1H NMR spectrum of 5-amino-2,6-dimethyl-6-hydroxy-4-(2'-methyl-1-oxobutyl)-3-methoxy-2,4-cyclohexadien-1-one (**10**) in $CDCl_3$ (400 MHz). 38

Figure S34. ^{13}C NMR spectrum of 5-amino-2,6-dimethyl-6-hydroxy-4-(2'-methyl-1-oxobutyl)-3-methoxy-2,4-cyclohexadien-1-one (**10**) in $CDCl_3$ (100 MHz). 39

Figure S35. ^1H NMR spectrum of phomaligol A (**11**) in $CDCl_3$ (400 MHz). 40

Figure S36. ^{13}C NMR spectrum of phomaligol A (**11**) in $CDCl_3$ (100 MHz). 41

Figure S37. ^1H NMR spectrum of monascuspiloin (**12**) in $CDCl_3$ (400 MHz). 42

Figure S38. ^{13}C NMR spectrum of monascuspiloin (**12**) in $CDCl_3$ (100 MHz). 43

Figure S39. HR-(+) ESI-MS spectrum of monarubin C (**13**). 44

Figure S40. ^1H NMR spectrum of monarubin C (**13**) in $CDCl_3$ (600 MHz). 45

Figure S41. ^{13}C NMR spectrum of monarubin C (**13**) in $CDCl_3$ (150 MHz). 46
Figure S42. DEPT 135 spectrum of monarubin C (**13**) in $CDCl_3$ (150 MHz). 47
Figure S43. HMQC spectrum of monarubin C (**13**) in $CDCl_3$. 48
Figure S44. ^1H–^1HCOSY spectrum of monarubin C (**13**) in $CDCl_3$. 49
Figure S45. HMBC spectrum of monarubin C (**13**) in $CDCl_3$. 50
Figure S46. NOESY spectrum of monarubin C (**13**) in $CDCl_3$. 51
Figure S47. The most stable conformers of 11*S*-**6** calculated at the B3LYP/6-31+G(d) level. Relative populations are in parentheses. 52
Figure S48. The most stable conformers of 11*R*-**6** calculated at the B3LYP/6-31+G(d) level. Relative populations are in parentheses. 53
Figure S49. The most stable conformers of 3*S*,6*R*,7*S*,11*S*,17*R*-**13** calculated at the B3LYP/6-31+G(d) level. Relative populations are in parentheses. 54
Figure S50. The most stable conformers of 3*S*,6*R*,7*S*,11*S*,17*S*-**13** calculated at the B3LYP/6-31+G(d) level. Relative populations are in parentheses. 55
Figure S51. The most stable conformers of 3*R*,6*S*,7*R*,11*R*,17*S*-**13** calculated at the B3LYP/6-31+G(d) level. Relative populations are in parentheses. 56
Figure S52. The most stable conformers of 3*R*,6*S*,7*R*,11*R*,17*R*-**13** calculated at the B3LYP/6-31+G(d) level. Relative populations are in parentheses. 57
Figure S53. The most stable conformers of 11*S*-**3** calculated at the B3LYP/6-31+G(d) level. Relative populations are in parentheses. 58

Figure S54. The most stable conformers of 11*R*-**3** calculated at the B3LYP/6-31+G(d) level. Relative populations are in parentheses. 59

Table S1. X-ray Crystallography Data for **4**. 60

Table S2.The hepatocellular carcinoma cell line HepG2 activity of manorubin B (**6**)... 62

Table S3. The hepatocellular carcinoma cell line QGY7701 activity of manorubin B (**6**)... 62

Table S4. The nasopharyngeal carcinoma cell line SUNE1 activity of lunatinin (**7**) 63

Table S5. The hepatocellular carcinoma cell line HepG2 activity of lunatinin (**7**)... 63

Table S6. The hepatocellular carcinoma cell line QGY7701 activity of lunatinin (**7**) 63

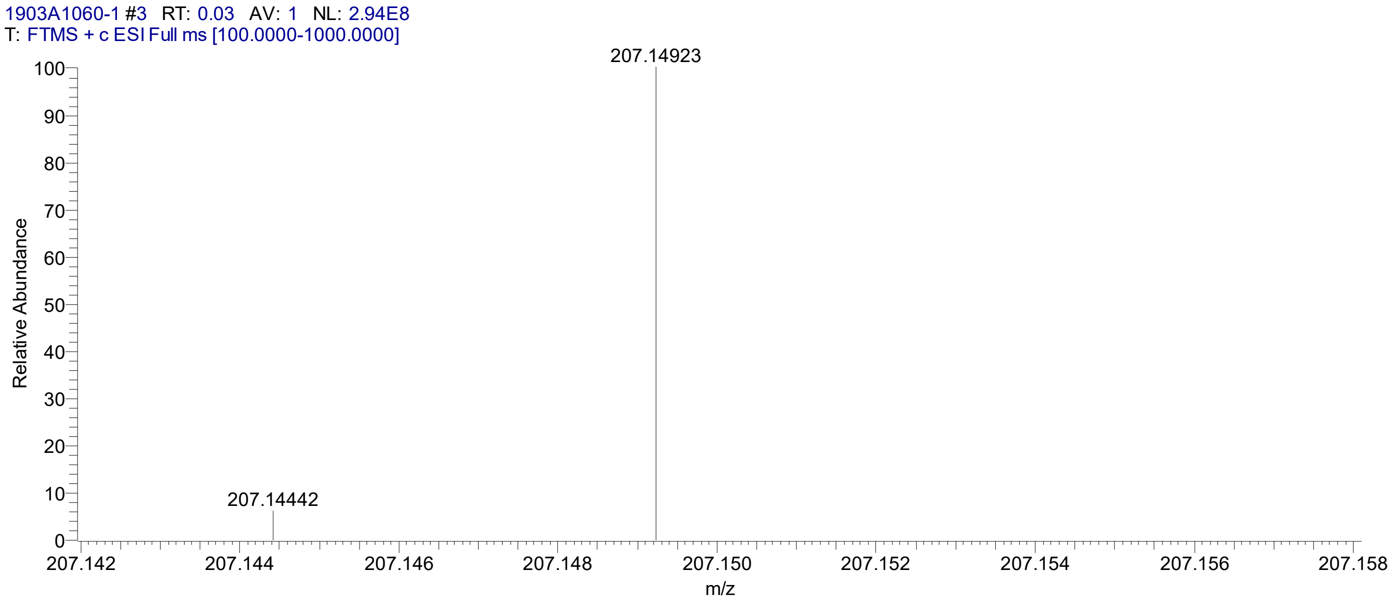

SPECTRUM-simulation :

m/z	Theo. Mass	Delta (ppm)	RDB equiv.	Composition
207.14923	207.14919	0.19	4.5	C12 H19 O N2

Figure S1. HR-(+) ESI-MS spectrum of monarubin A (**1**).

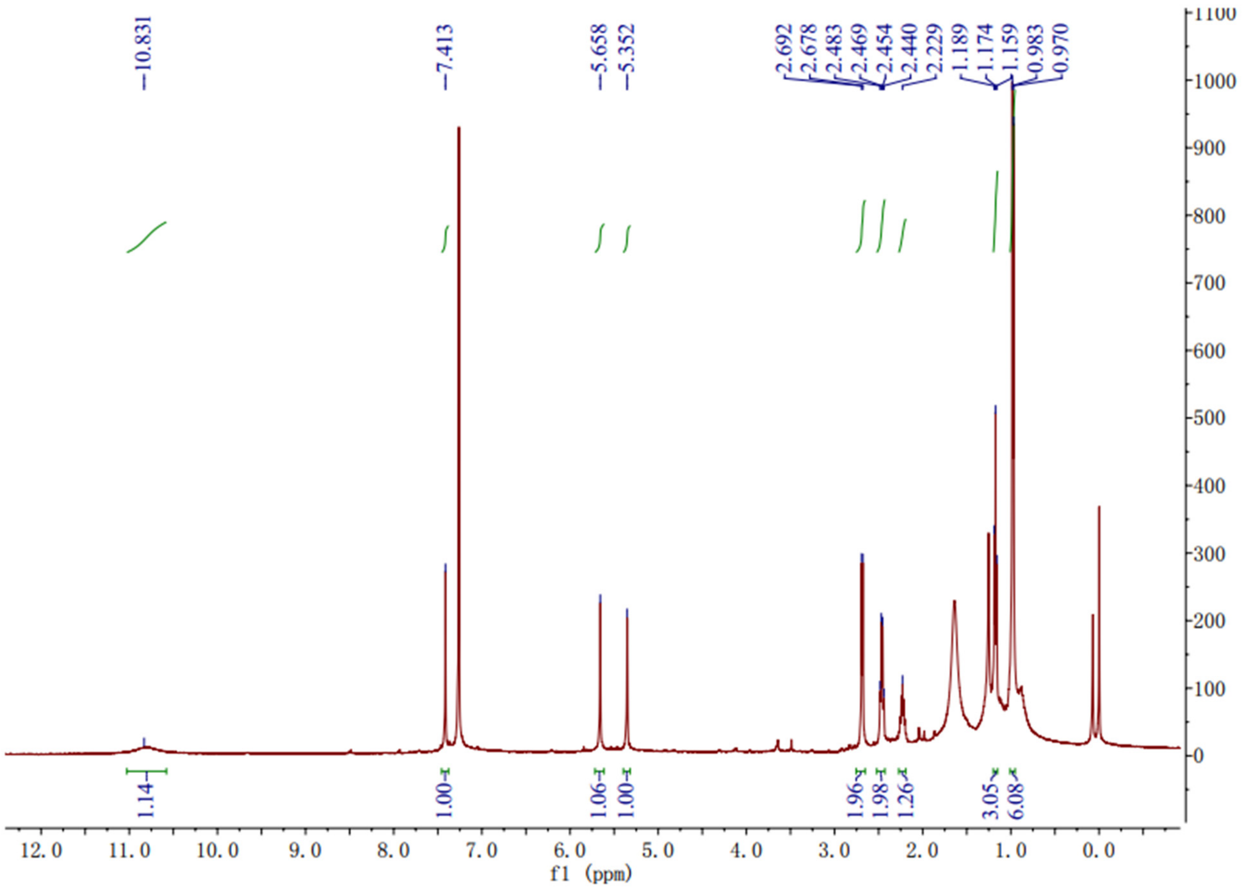

Figure S2. ^{1}H NMR spectrum of monarubin A (**1**) in $CDCl_3$ (500 MHz).

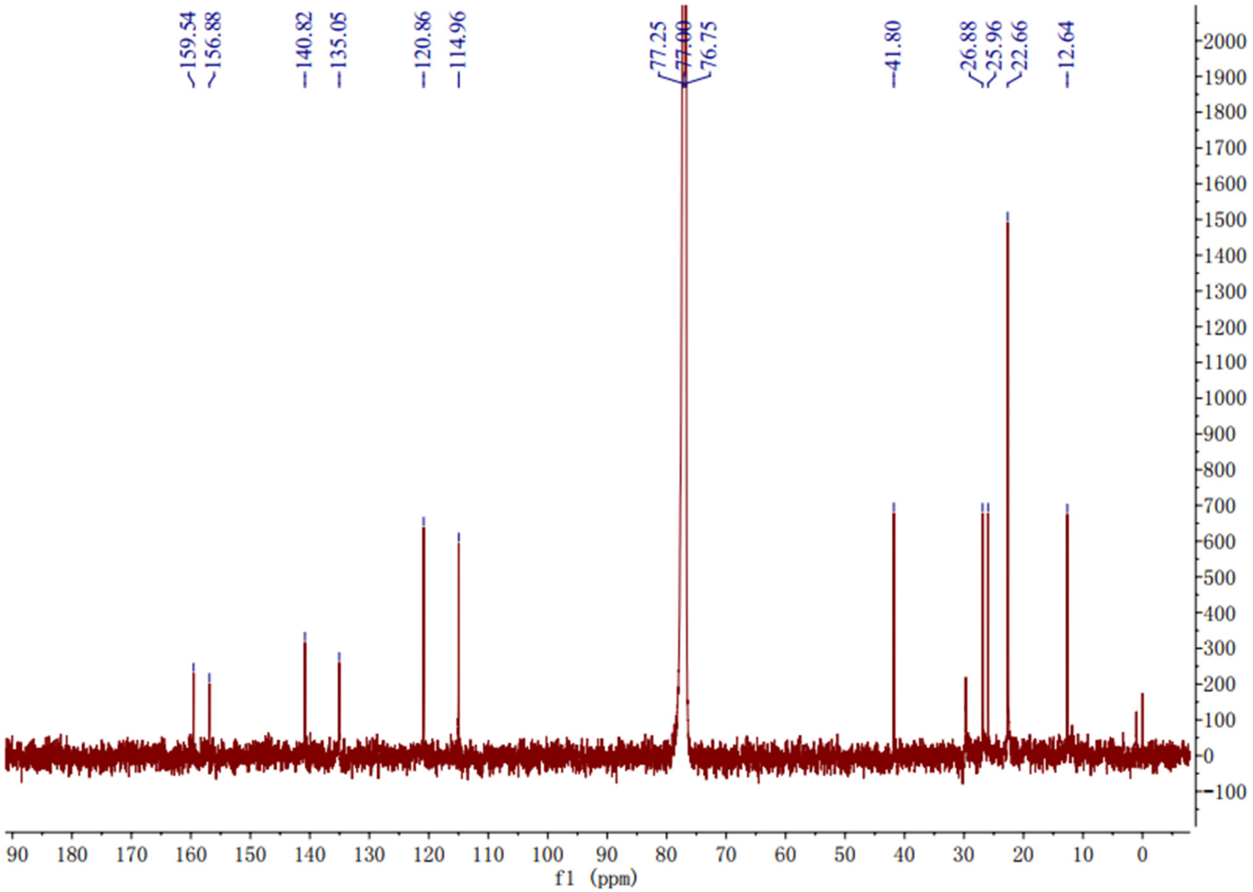

Figure S3. ^{13}C NMR spectrum of monarubin A (**1**) in $CDCl_3$ (125 MHz).

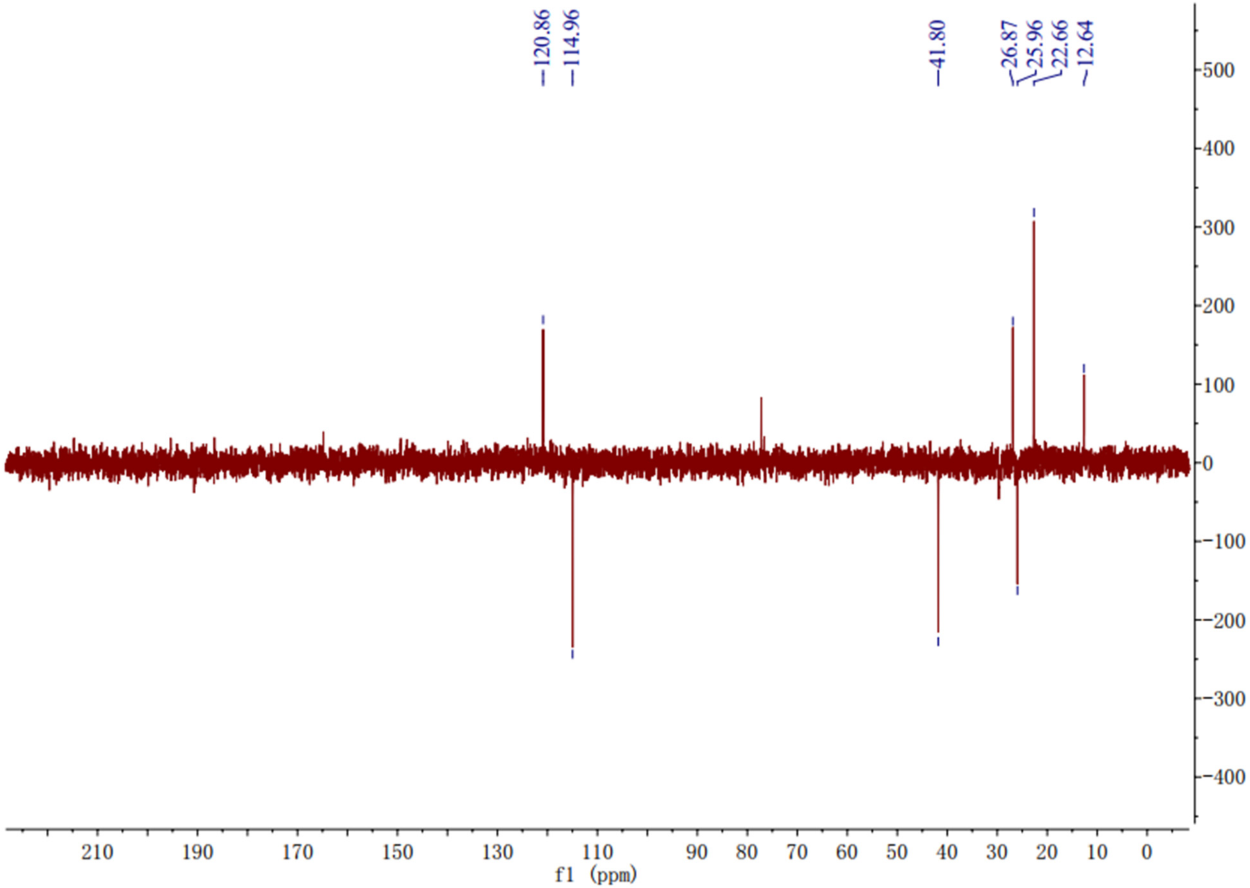

Figure S4. DPET 135 spectrum of monarubin A (**1**) in $CDCl_3$ (125 MHz).

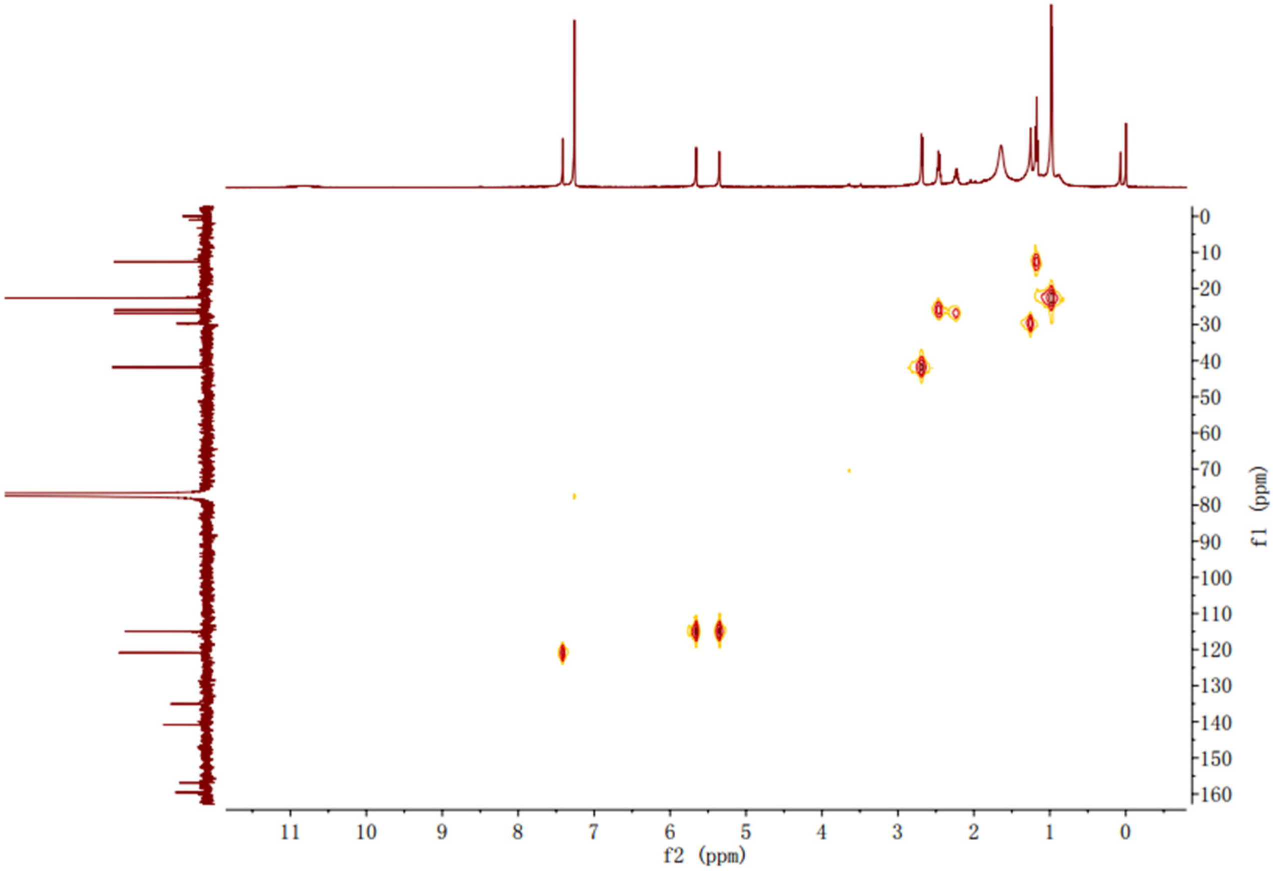

Figure S5. HMQC spectrum of monarubin A (**1**) in $CDCl_3$.

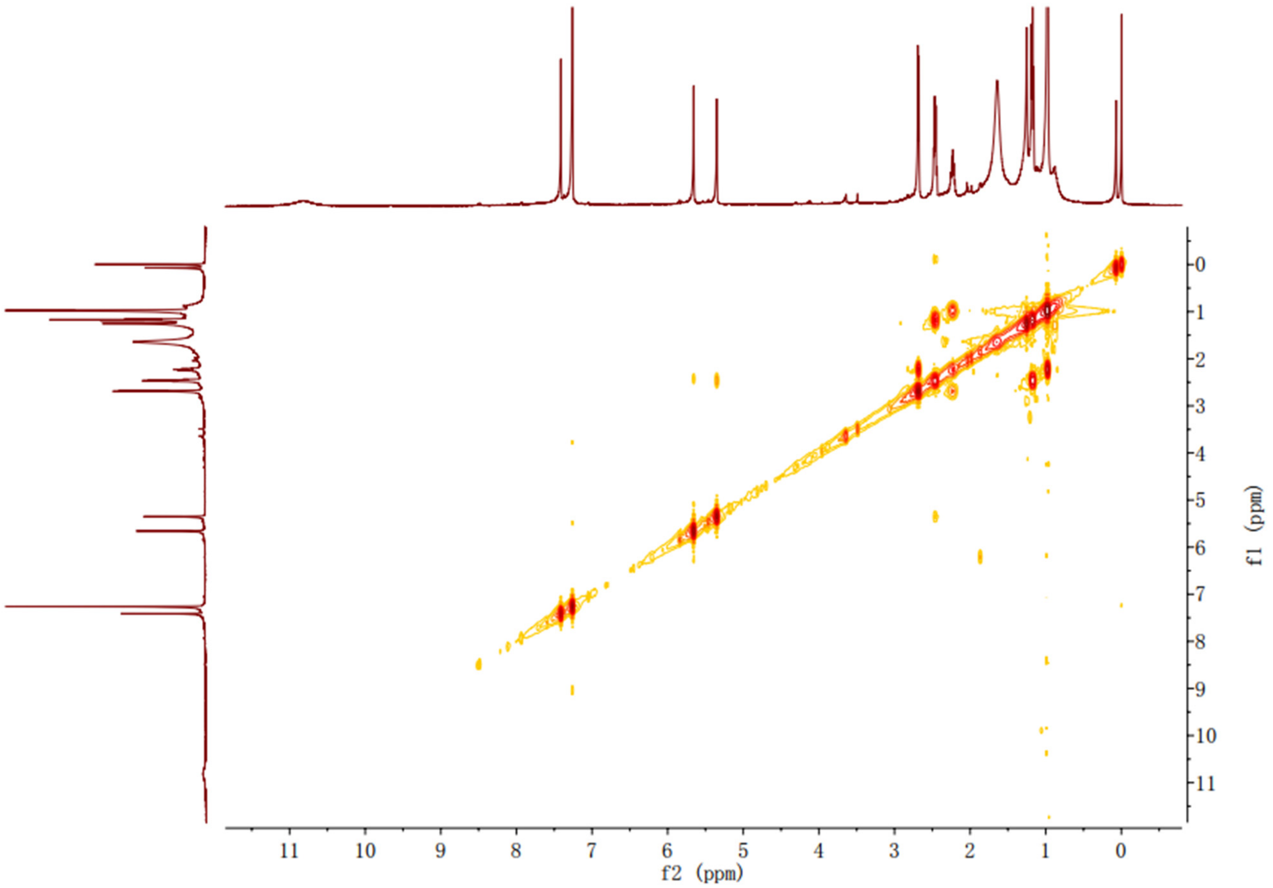

Figure S6. ^{1}H–^{1}HCOSY spectrum of monarubin A (**1**) in $CDCl_3$.

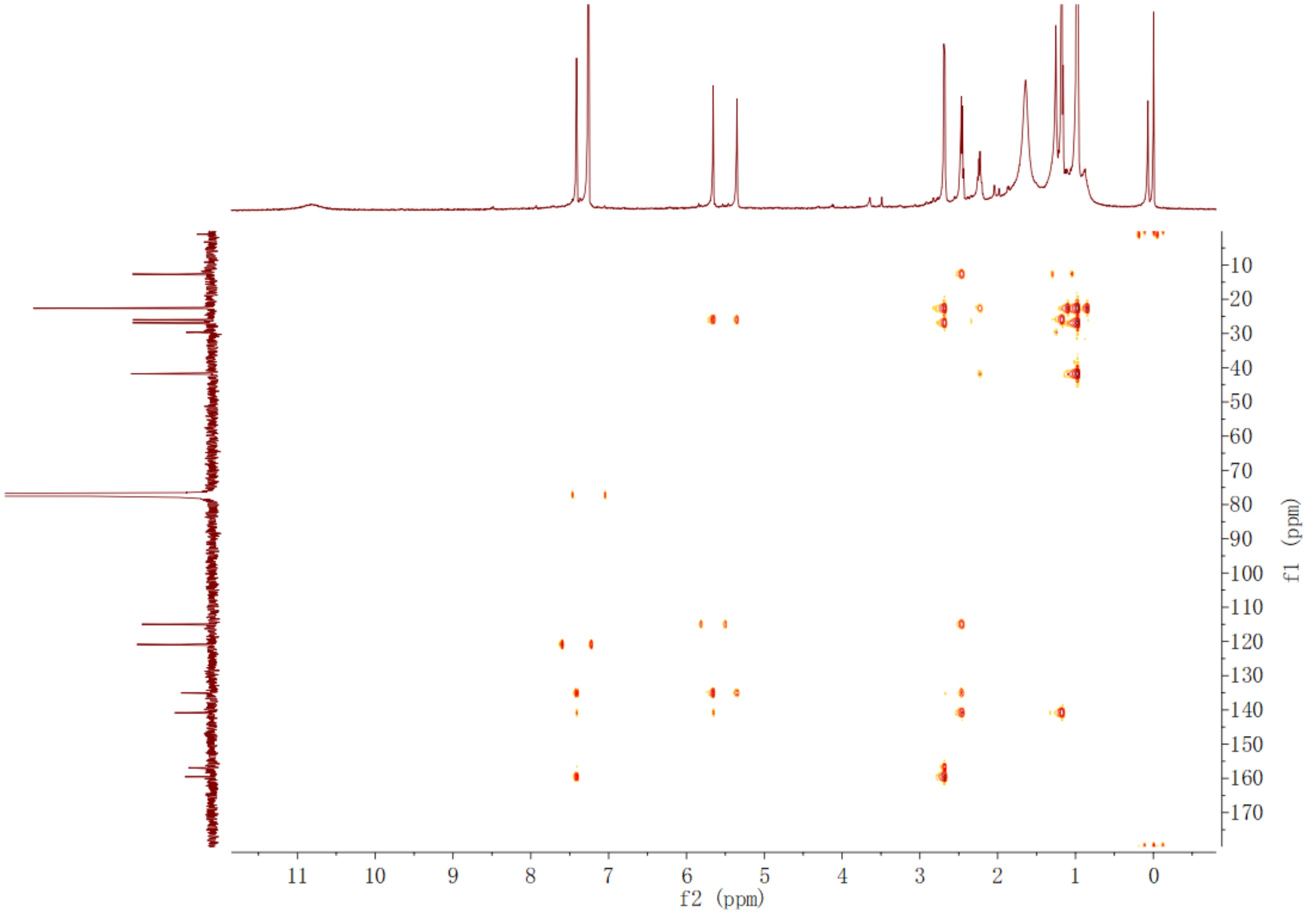

Figure S7. HMBC spectrum of monarubin A (**1**) in $CDCl_3$.

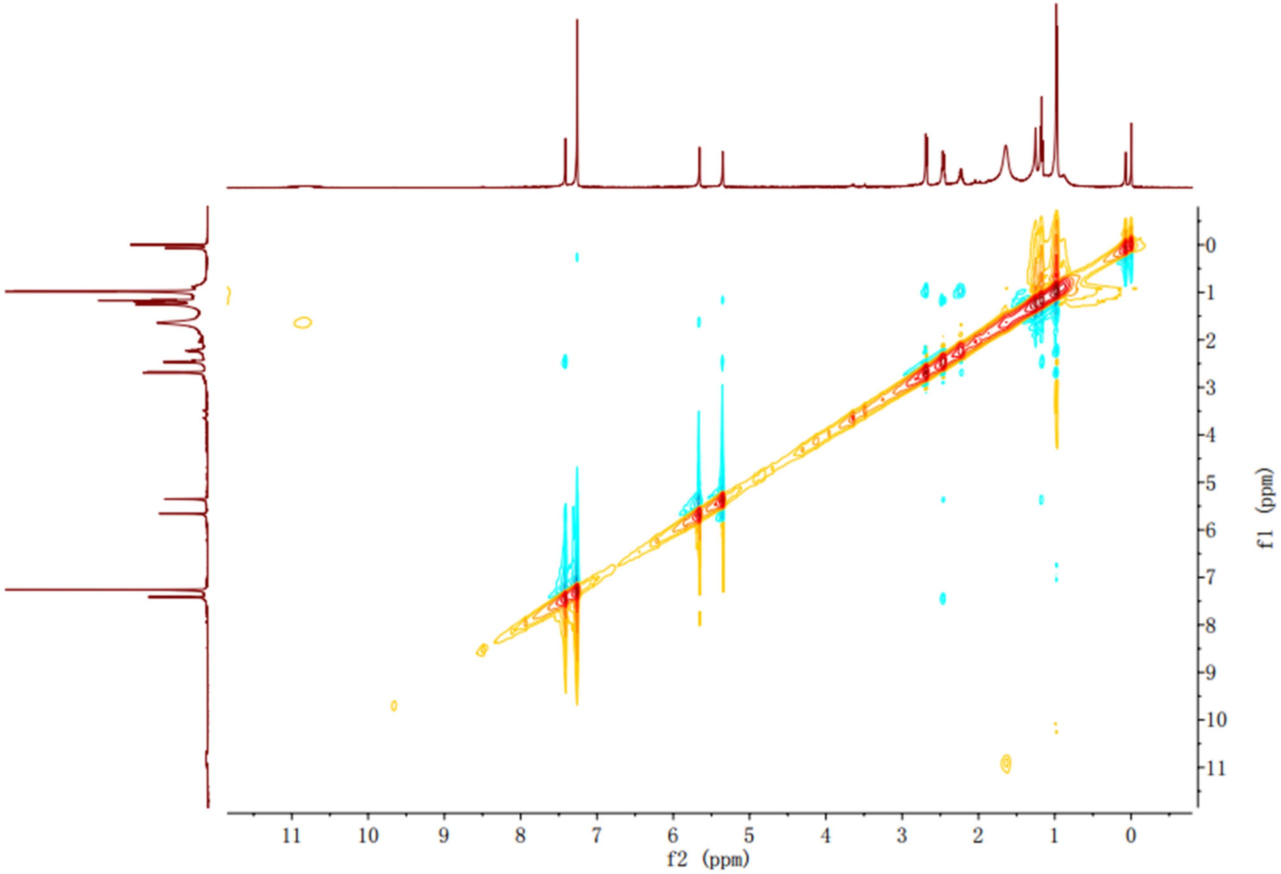

Figure S8. NOEY spectrum of monarubin A (**1**) in $CDCl_3$.

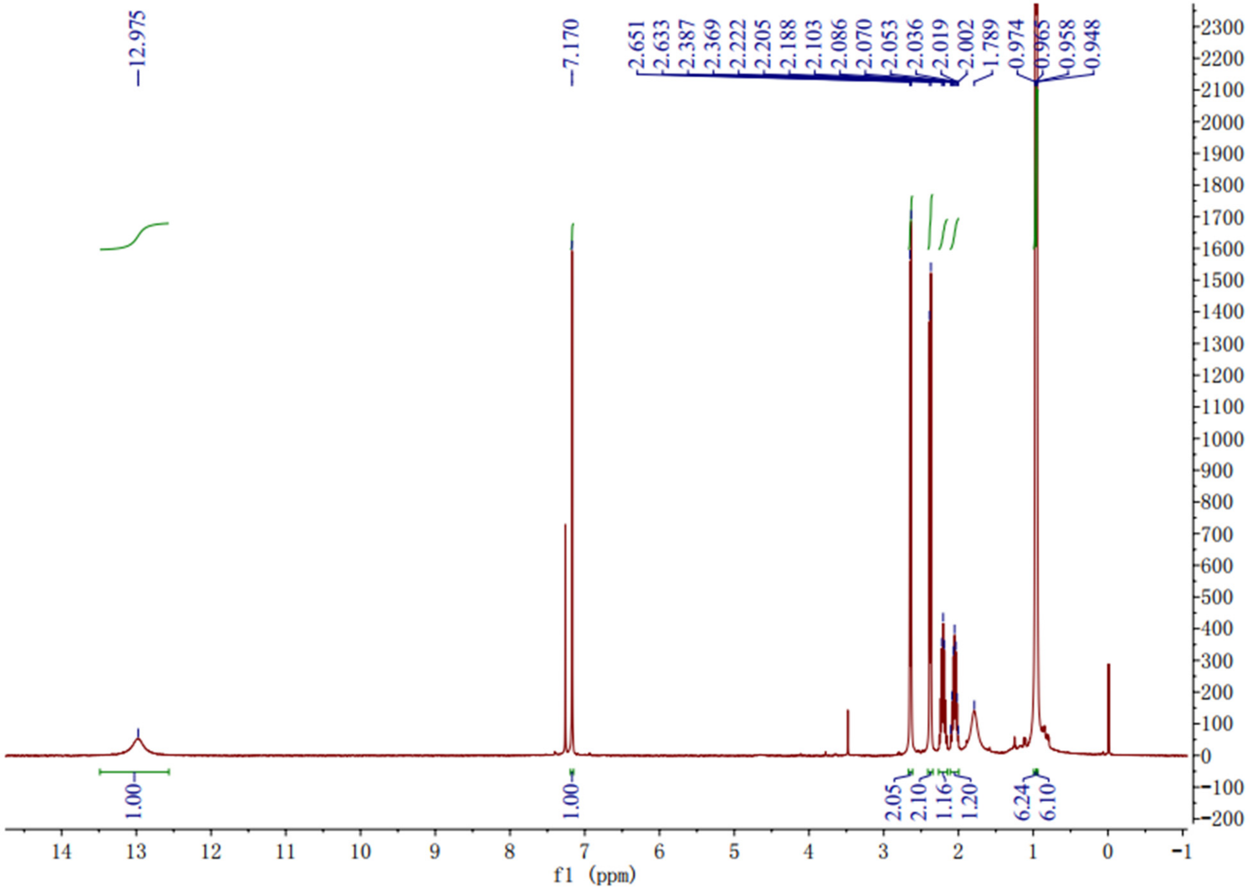

Figure S9. ^{1}H NMR spectrum of 3,6-diisobutyl-2(1*H*)-pyrazinone (**2**) in $CDCl_3$ (400 MHz).

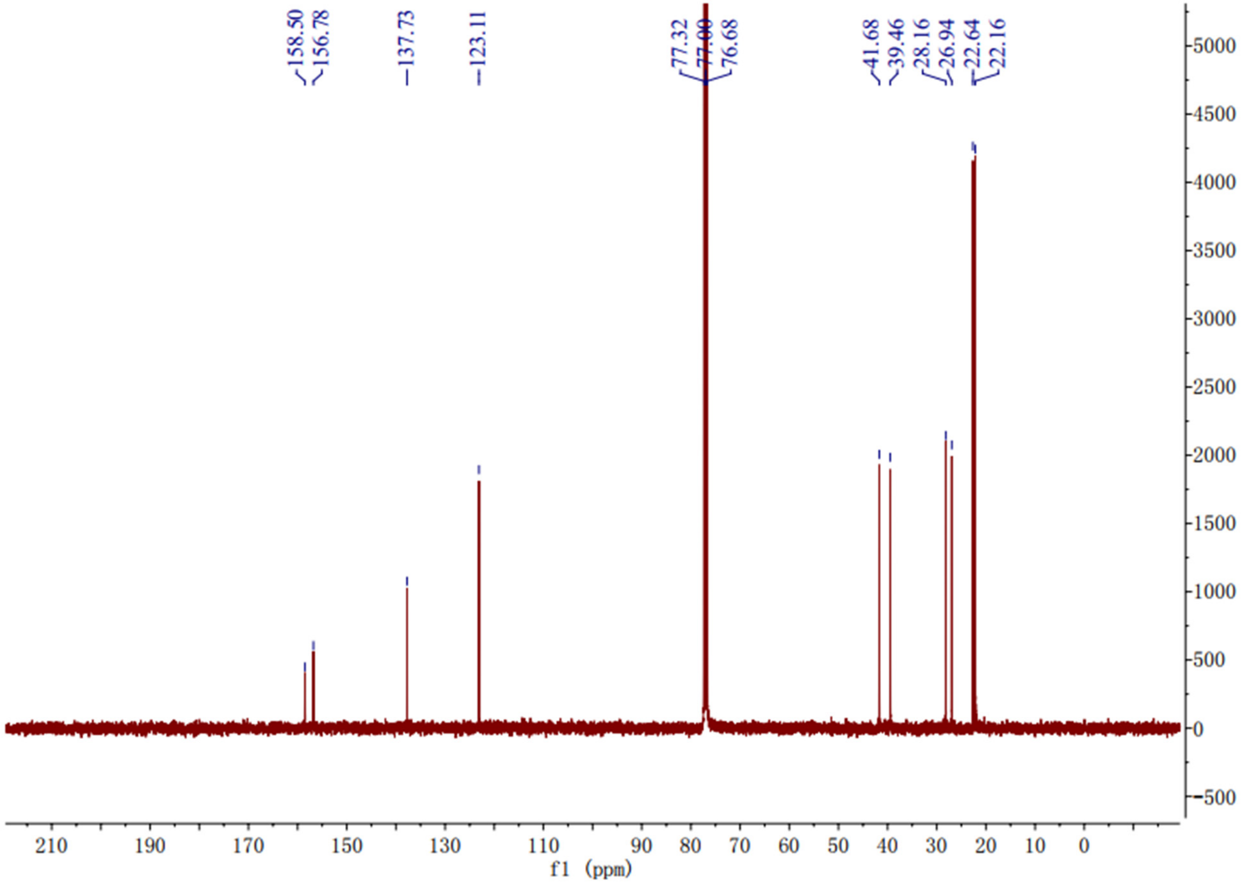

Figure S10. ^{13}C NMR spectrum of 3,6-diisobutyl-2(1*H*)-pyrazinone (**2**) in $CDCl_3$ (100 MHz).

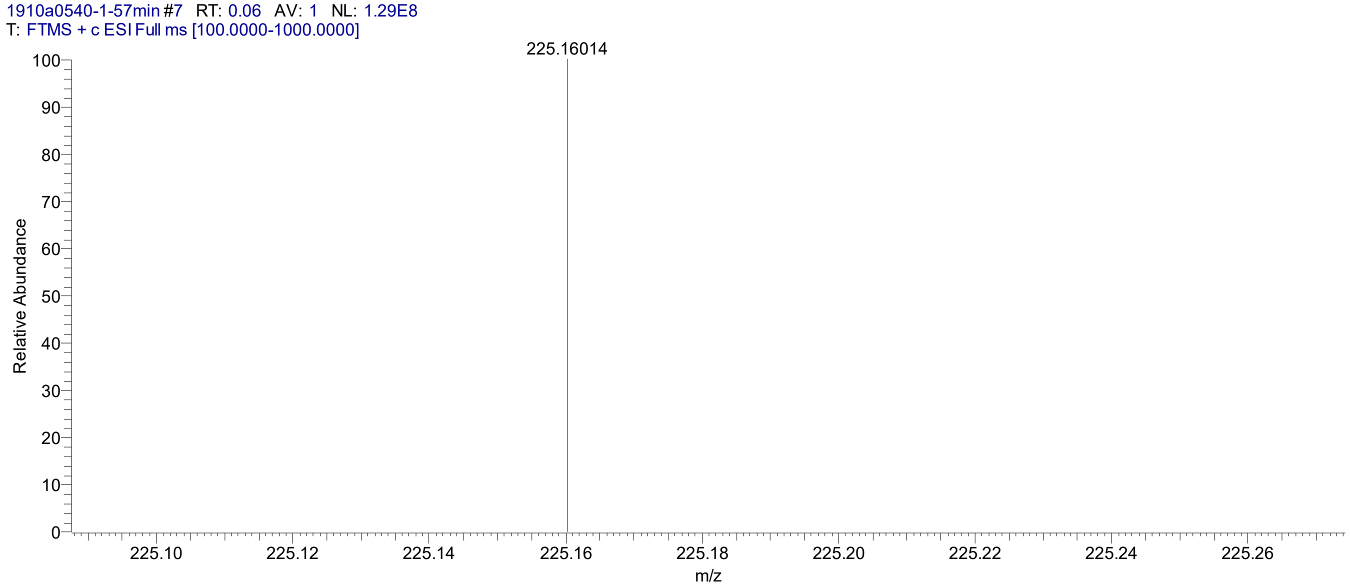

SPECTRUM-simulation :				
m/z	Theo. Mass	Delta (ppm)	RDB equiv.	Composition
225.16019	225.15975	1.93	3.5	C12 H21 O2 N2

Figure S11. HR-(+) ESI-MS spectrum of deoxyhydroxyaspergillic acid (**3**).

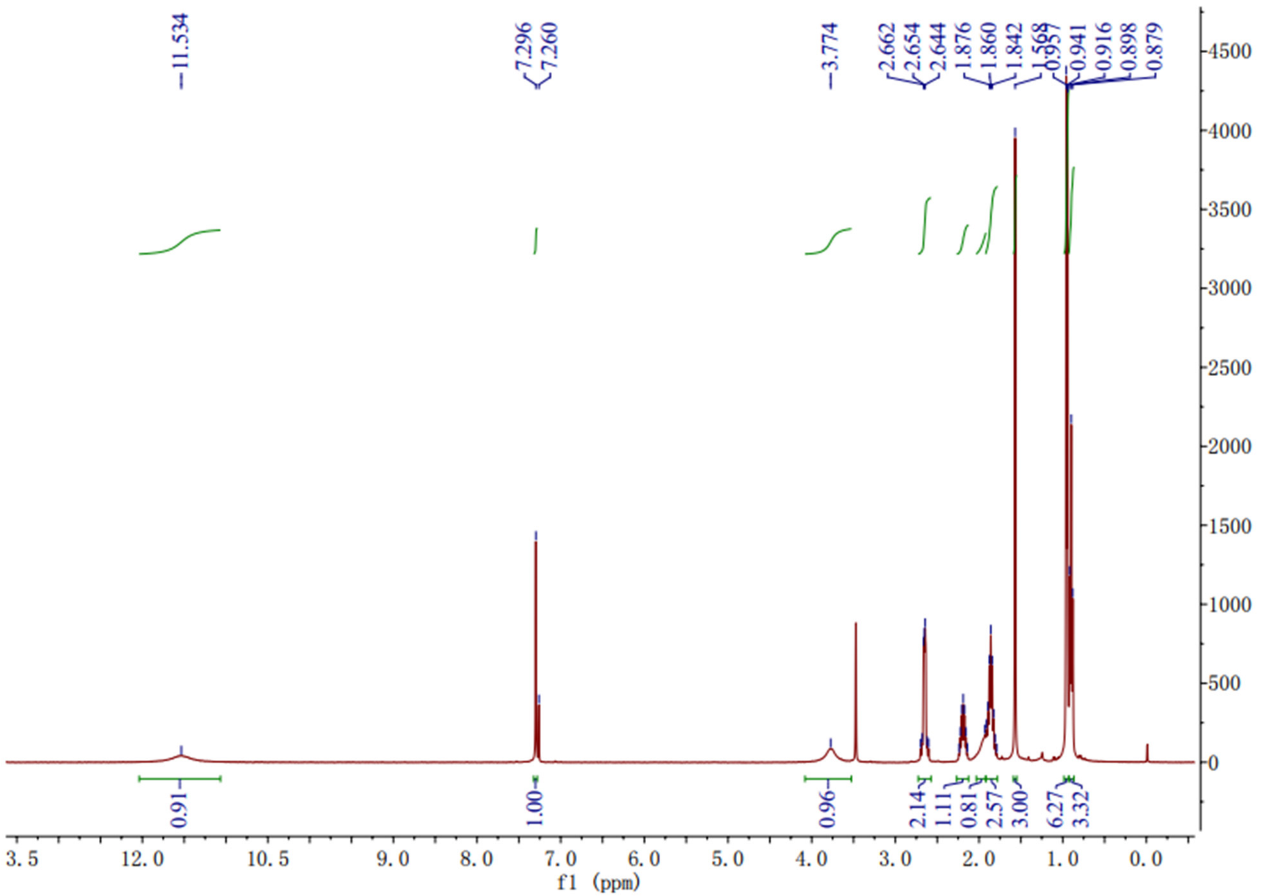

Figure S12. ^{1}H NMR spectrum of deoxyhydroxyaspergillic acid (**3**) in $CDCl_3$ (400 MHz).

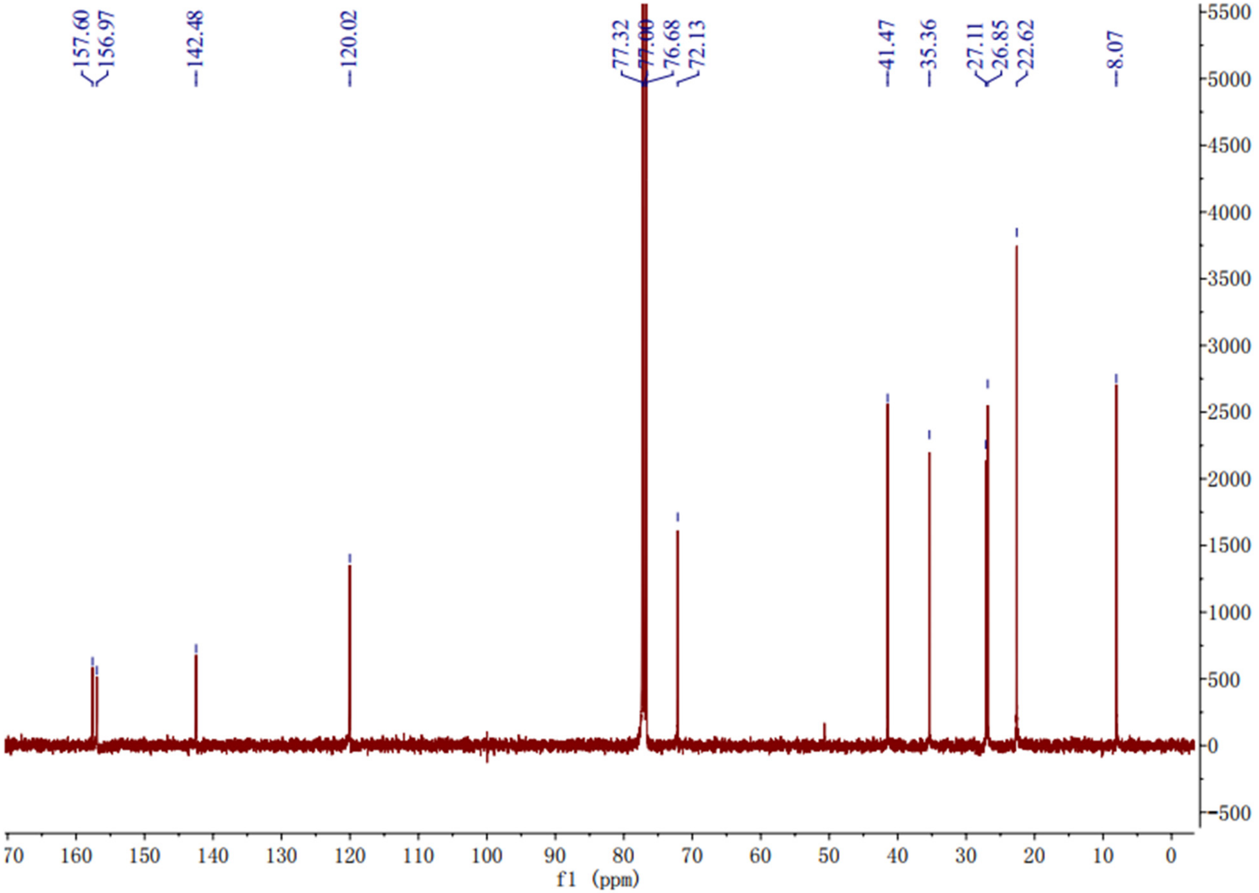

Figure S13. ^{13}C NMR spectrum of deoxyhydroxyaspergillic acid (**3**) in $CDCl_3$ (100 MHz).

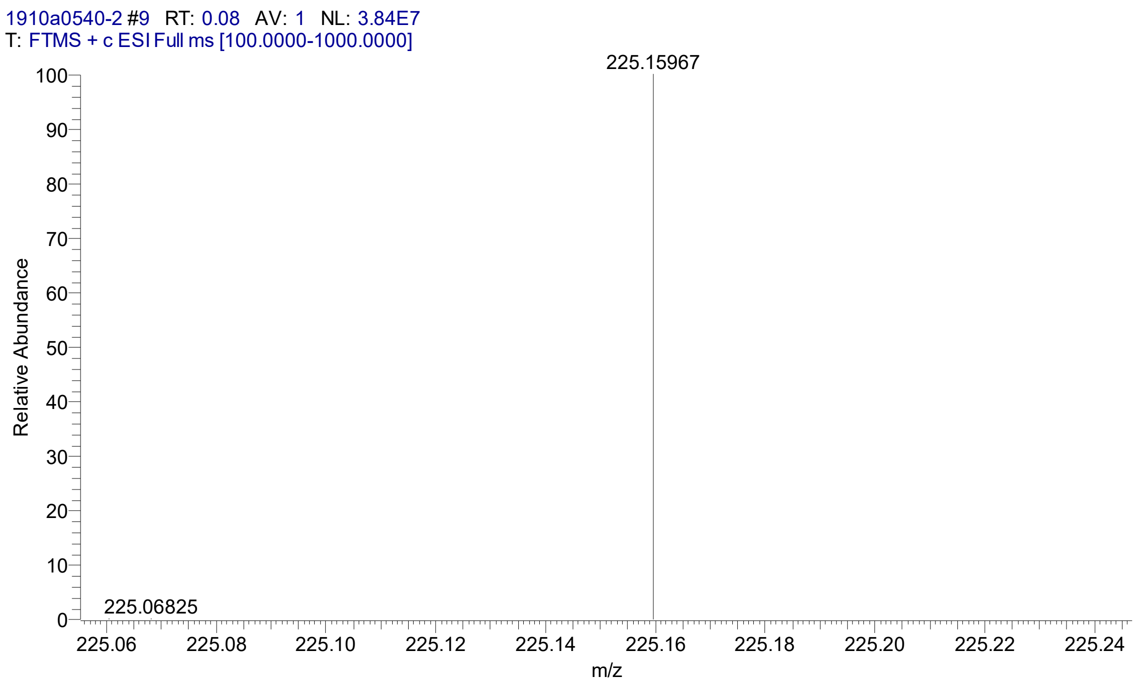

SPECTRUM-simulation :

m/z	Theo. Mass	Delta (ppm)	RDB equiv.	Composition
225.15967	225.15975	-0.37	3.5	C12 H21 O2 N2

Figure S14. HR-(+) ESI-MS spectrum of 2-hydroxy-6-(1-hydroxy-1-methylpropyl) 3-*sec*-buthylpyrazine (**4**).

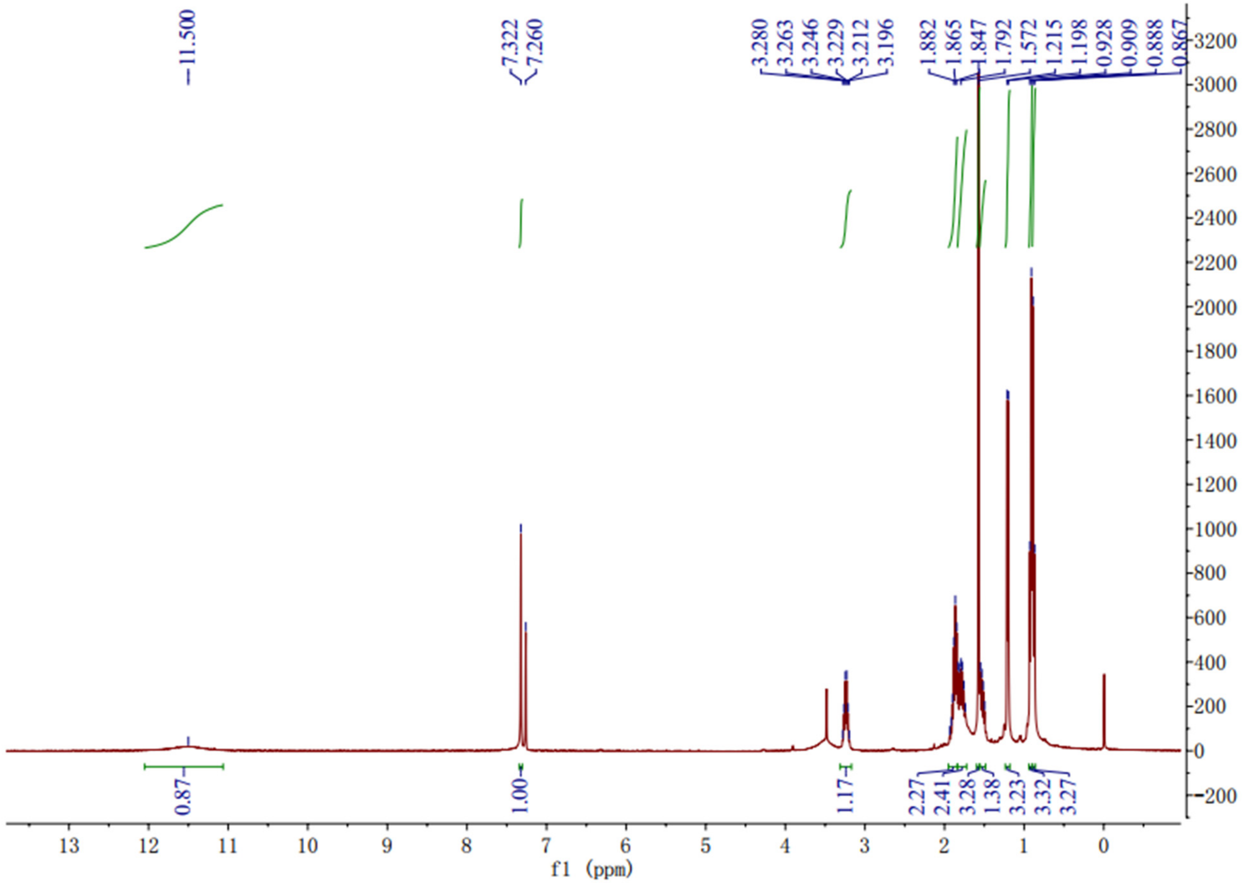

Figure S15. ^{1}H NMR spectrum of 2-hydroxy-6-(1-hydroxy-1-methylpropyl) 3-*sec*-buthylpyrazine (**4**) in $CDCl_3$ (400 MHz).

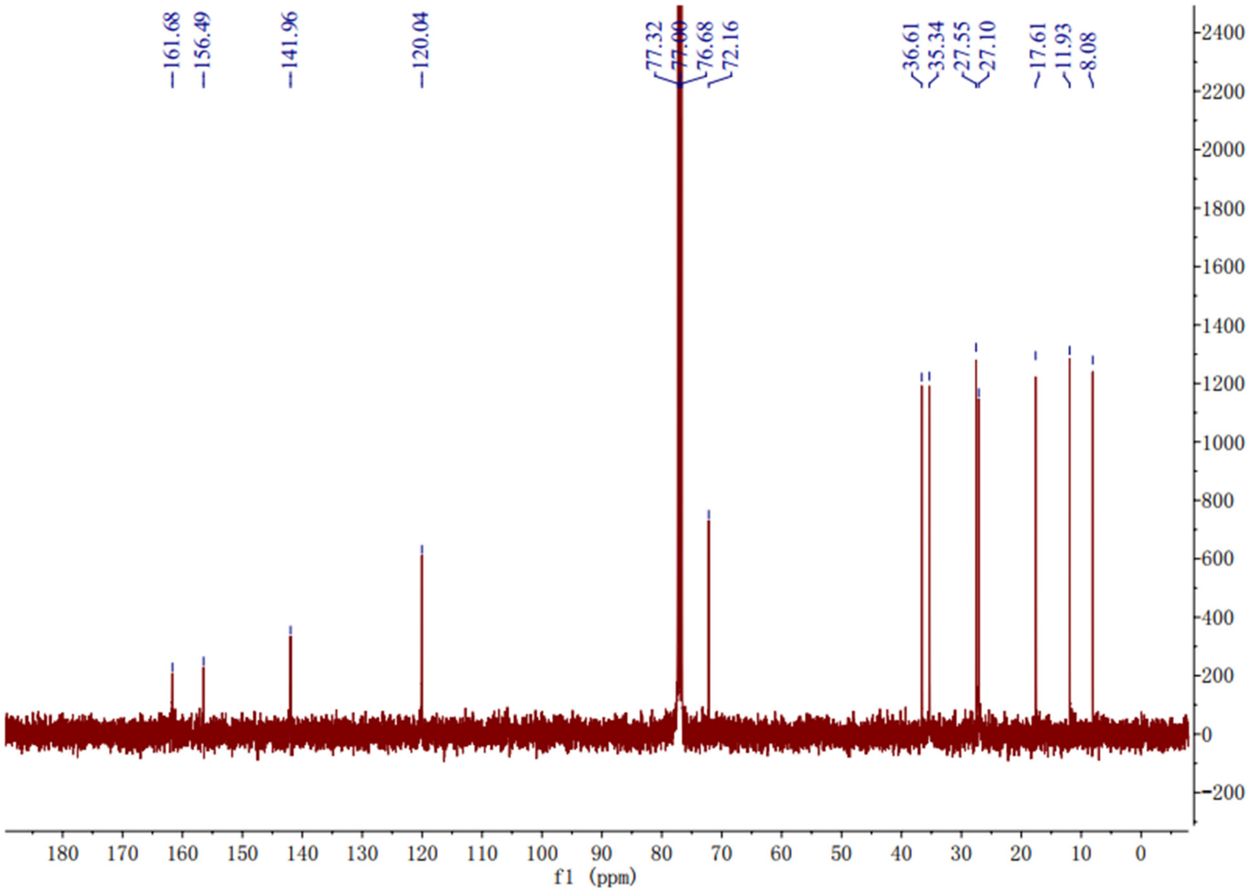

Figure S16. ^{13}C NMR spectrum of 2-hydroxy-6-(1-hydroxy-1-methylpropyl) 3-*sec*-buthylpyrazine (**4**) in $CDCl_3$ (100 MHz).

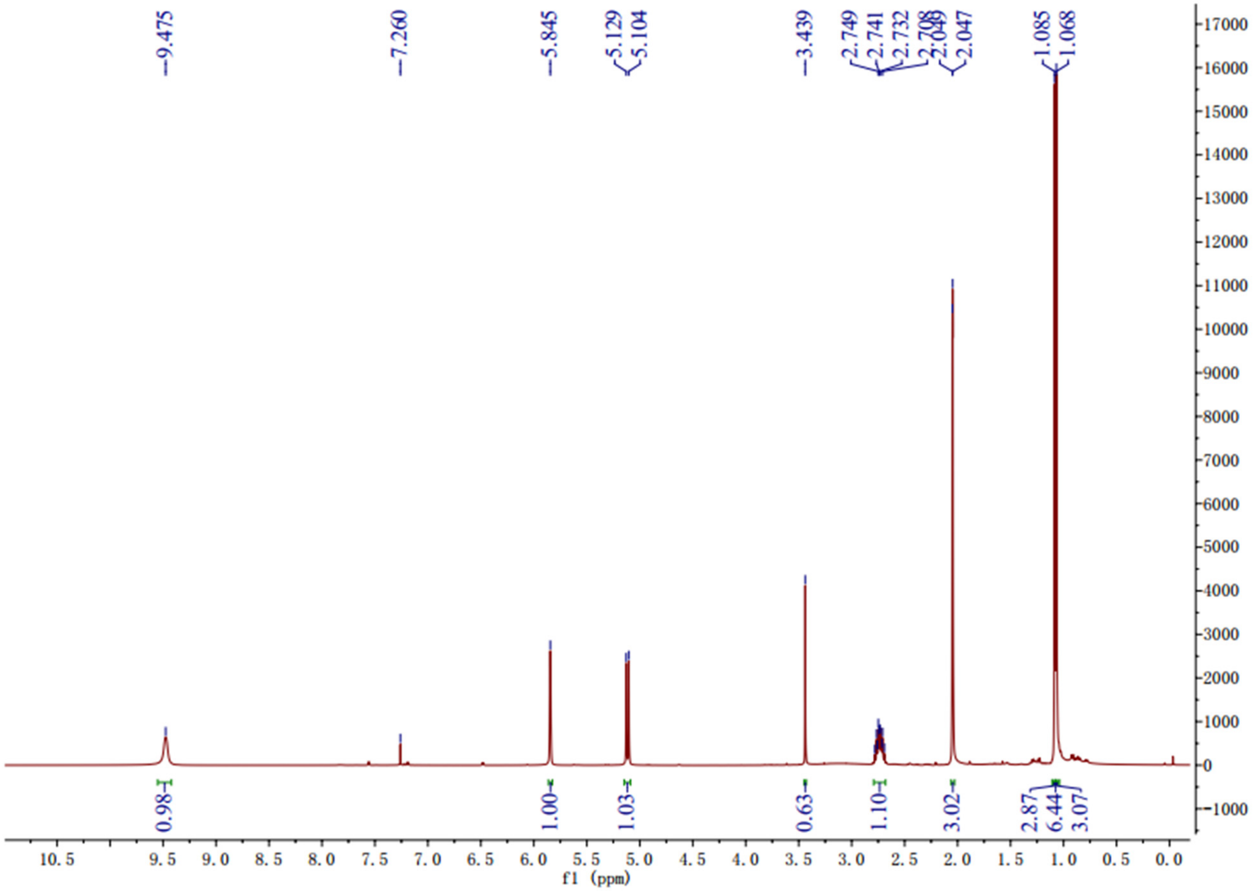

Figure S17. ^{1}H NMR spectrum of pulchellalactam (**5**) in $CDCl_3$ (400 MHz).

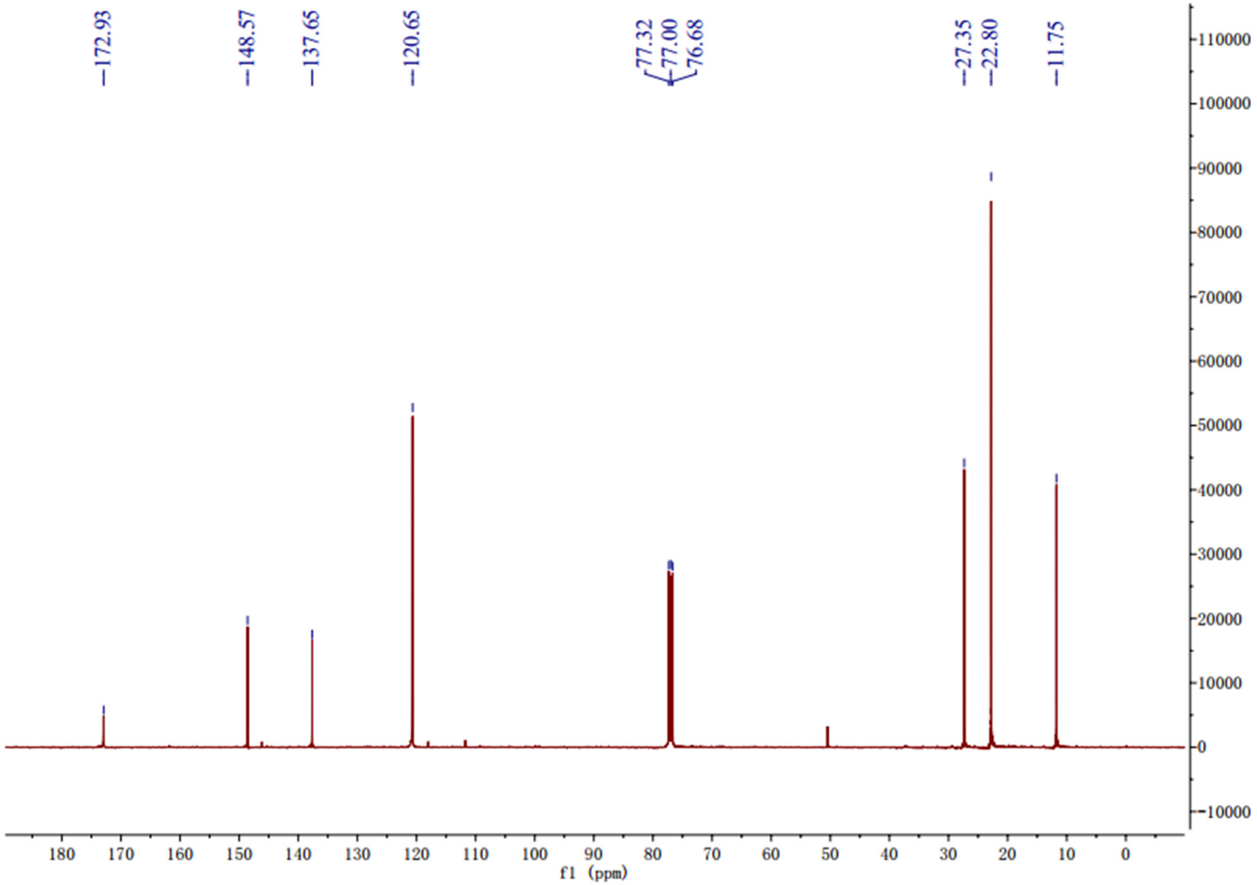

Figure S18. ^{13}C NMR spectrum of pulchellalactam (**5**) in $CDCl_3$ (100 MHz).

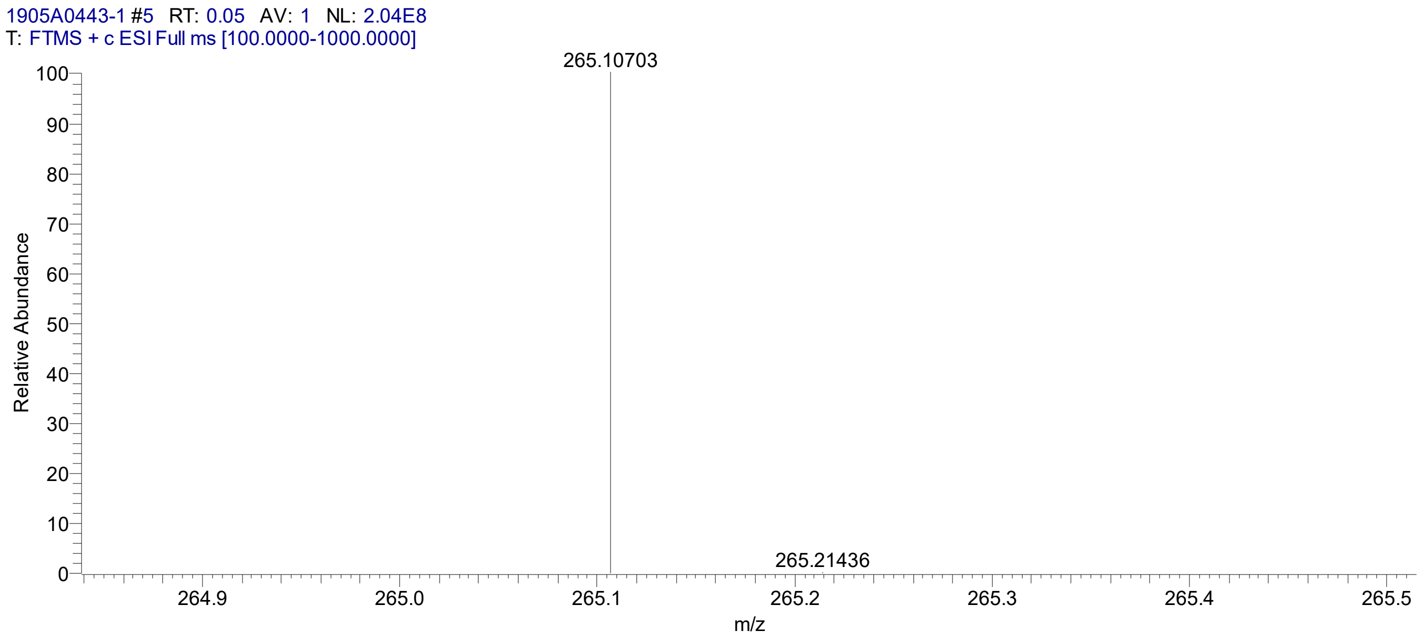

SPECTRUM - simulation :

m/z	Theo. Mass	Delta (ppm)	RDB equiv.	Composition
265.10703	265.10705	-0.08	6.5	C14 H17 O5

Figure S19. HR-(+) ESI-MS spectrum of monarubin B (**6**).

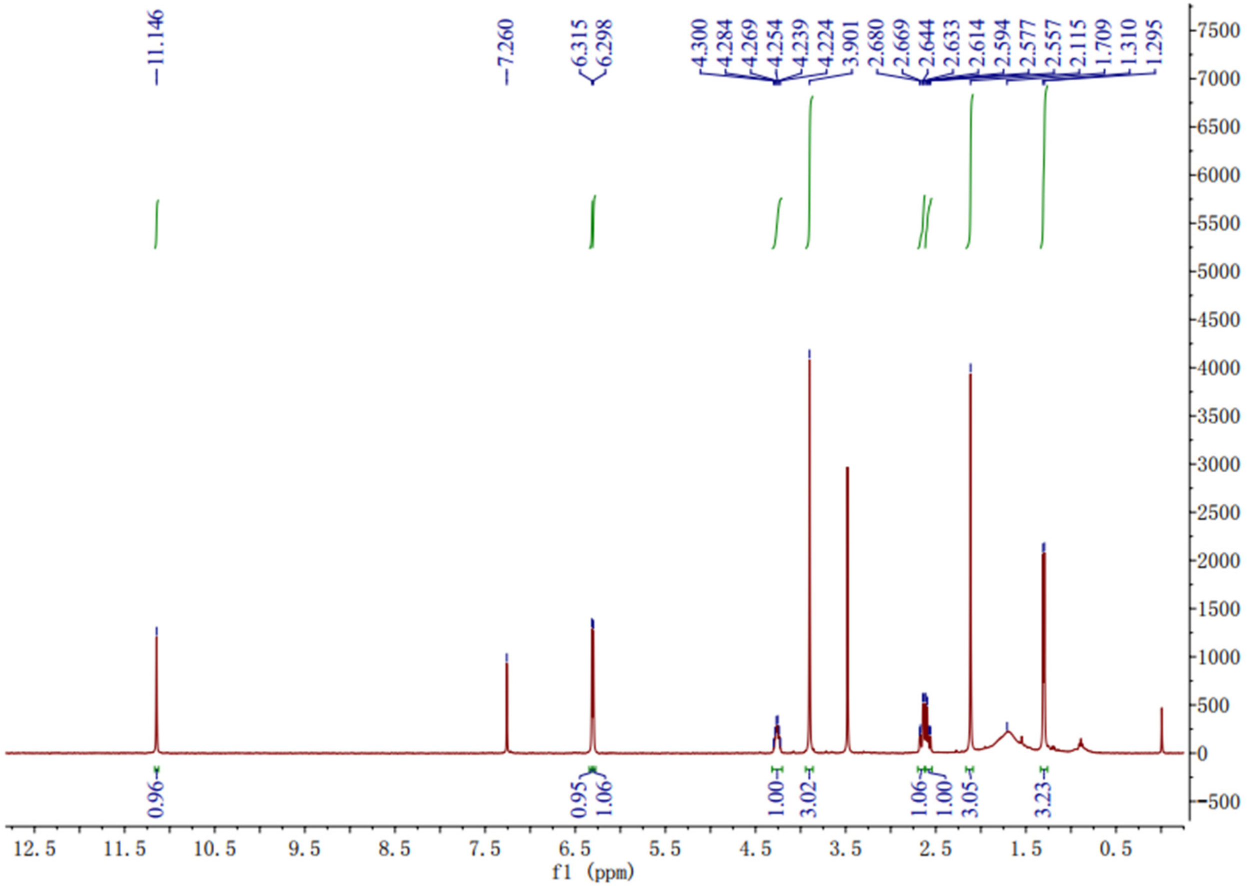

Figure S20. ^{1}H NMR spectrum of monarubin B (**6**) in $CDCl_3$ (400 MHz).

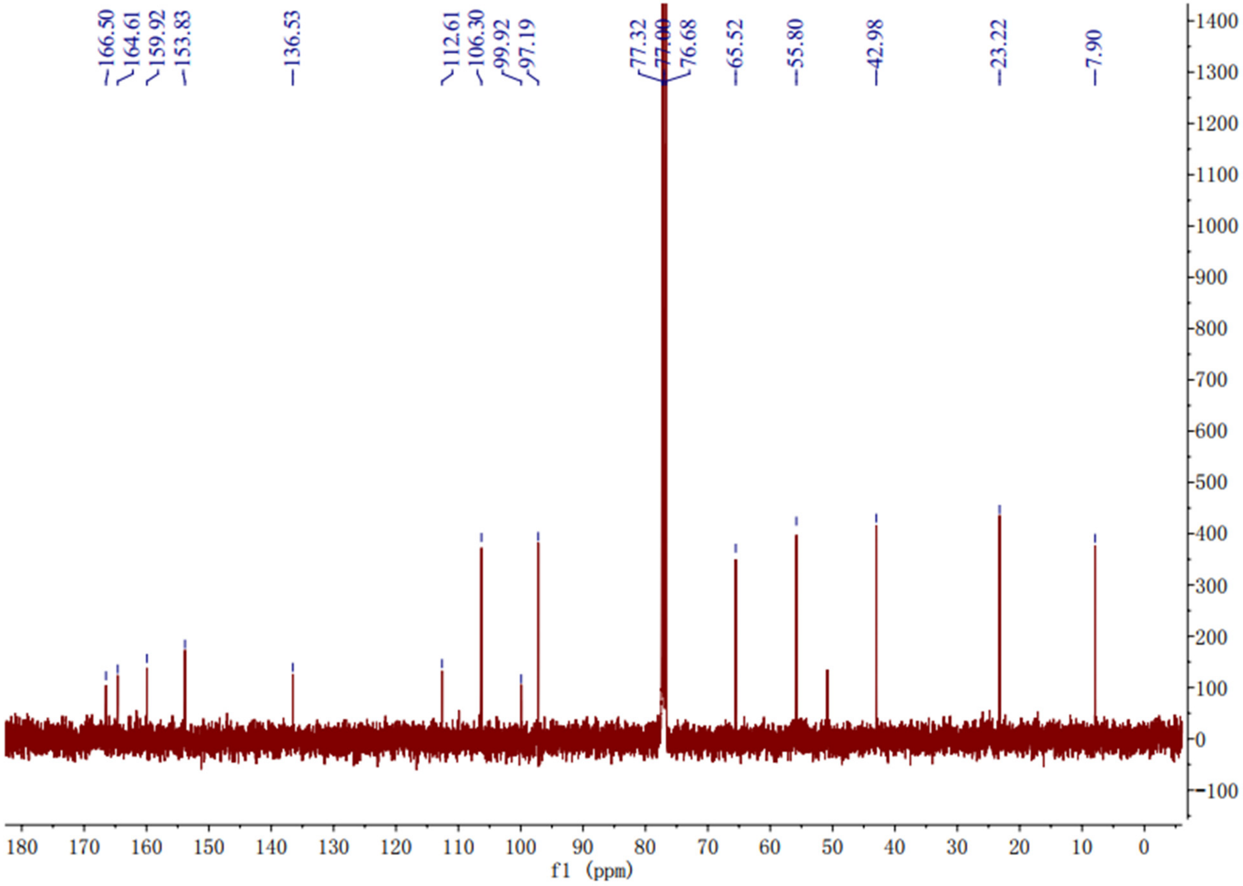

Figure S21. ^{13}C NMR spectrum of monarubin B (**6**) in $CDCl_3$ (100 MHz).

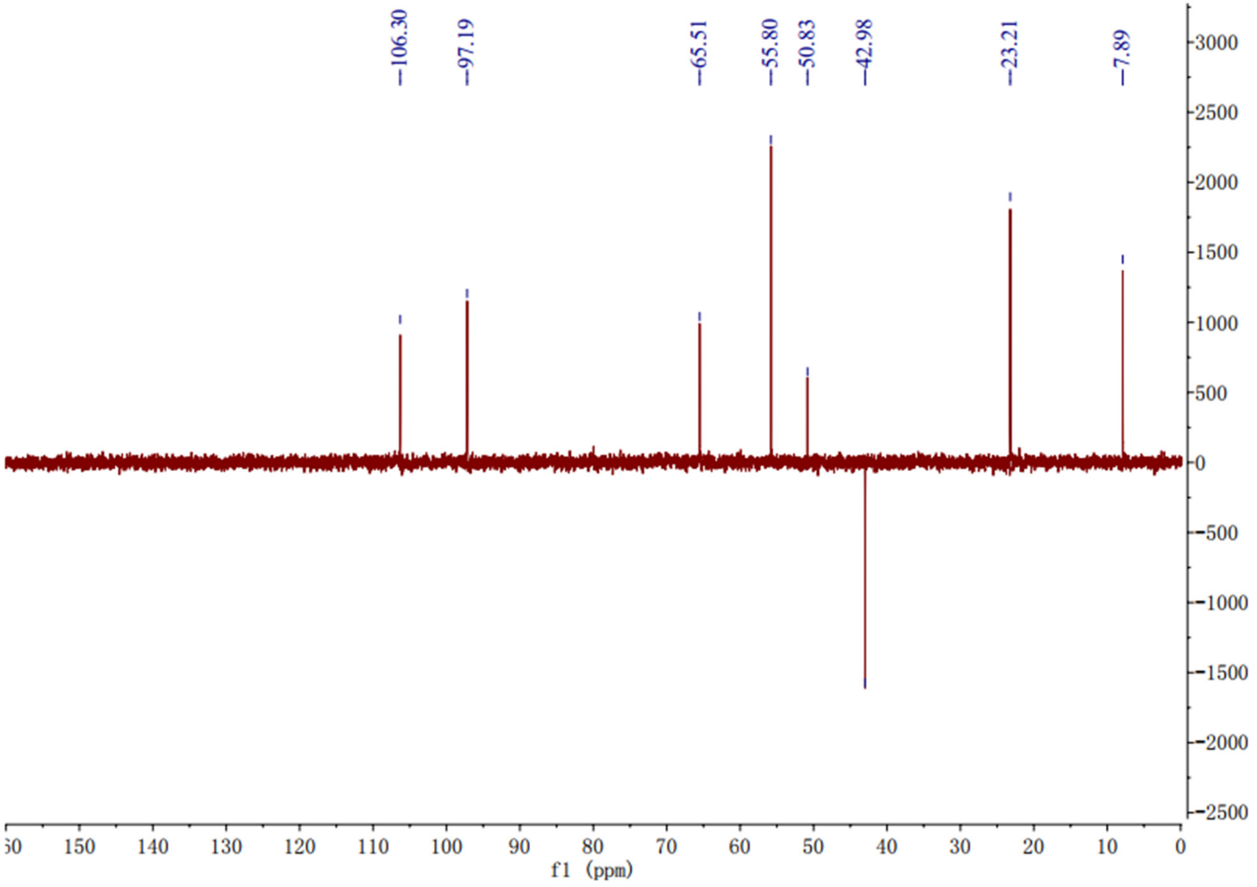

Figure S22. DEPT 135 spectrum of monarubin B (**6**) in $CDCl_3$ (100 MHz).

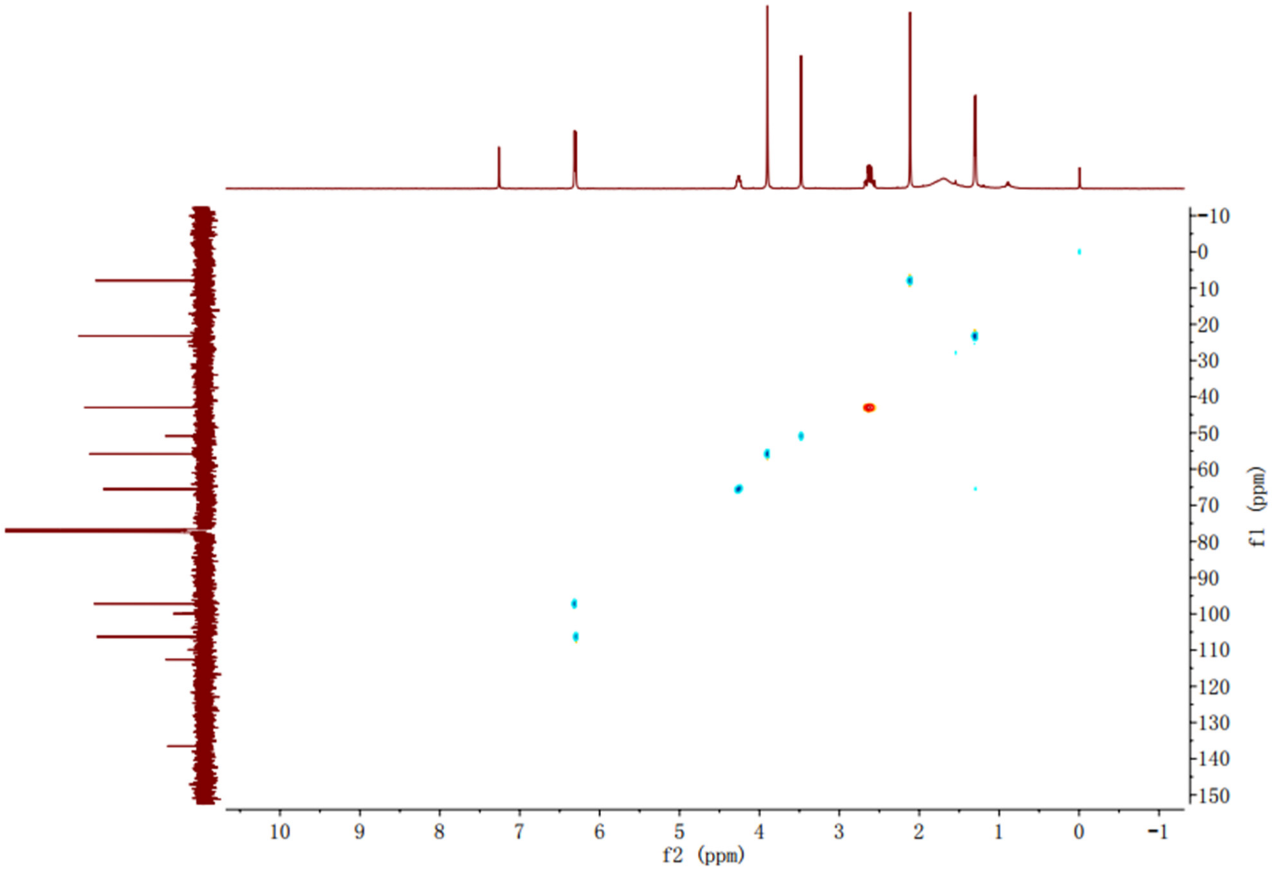

Figure S23. HMQC spectrum of monarubin B (**6**) in $CDCl_3$.

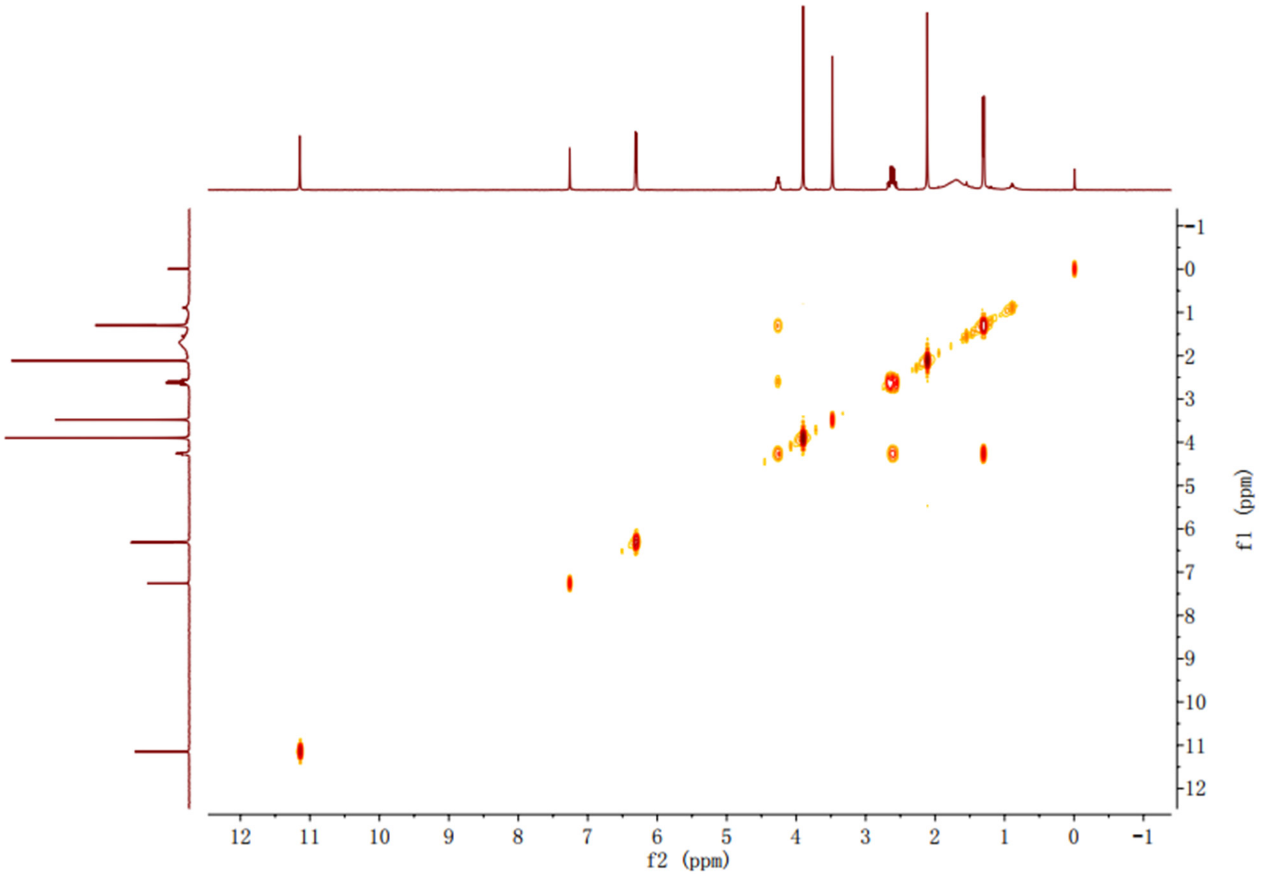

Figure S24. ^{1}H–^{1}H COSY spectrum of monarubin B (**6**) in $CDCl_3$.

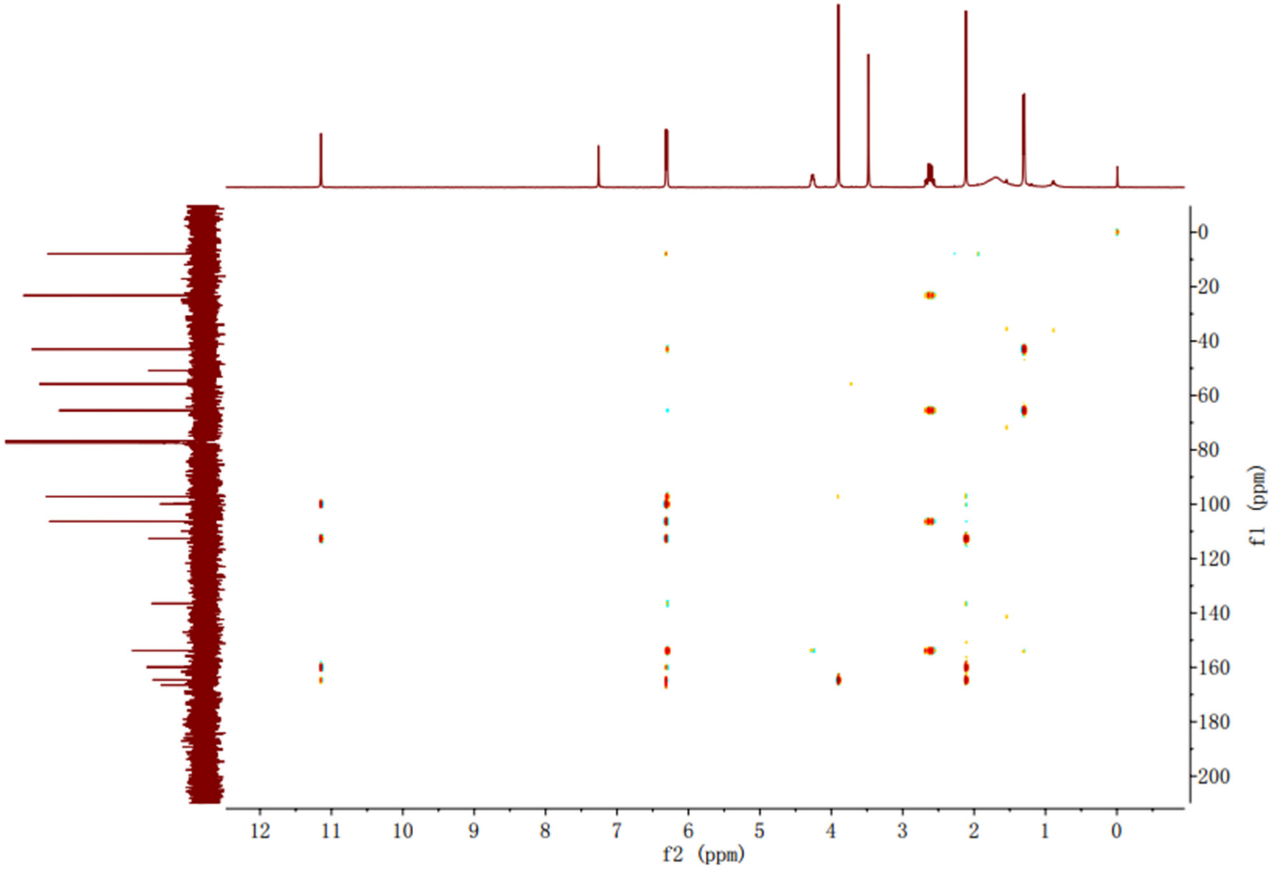

Figure S25. HMBC spectrum of monarubin B (**6**) in $CDCl_3$.

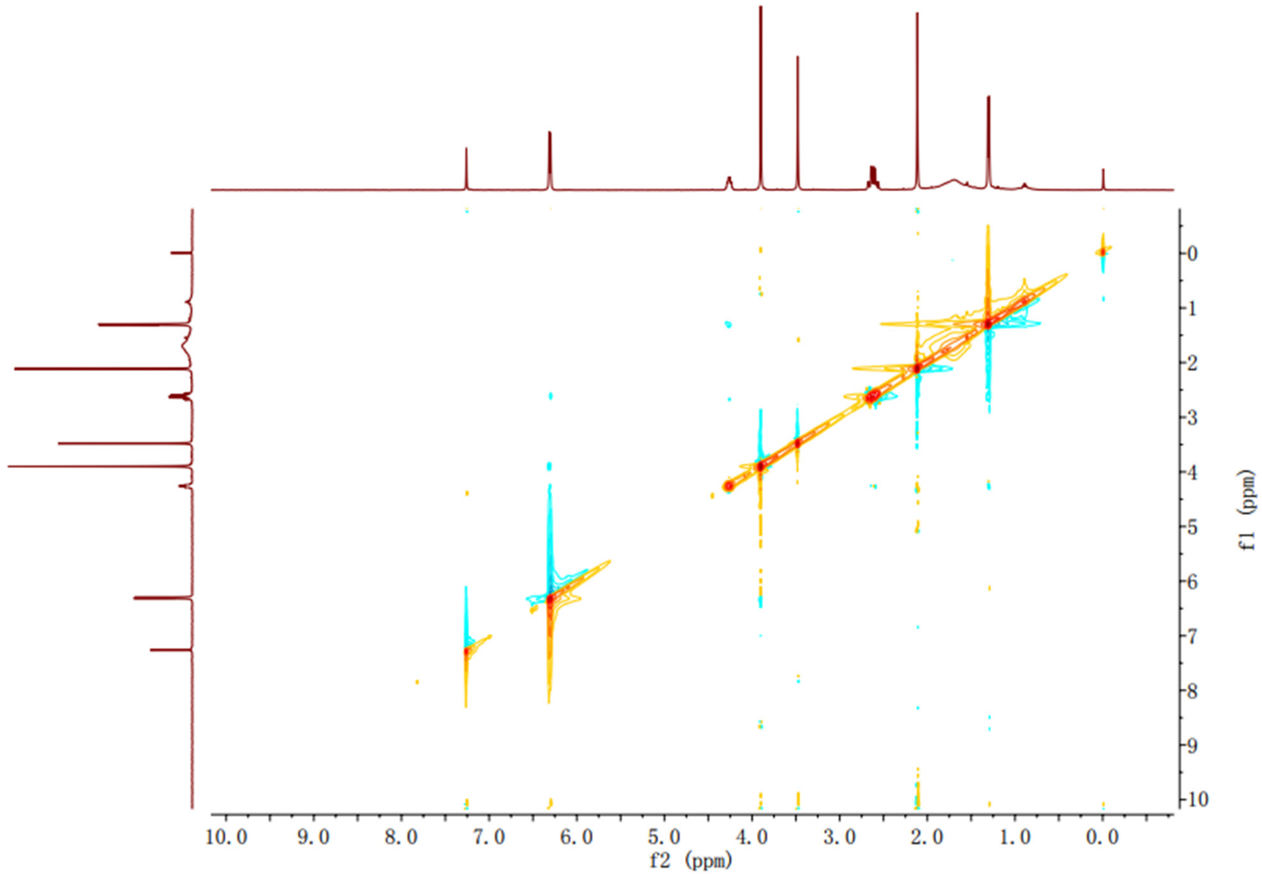

Figure S26. NOESY spectrum of monarubin B (**6**) in $CDCl_3$.

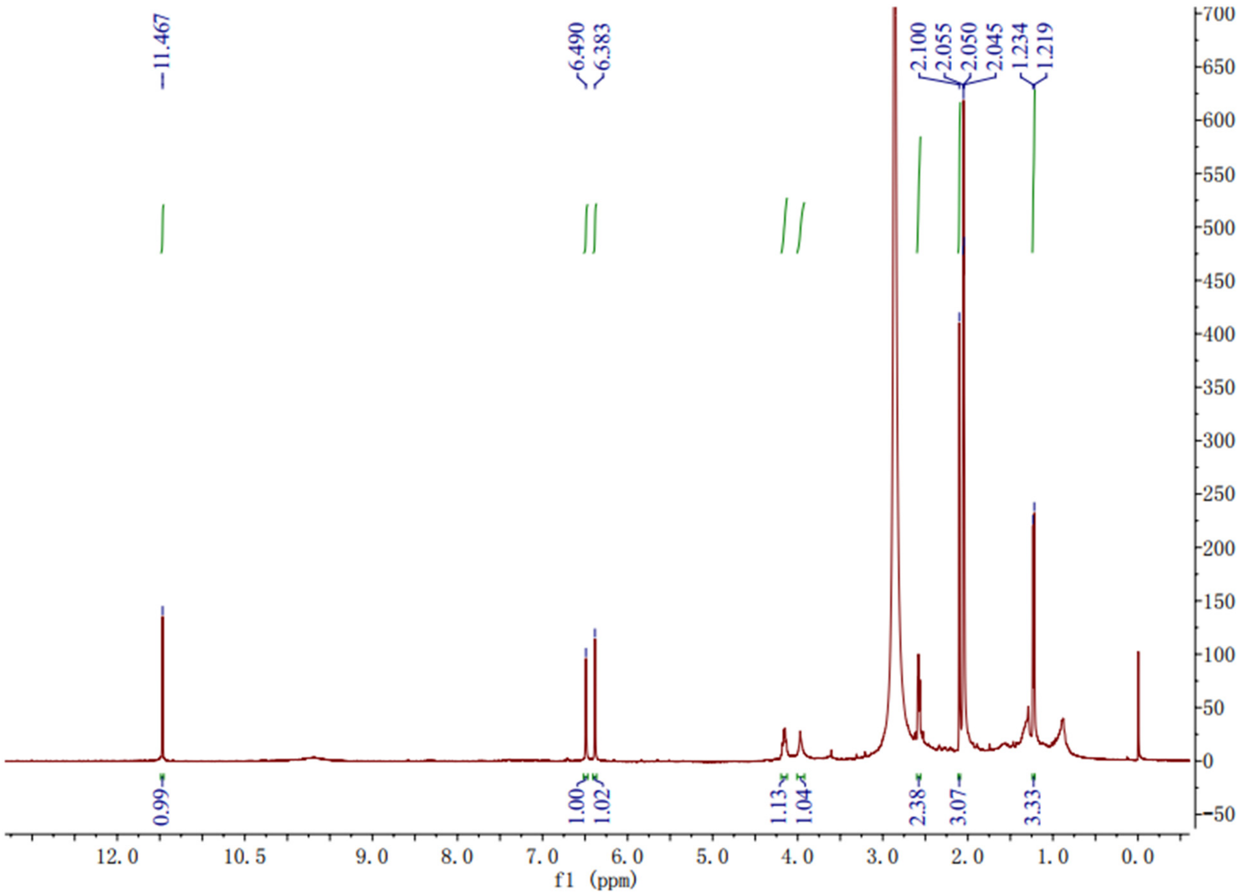

Figure S27. ^{1}H NMR spectrum of lunatinin (**7**) in actone-d_6 (400 MHz).

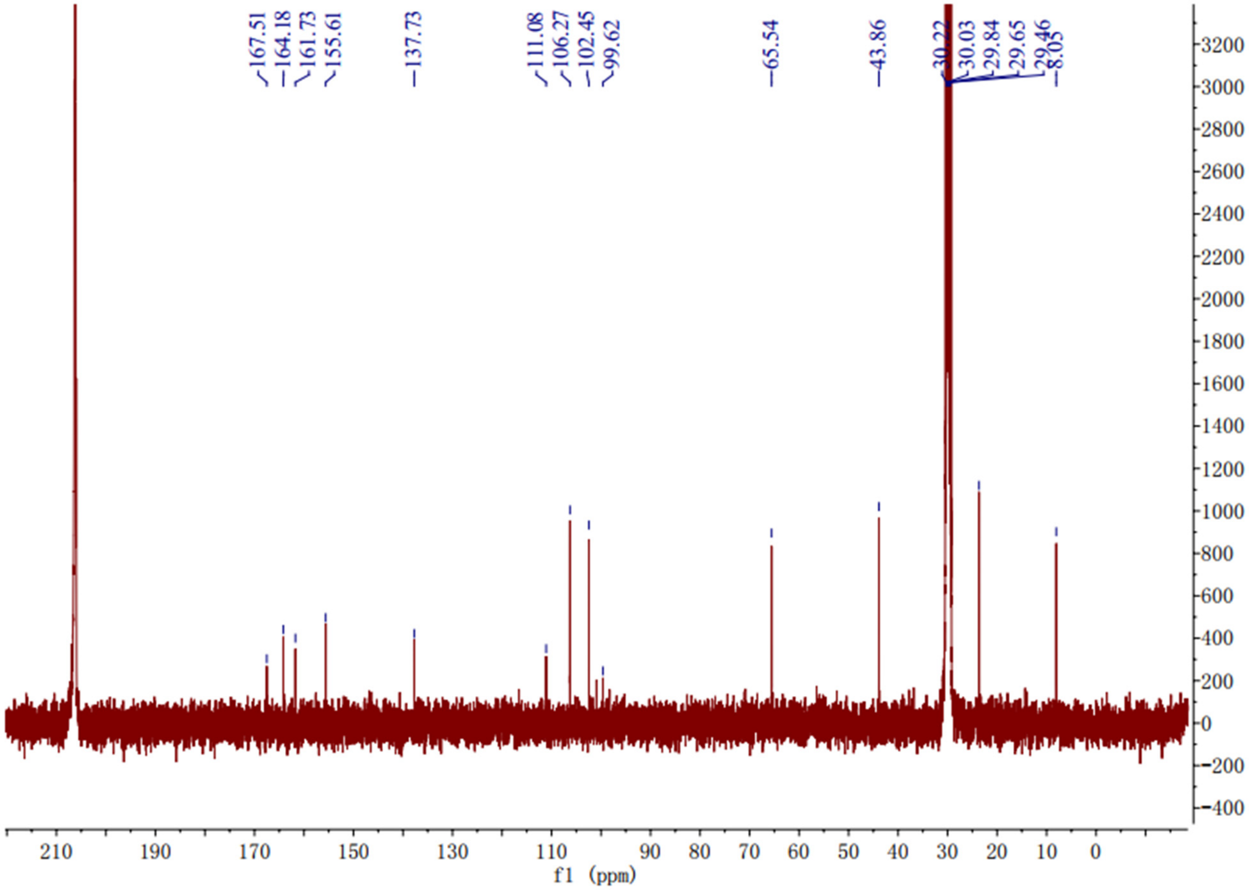

Figure S28. ^{13}C NMR spectrum of lunatinin (**7**) in actone-d_6 (100 MHz).

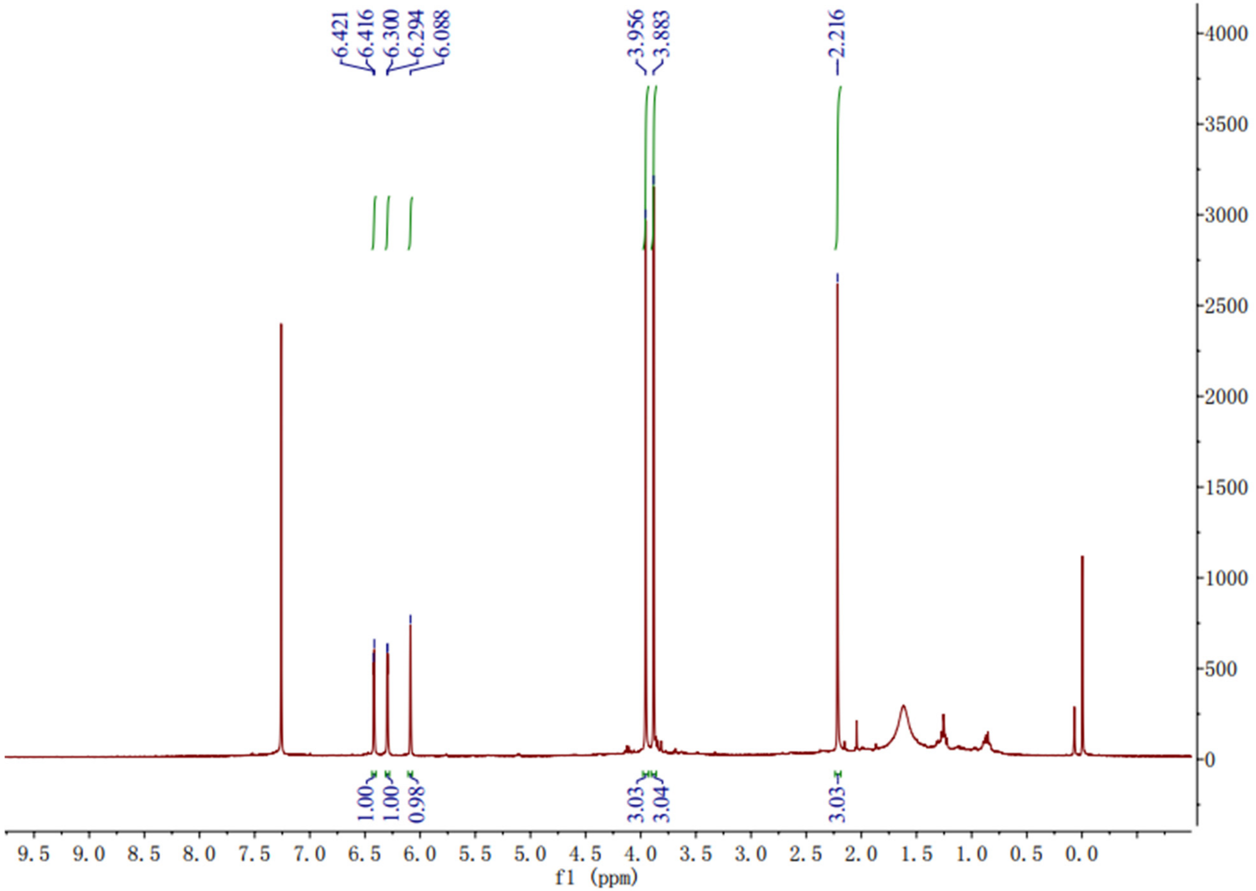

Figure S29. ^{1}H NMR spectrum of 6,8-dimethoxy-3-methylisocoumarin (**8**) in $CDCl_3$ (400 MHz).

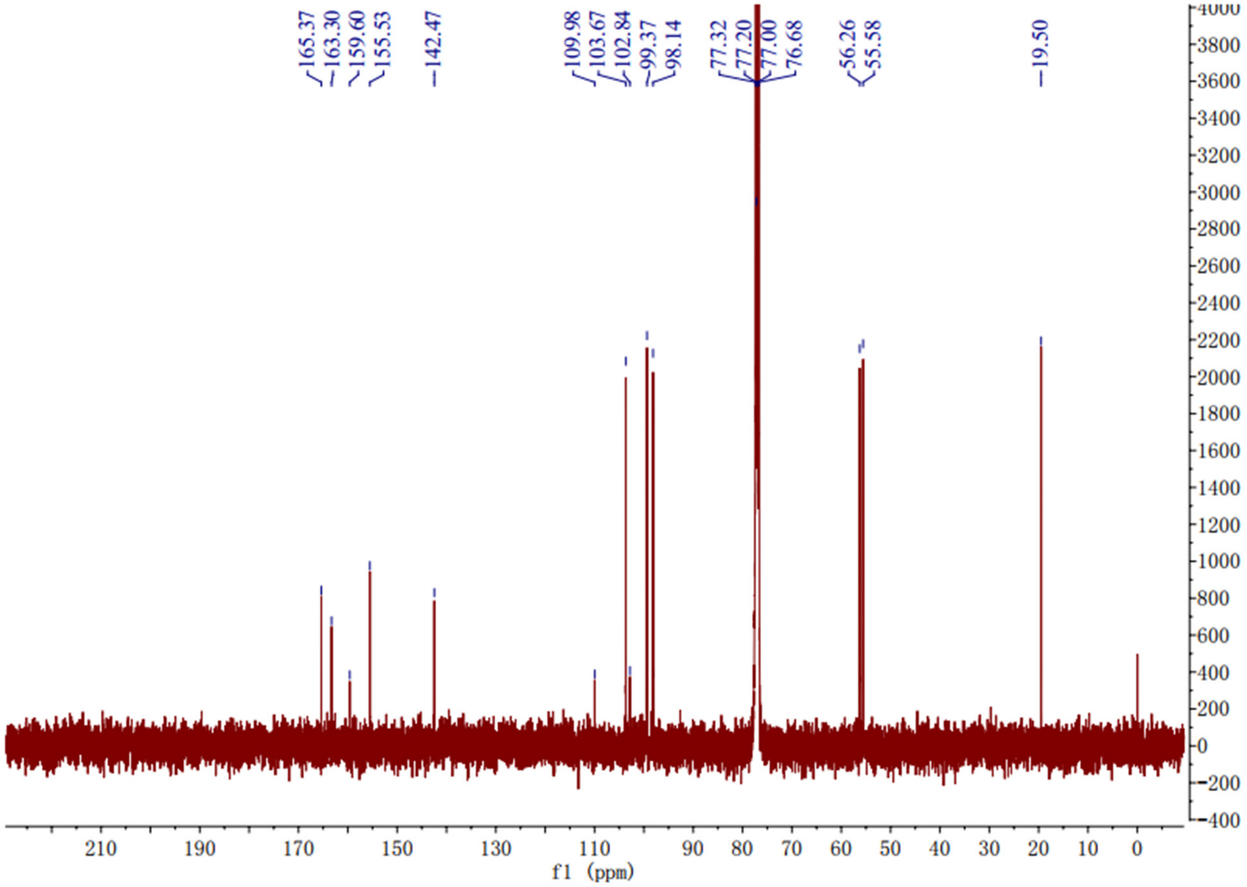

Figure S30. ^{13}C NMR spectrum of 6,8-dimethoxy-3-methylisocoumarin (**8**) in $CDCl_3$ (100 MHz).

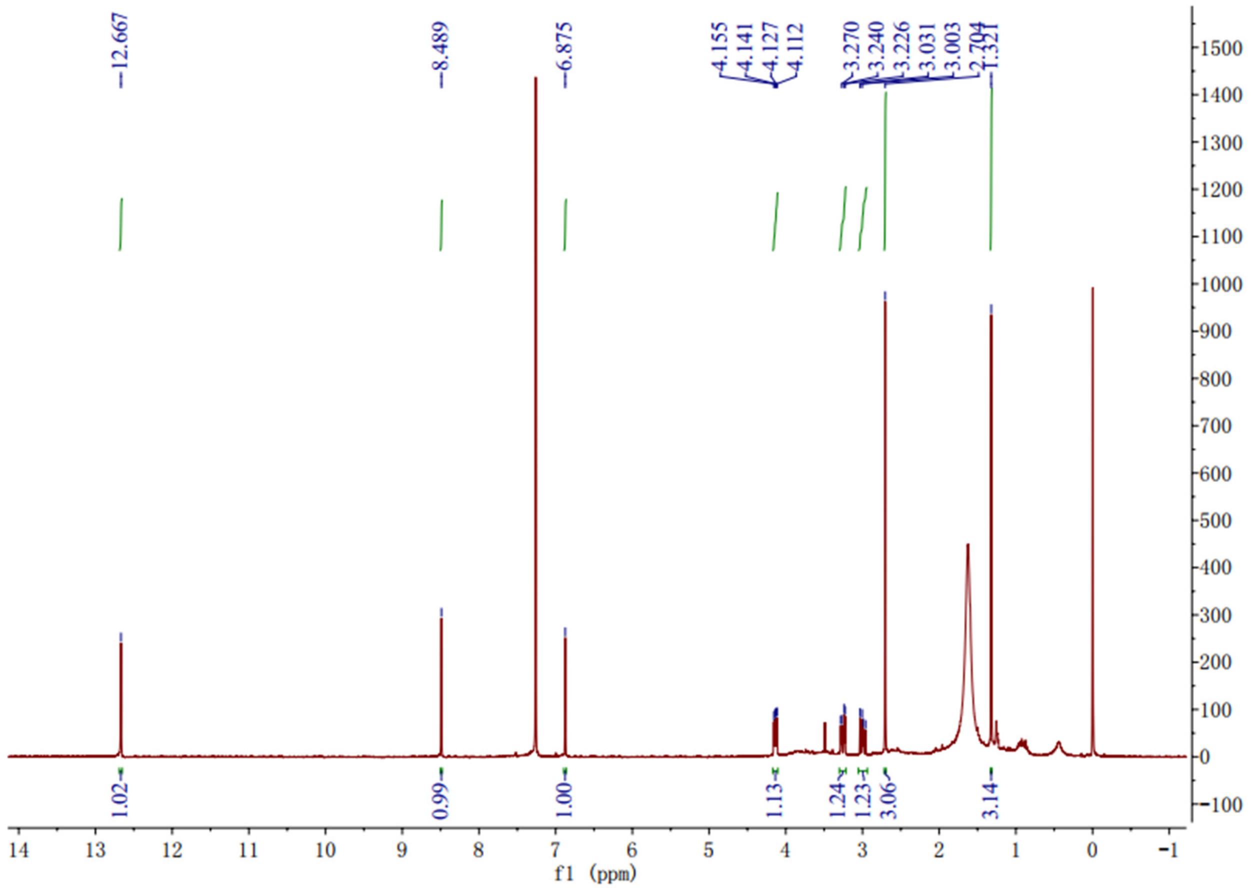

Figure S31. ^{1}H NMR spectrum of monaspurpurone (**9**) in $CDCl_3$ (400 MHz).

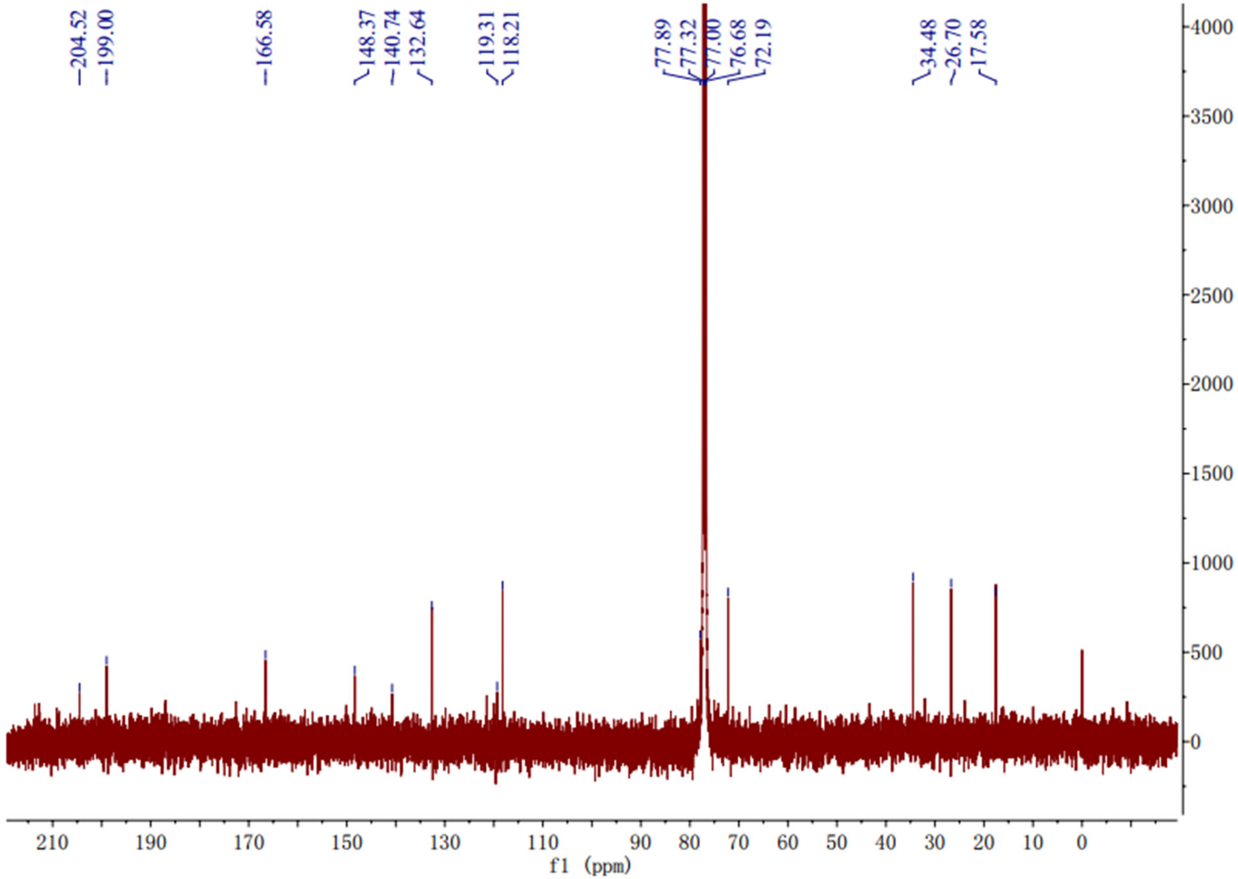

Figure S32. ^{13}C NMR spectrum of monaspurpurone (**9**) in $CDCl_3$ (100 MHz).

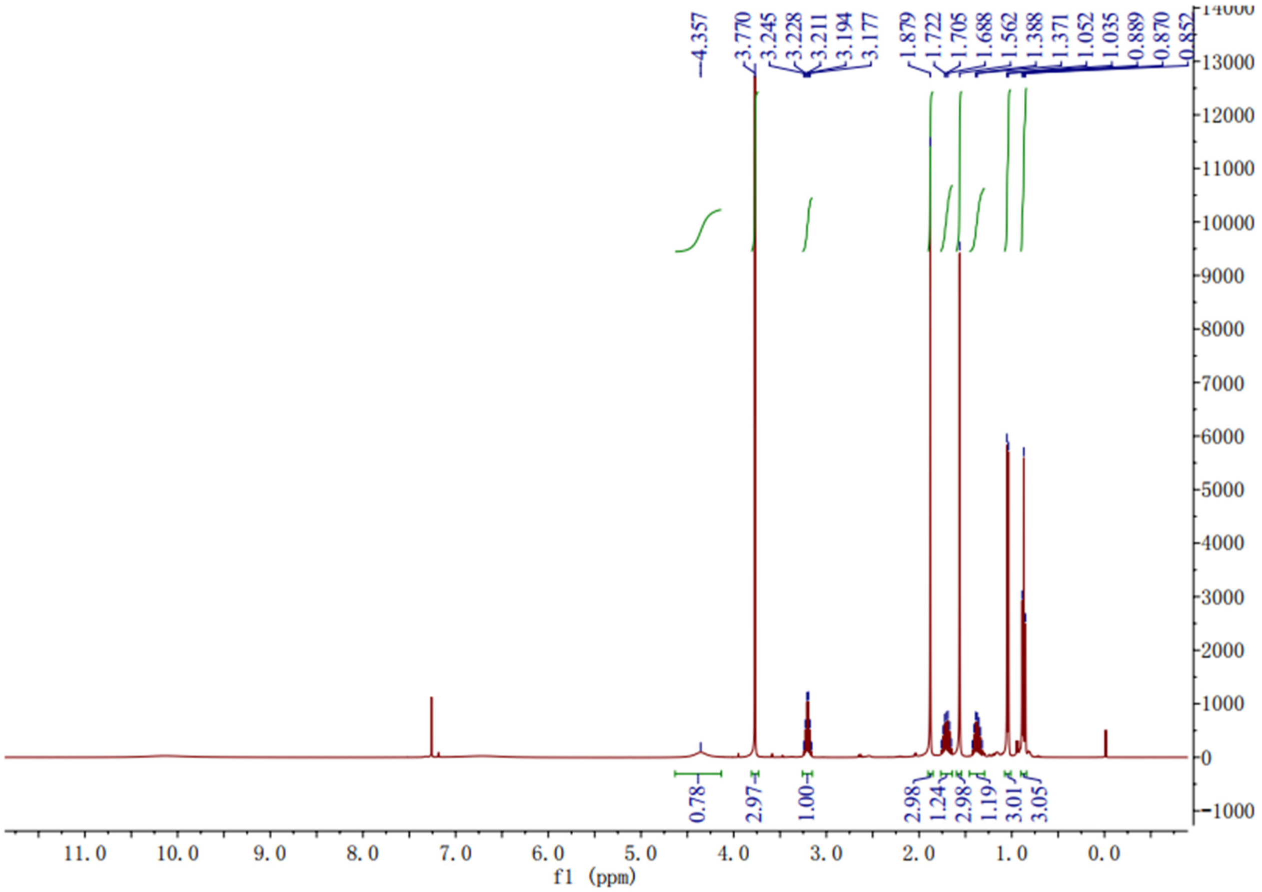

Figure S33. ^{1}H NMR spectrum of 5-amino-2,6-dimethyl-6-hydroxy-4-(2'-methyl-1-oxobutyl)-3-methoxy-2,4-cyclohexadien-1-one (**10**) in $CDCl_3$ (400 MHz).

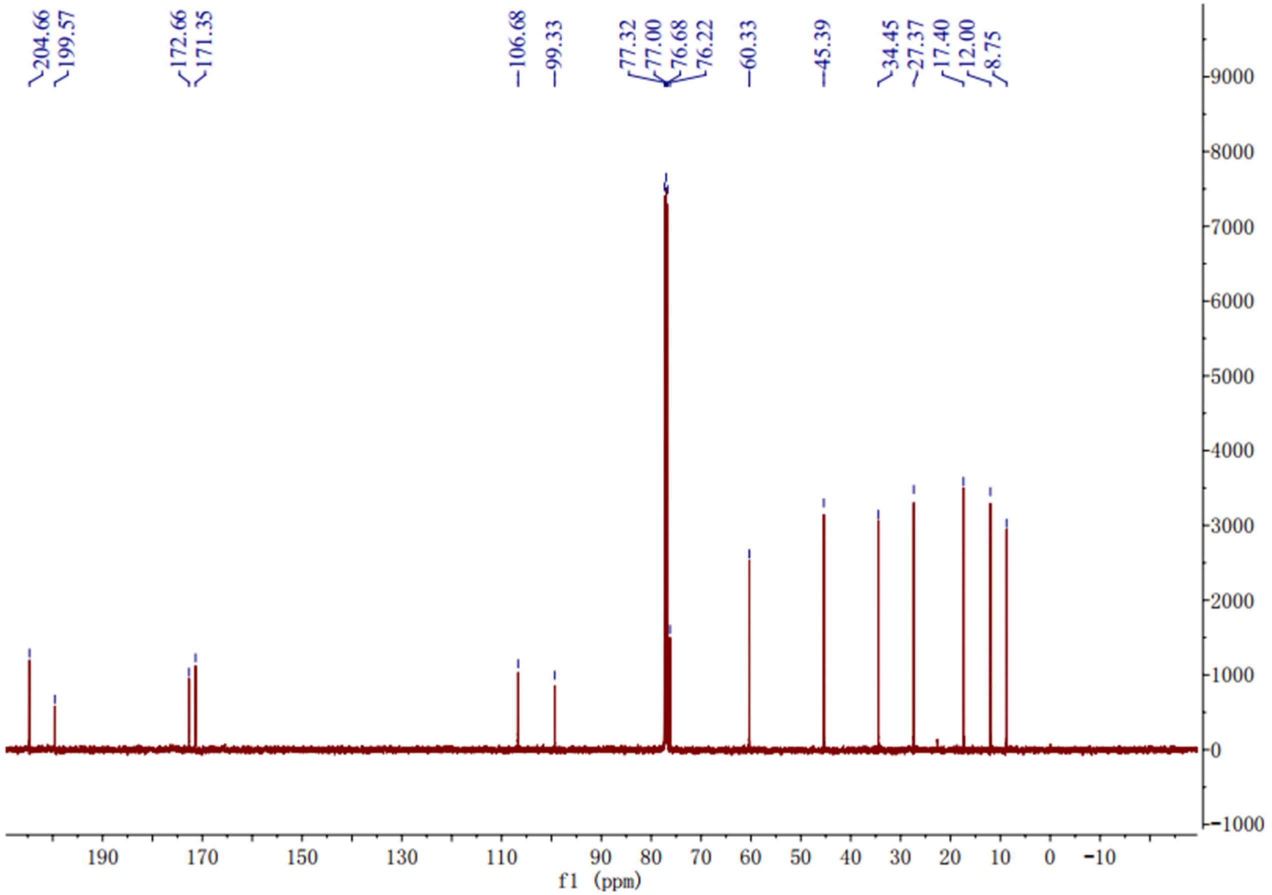

Figure S34. ^{13}C NMR spectrum of 5-amino-2,6-dimethyl-6-hydroxy-4-(2'-methyl-1-oxobutyl)-3-methoxy-2,4-cyclohexadien-1-one (**10**) in $CDCl_3$ (100 MHz).

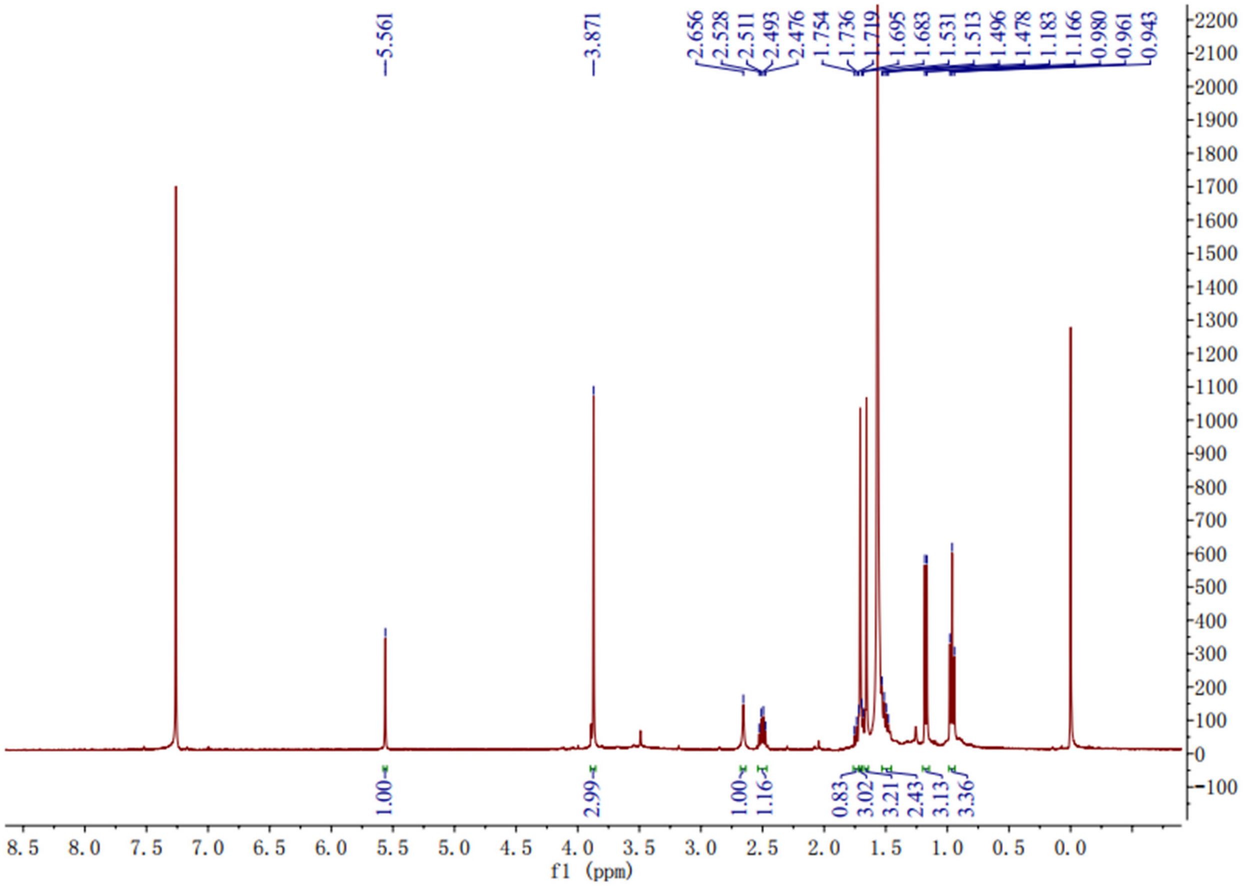

Figure S35. ^{1}H NMR spectrum of phomaligol A (**11**) in $CDCl_3$ (400 MHz).

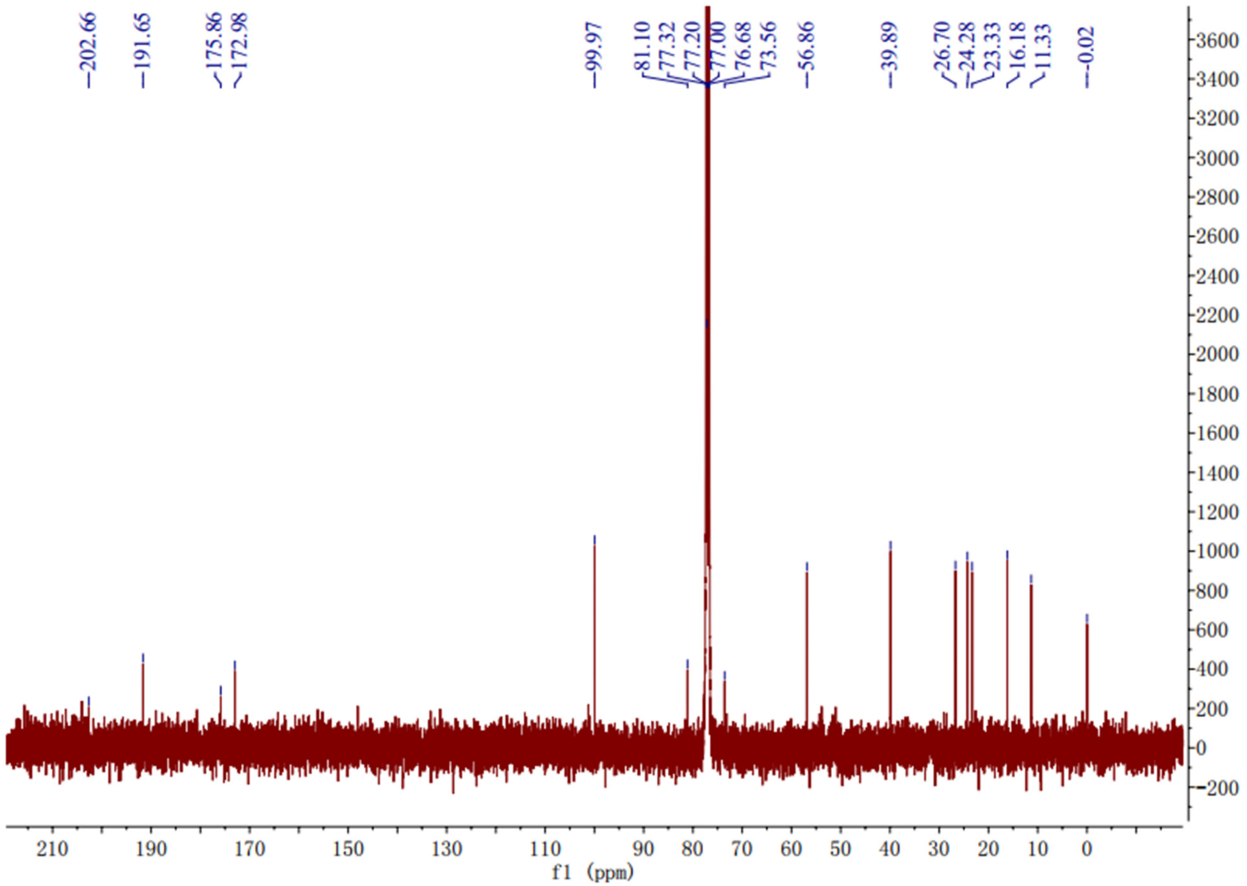

Figure S36. ^{13}C NMR spectrum of phomaligol A (**11**) in $CDCl_3$ (100 MHz).

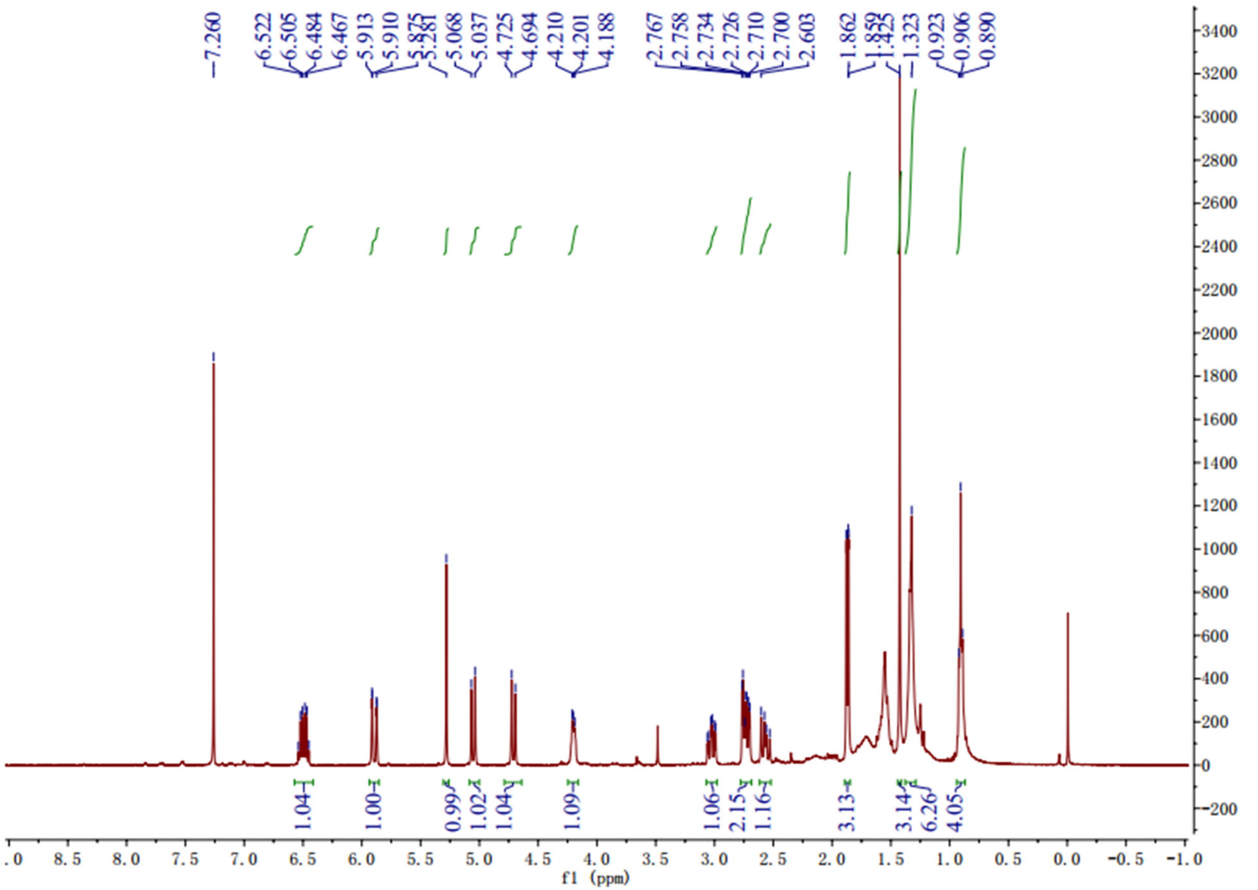

Figure S37. ^{1}H NMR spectrum of monascuspiloin (**12**) in $CDCl_3$ (400 MHz).

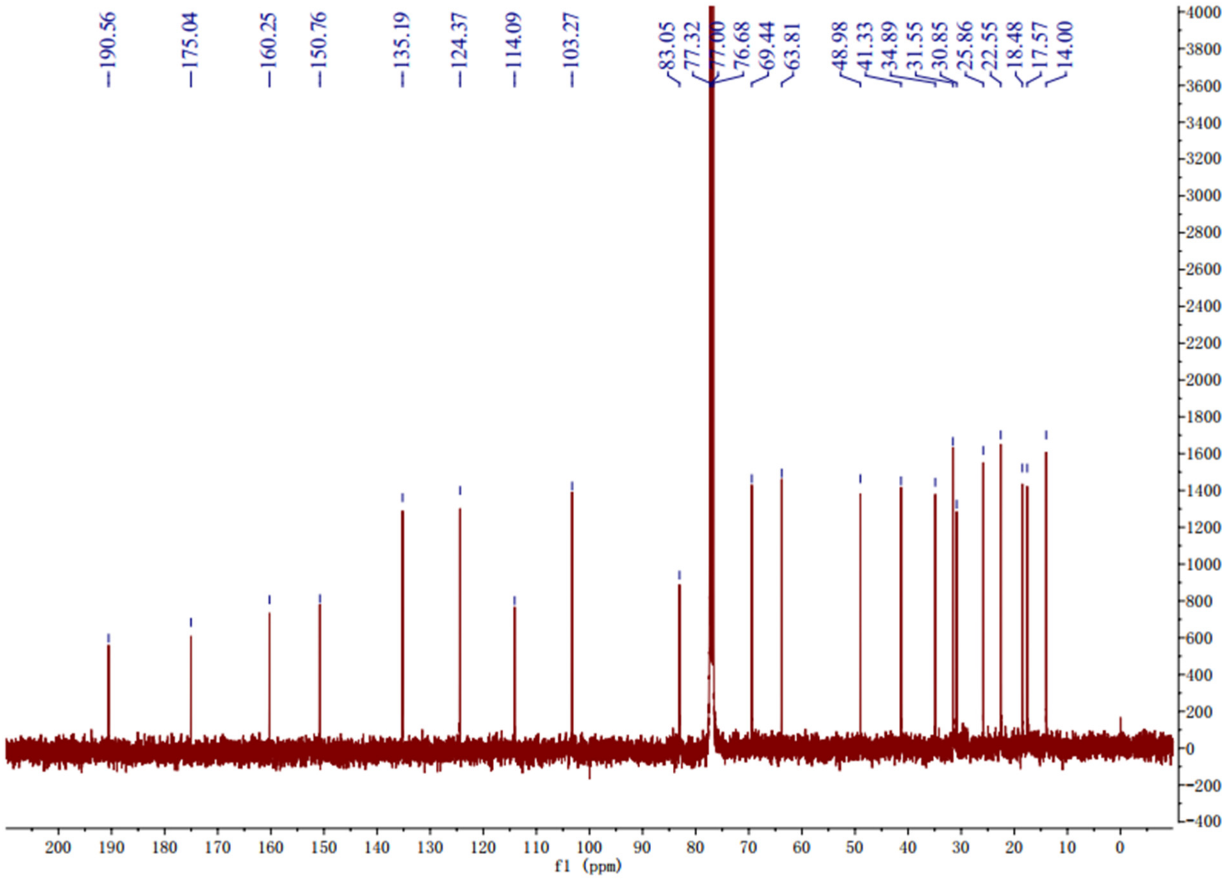

Figure S38. ^{13}C NMR spectrum of monascuspiloin (**12**) in $CDCl_3$ (100 MHz).

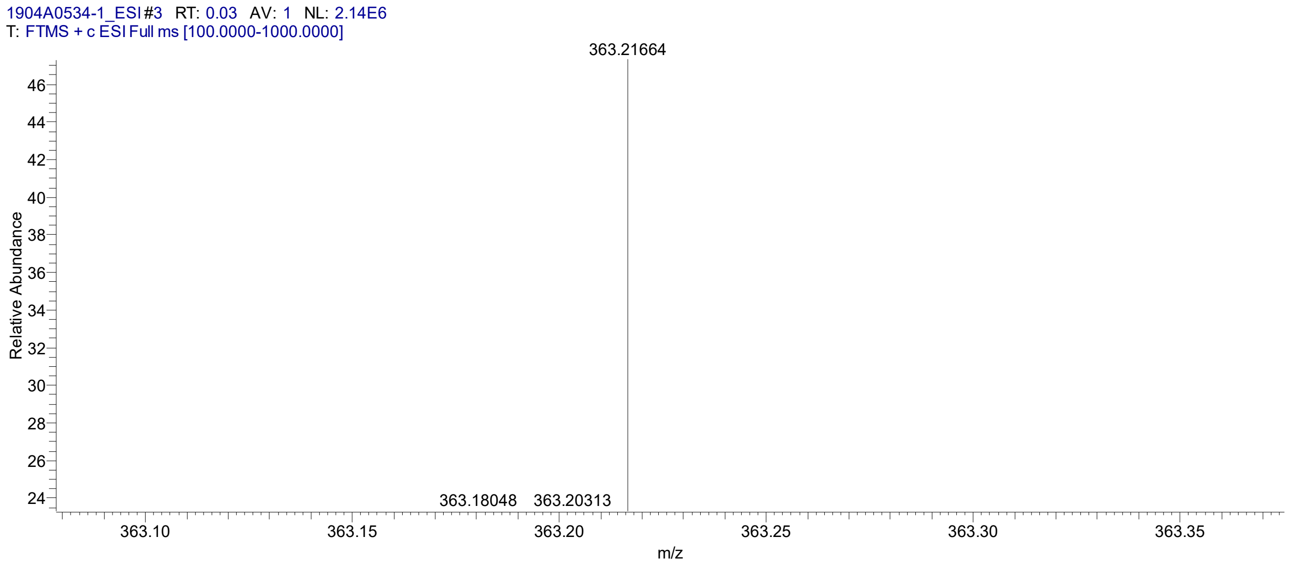

SPECTRUM -simulation :				
m/z	Theo. Mass	Delta (ppm)	RDB equiv.	Composition
363.21664	363.21664	0.11	6.5	C21 H31 O5

Figure S39. HR-(+) ESI-MS spectrum of monarubin C (**13**).

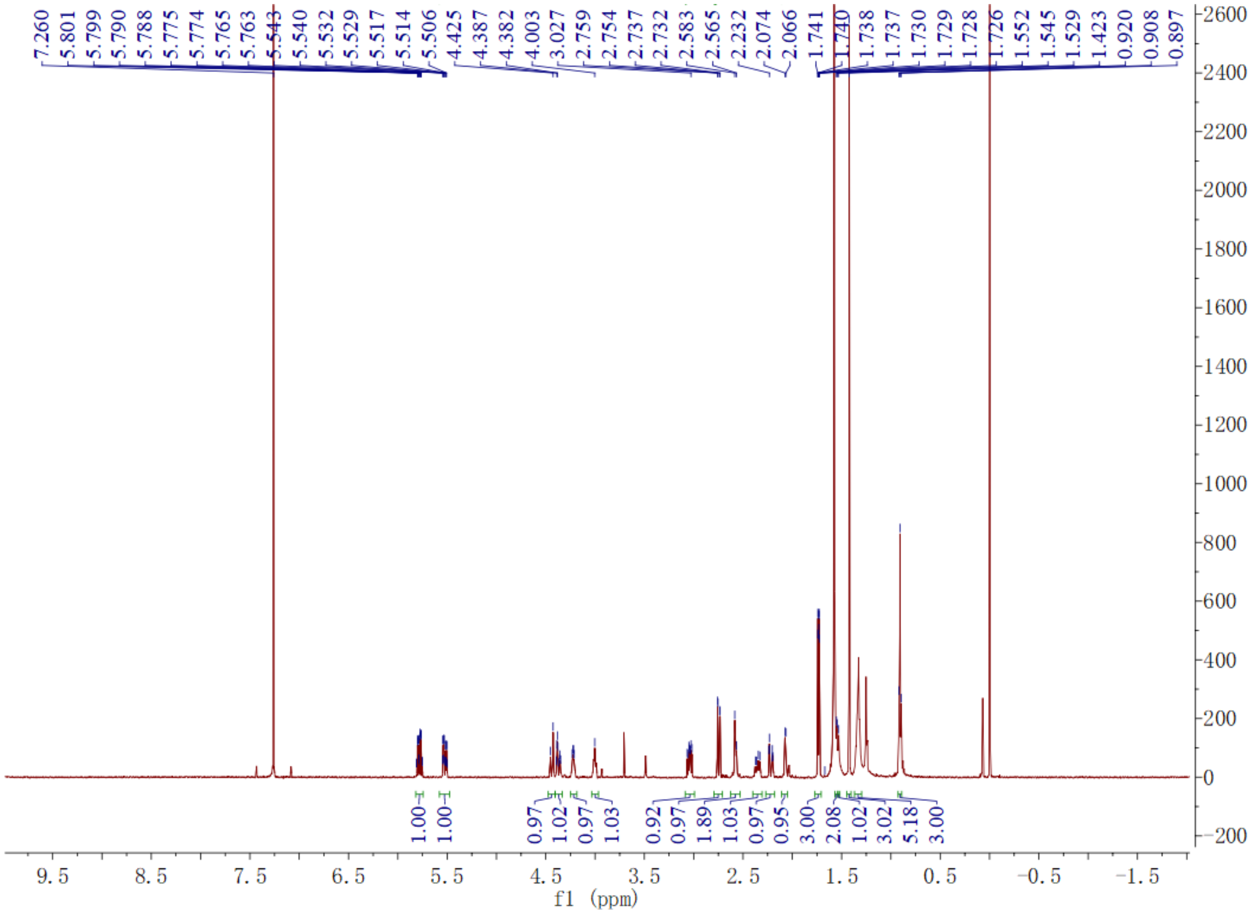

Figure S40. ^{1}H NMR spectrum of monarubin C (**13**) in $CDCl_3$ (600 MHz).

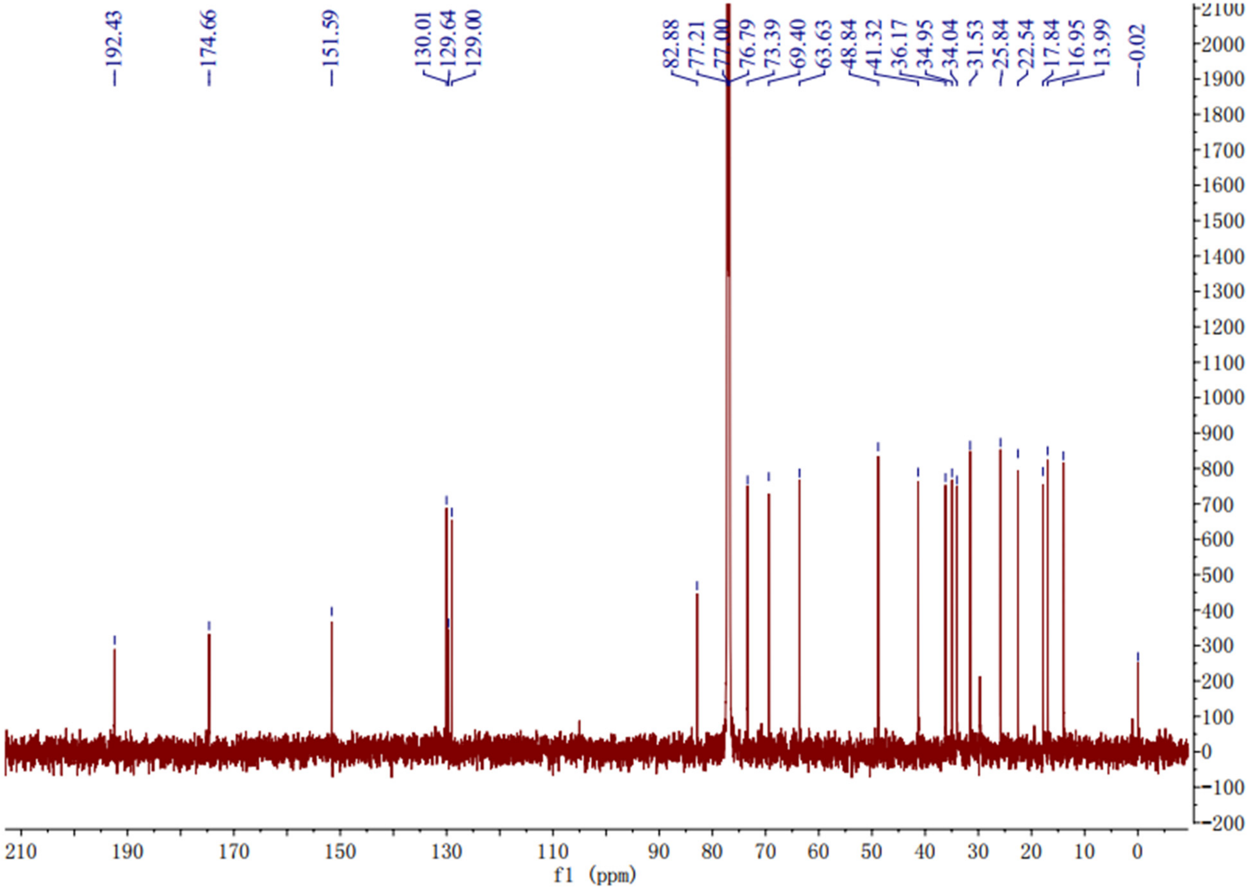

Figure S41. ^{13}C NMR spectrum of monarubin C (**13**) in $CDCl_3$ (150 MHz).

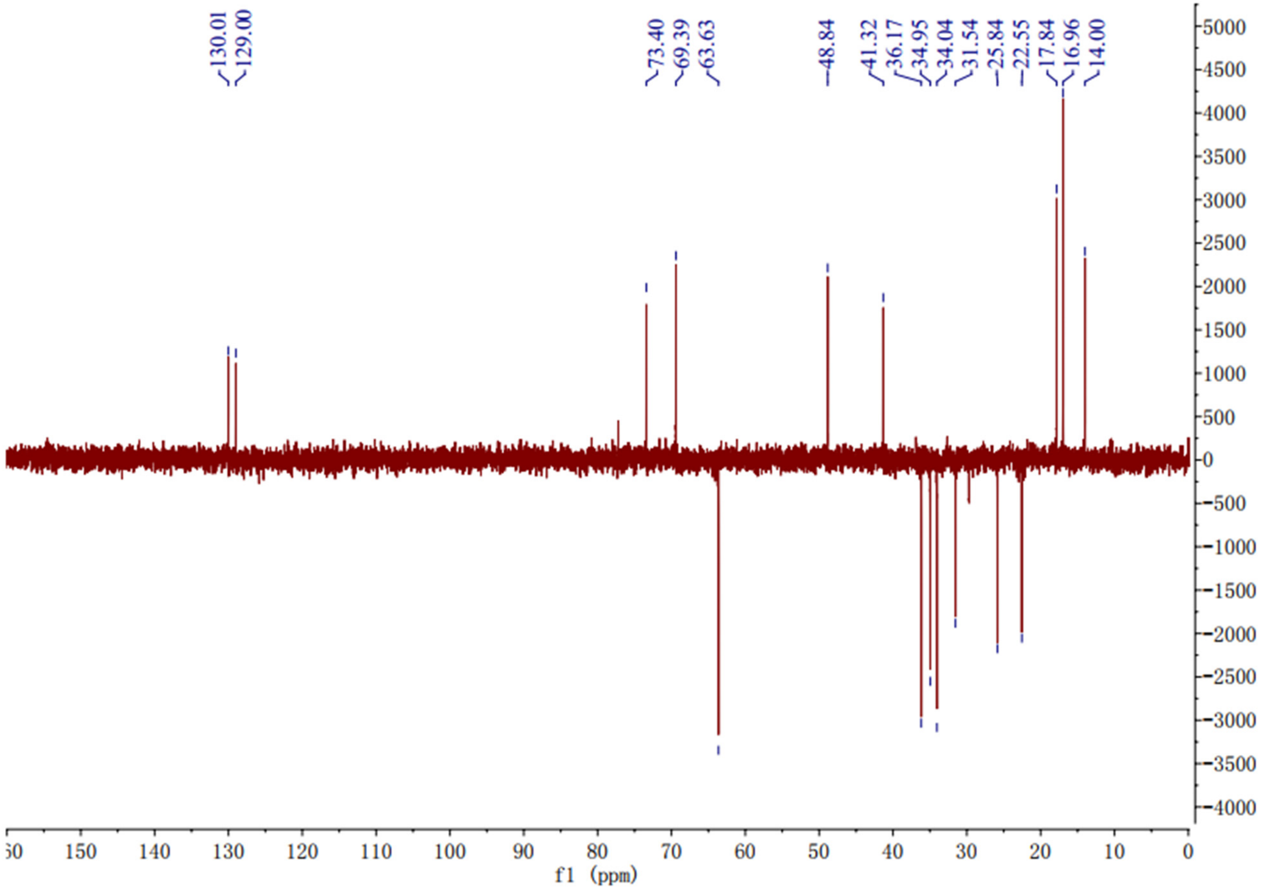

Figure S42. DEPT 135 spectrum of monarubin C (**13**) in $CDCl_3$ (150 MHz).

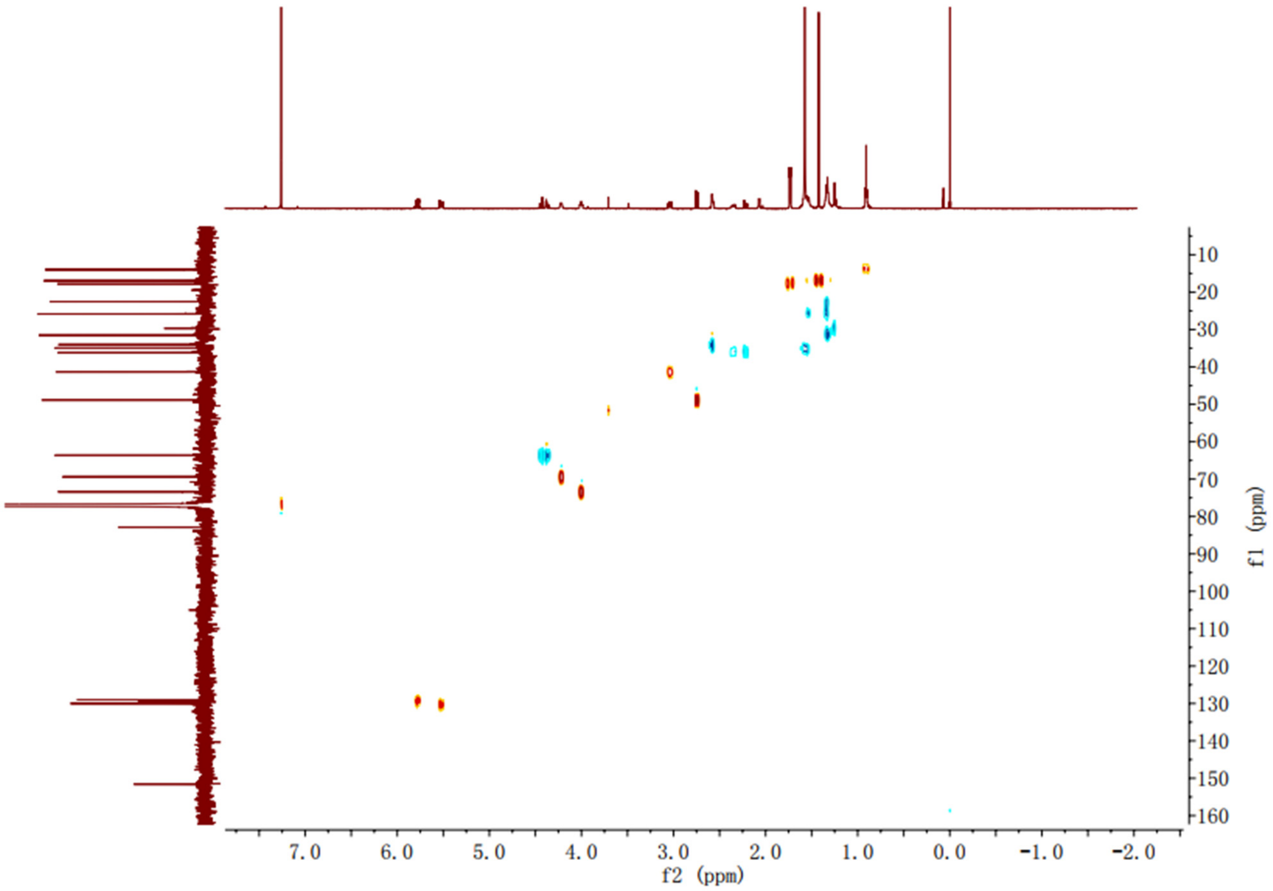

Figure S43. HMQC spectrum of monarubin C (**13**) in $CDCl_3$.

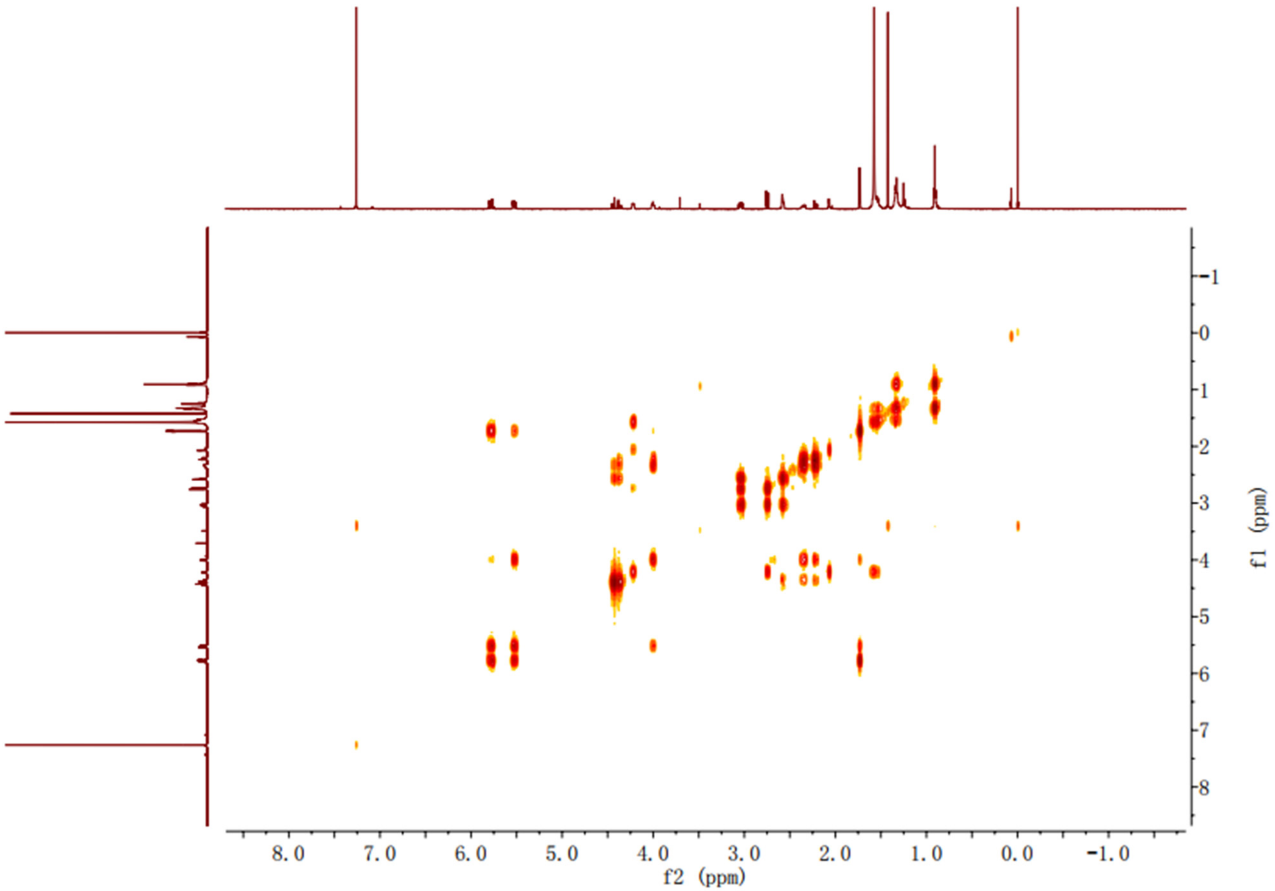

Figure S44. ^{1}H–^{1}HCOSY spectrum of monarubin C (**13**) in $CDCl_3$.

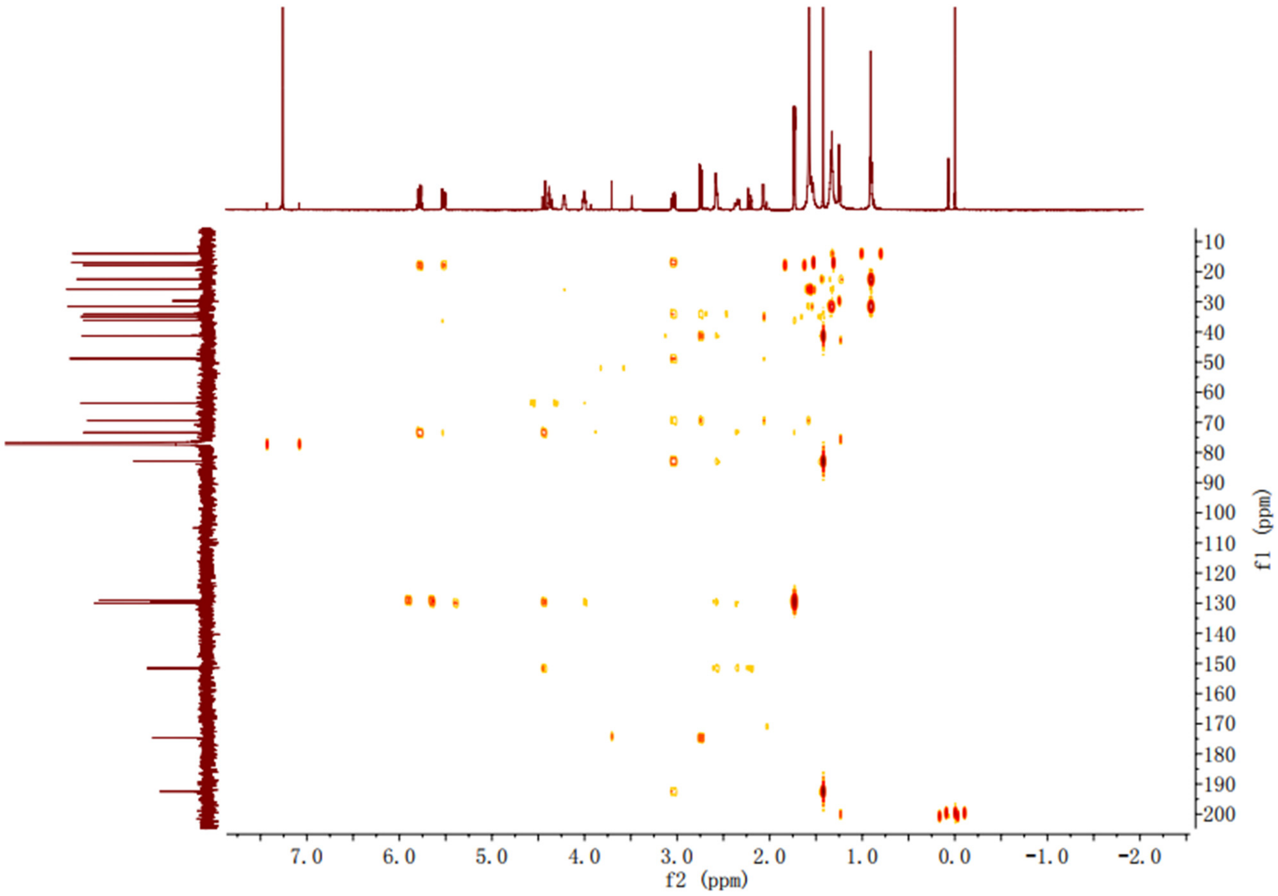

Figure S45. HMBC spectrum of monarubin C (**13**) in $CDCl_3$.

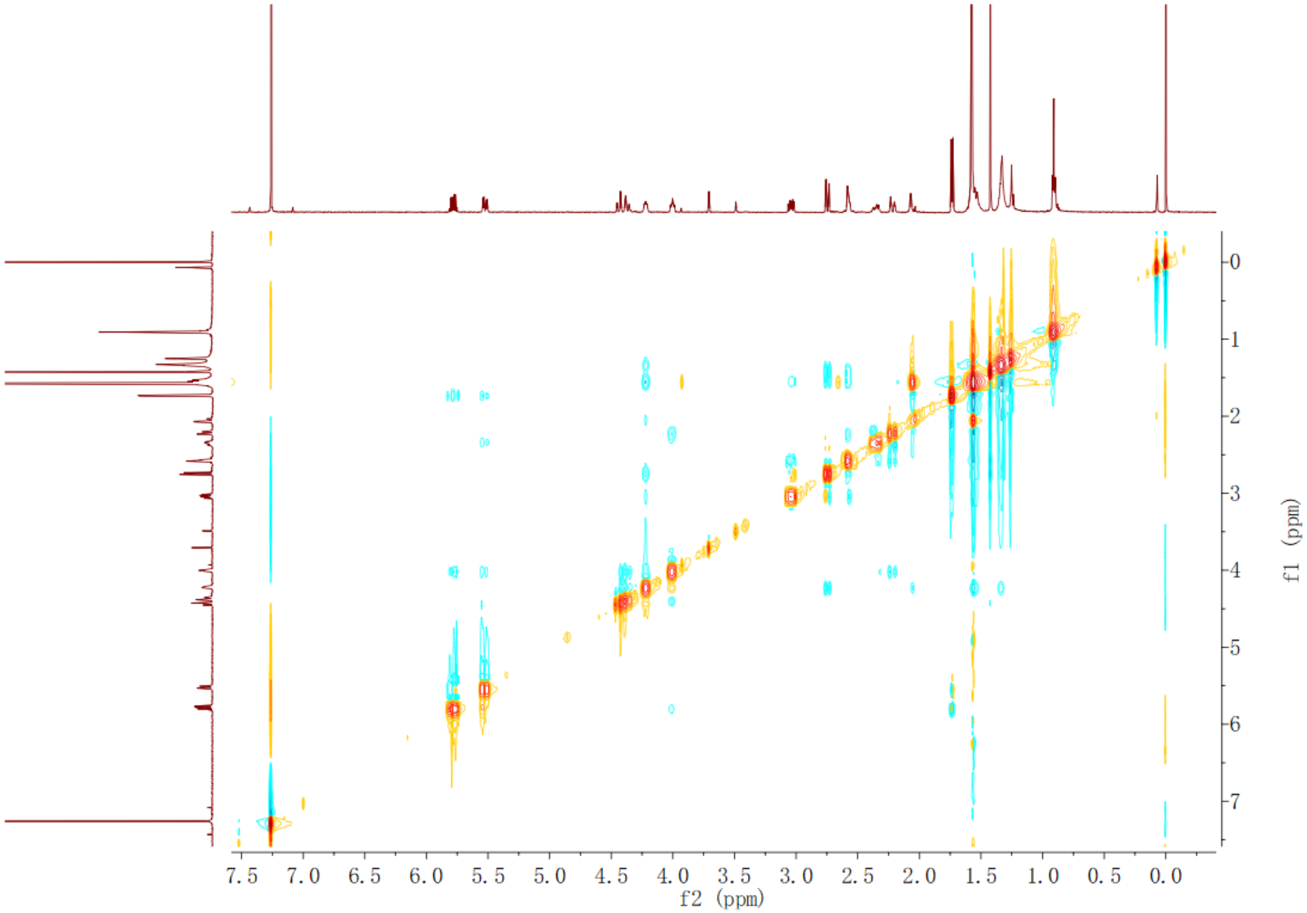

Figure S46. NOESY spectrum of monarubin C (**13**) in $CDCl_3$.

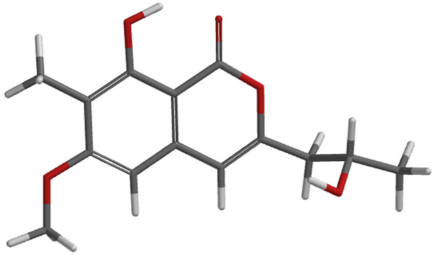

1 (18.6%)

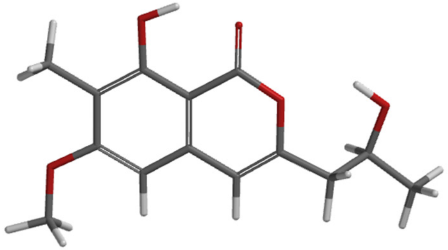

2 (13.7%)

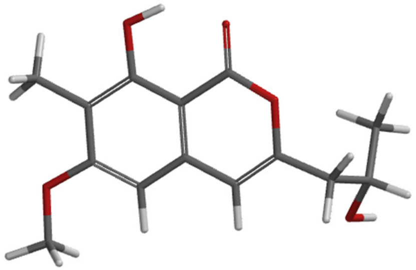

3 (9.3%)

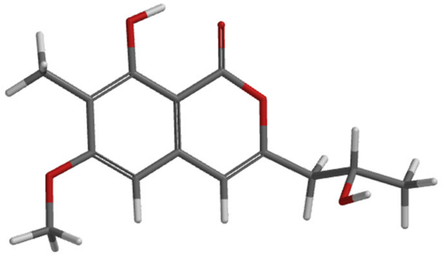

4 (7.9%)

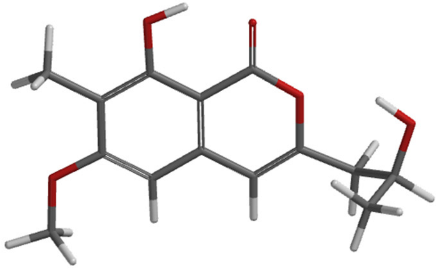

5 (5.9%)

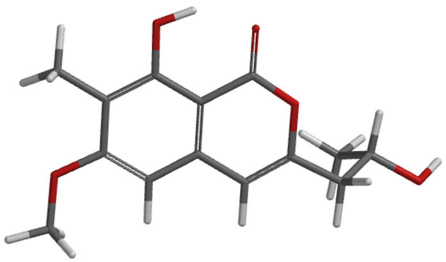

6 (5.8%)

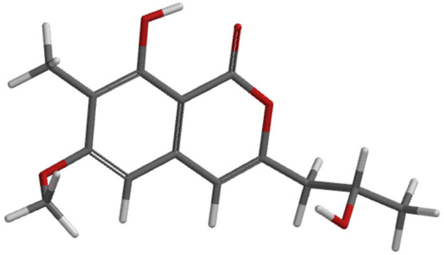

7 (4.9%)

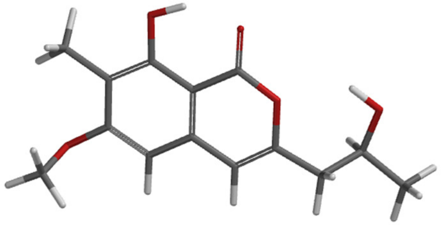

8 (4.0%)

Figure S47. The most stable conformers of 11*S*-**6** calculated at the B3LYP/6-31+G(d) level. Relative populations are in parentheses.

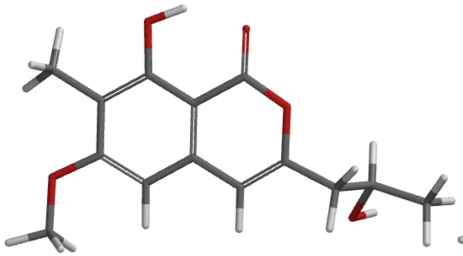

1 (17.5%)

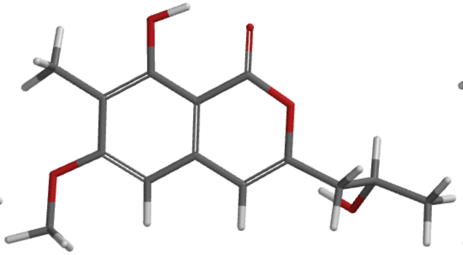

2 (13.0%)

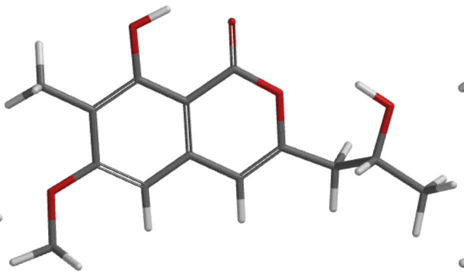

3 (8.8%)

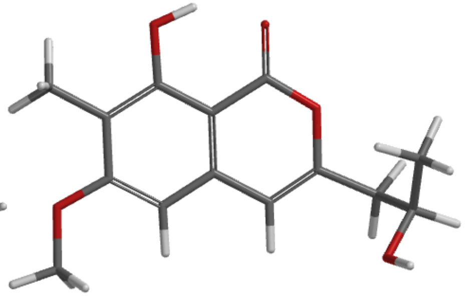

4 (7.6%)

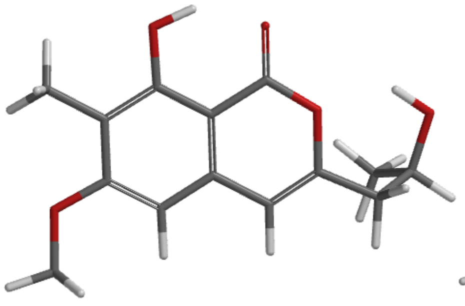

5 (5.6%)

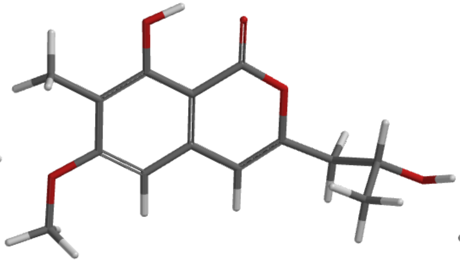

6 (5.5%)

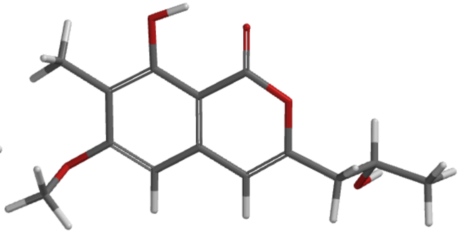

7 (5.4%)

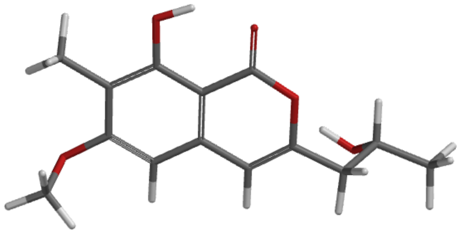

8 (4.8%)

Figure S48. The most stable conformers of 11*R*-**6** calculated at the B3LYP/6-31+G(d) level. Relative populations are in parentheses.

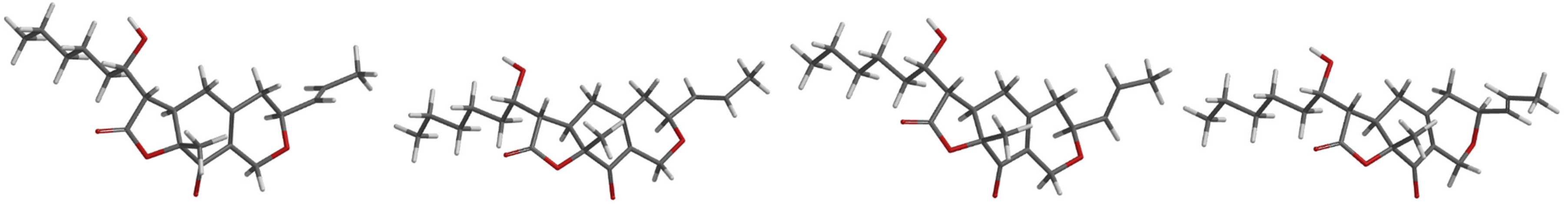

1 (26.4%) 2 (18.6%) 3 (12.3%) 4 (8.3%)

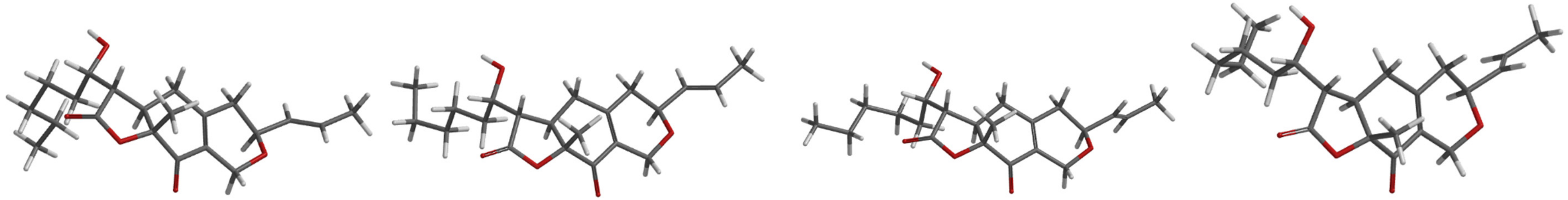

5 (5.1%) 6 (4.6%) 7 (3.4%) 8 (3.2%)

Figure S49. The most stable conformers of 3*S*,6*R*,7*S*,11*S*,17*R*-**13** calculated at the B3LYP/6-31+G(d) level. Relative populations are in parentheses.

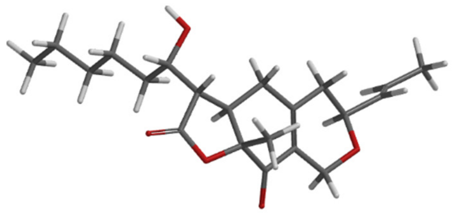

1 (31.3%)

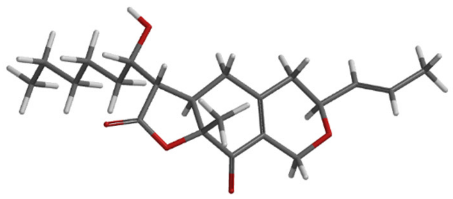

2 (22%)

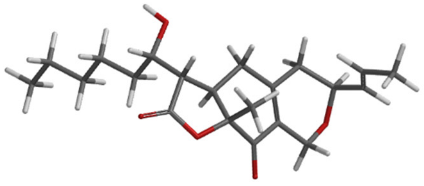

3 (9.8%)

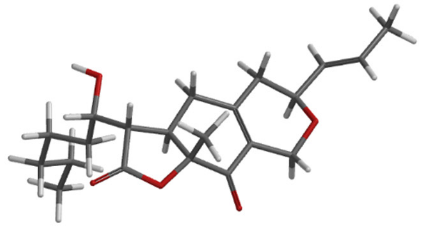

4 (6.0%)

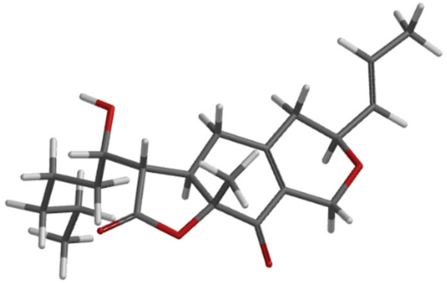

5 (4.0%)

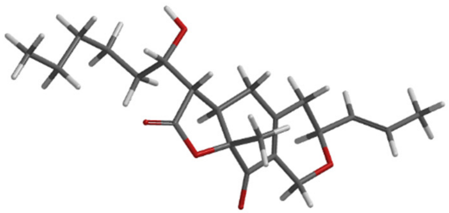

6 (3.0%)

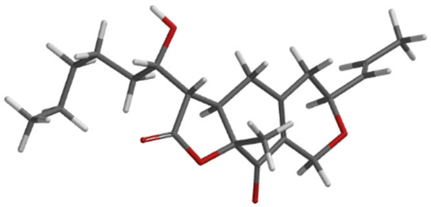

7 (2.9%)

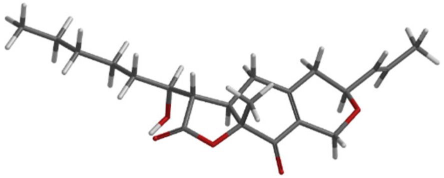

8 (2.8%)

Figure S50. The most stable conformers of 3*S*,6*R*,7*S*,11*S*,17*S*-**13** calculated at the B3LYP/6-31+G(d) level. Relative populations are in parentheses.

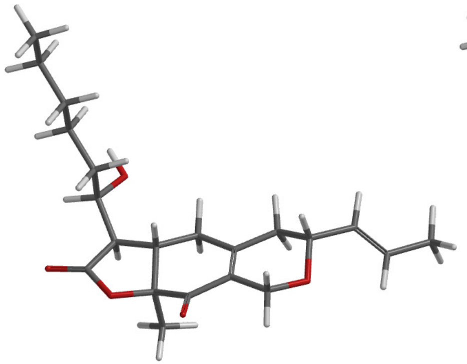

1 (26.8%)

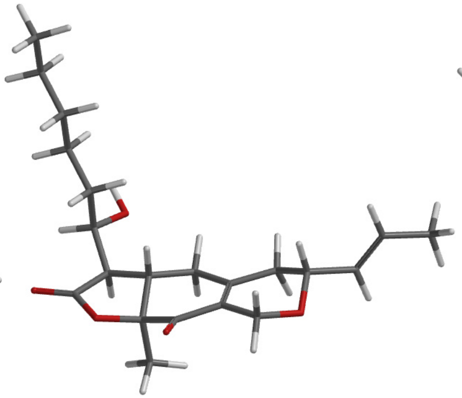

2 (18.8%)

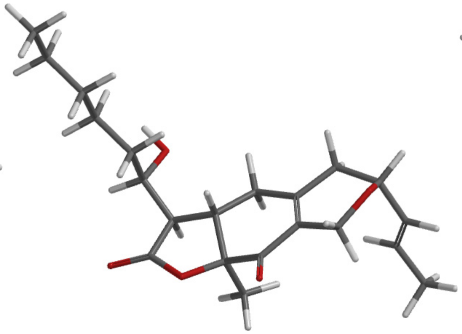

3 (8.4%)

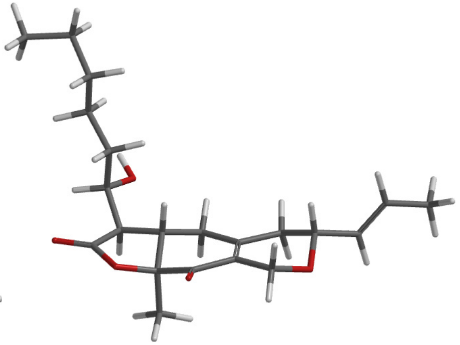

4 (6.8%)

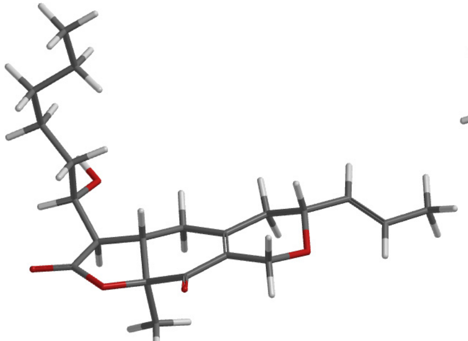

5 (5.1%)

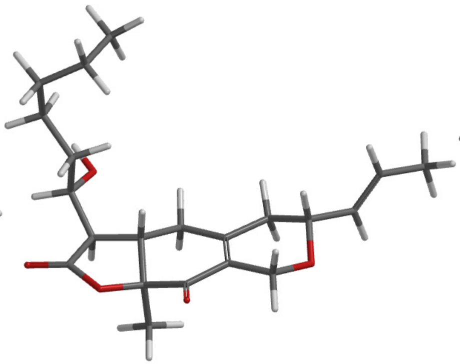

6 (4.7%)

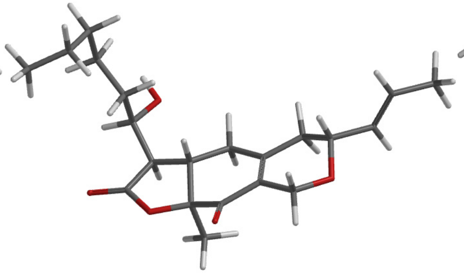

7 (3.3%)

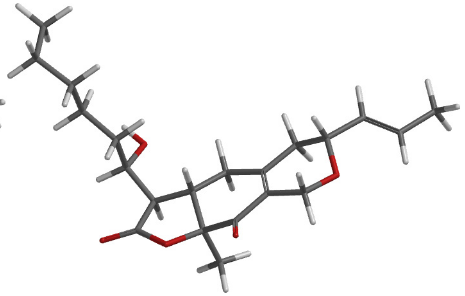

8 (2.6%)

Figure S51. The most stable conformers of 3*R*,6*S*,7*R*,11*R*,17*S*-**13** calculated at the B3LYP/6-31+G(d) level. Relative populations are in parentheses.

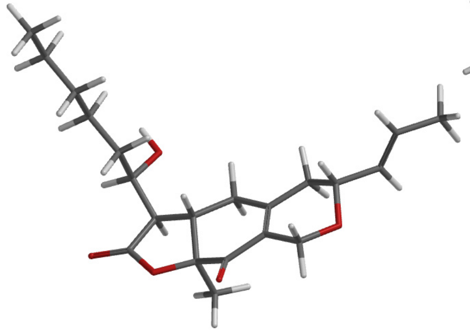

1 (25%)

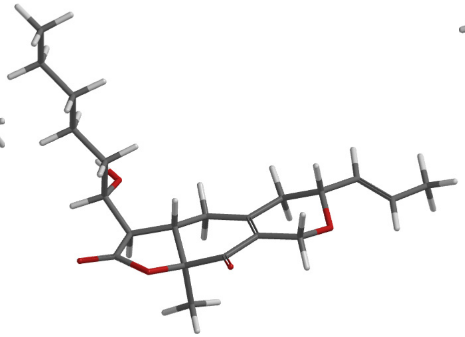

2 (17.5%)

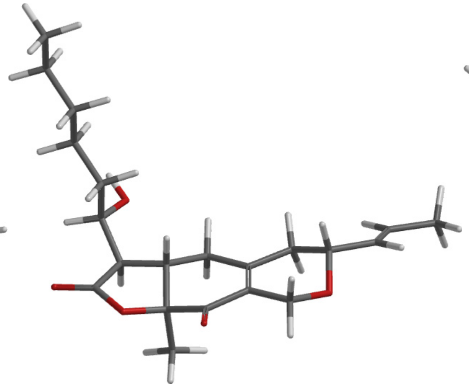

3 (11.7%)

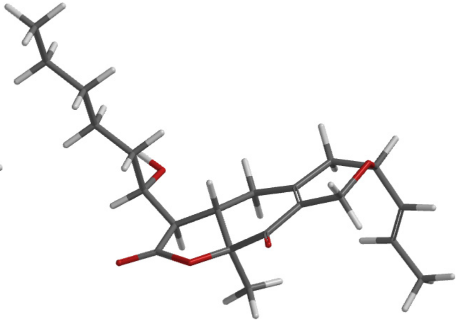

4 (7.8%)

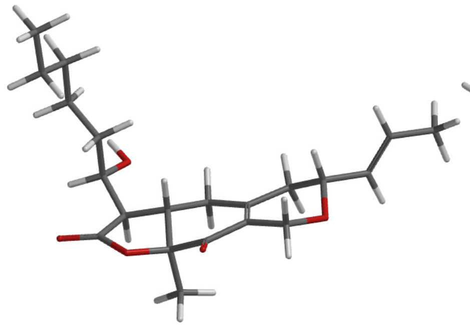

5 (5.4%)

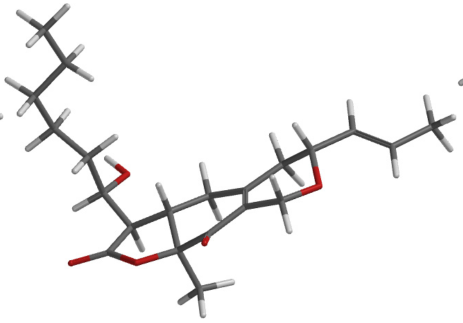

6 (4.8%)

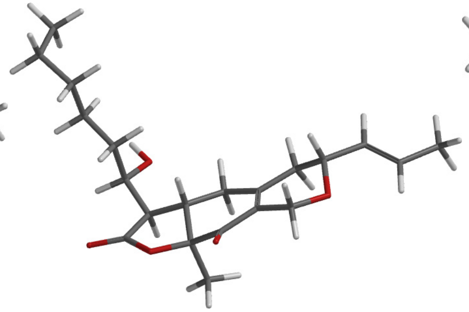

7 (4.4%)

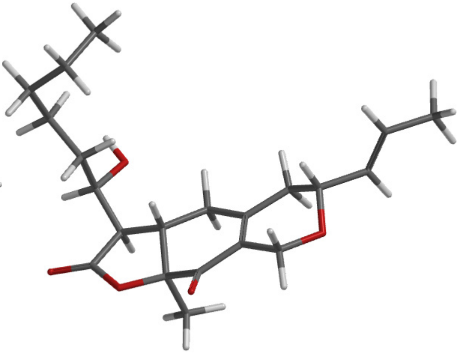

8 (3.1%)

Figure S52. The most stable conformers of 3*R*,6*S*,7*R*,11*R*,17*R*-**13** calculated at the B3LYP/6-31+G(d) level. Relative populations are in parentheses.

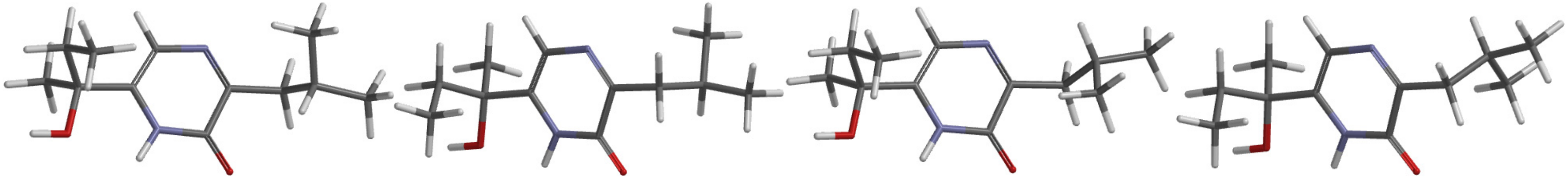

1 (13.2%) 2 (12.6%) 3 (6.7%) 4 (6.4%)

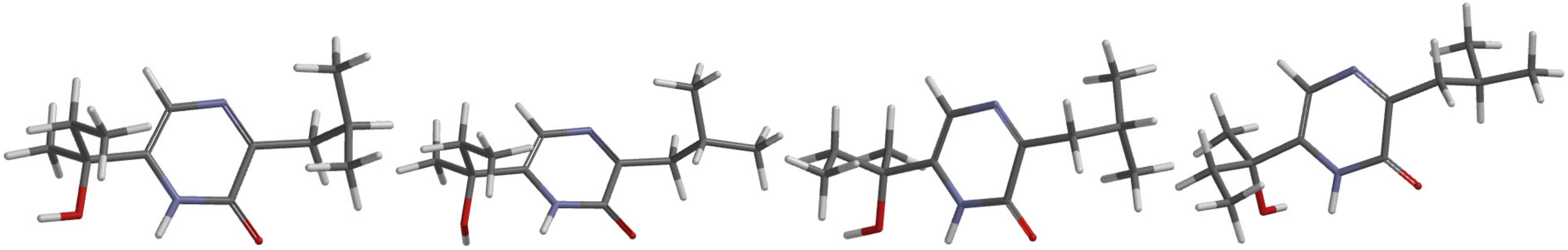

5 (4.1%) 6 (4.0%) 7 (3.8%) 8 (3.8%)

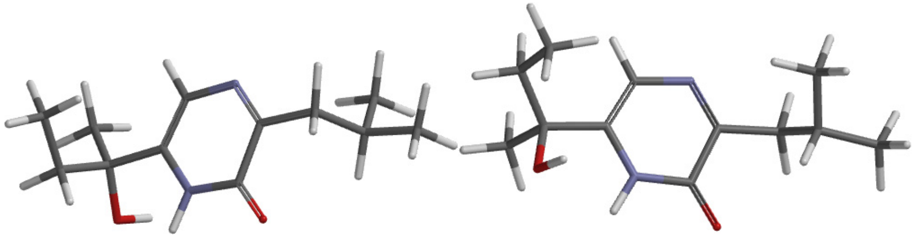

9 (3.7%) 10 (2.4%)

Figure S53. The most stable conformers of 11*S*-**3** calculated at the B3LYP/6-31+G(d) level. Relative populations are in parentheses.

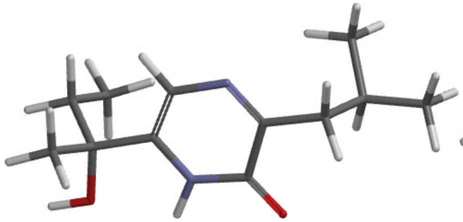

1 (12.2%)

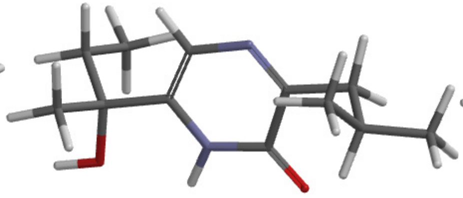

2 (11.7%)

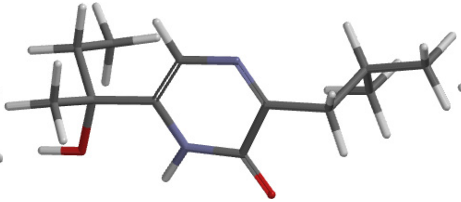

3 (6.2%)

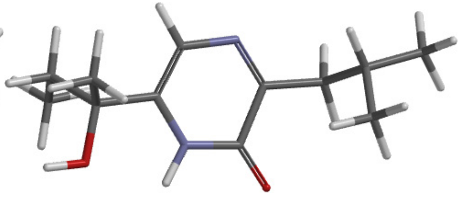

4 (5.9%)

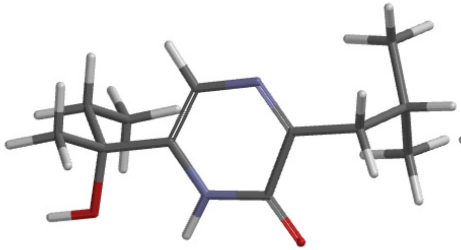

5 (3.8%)

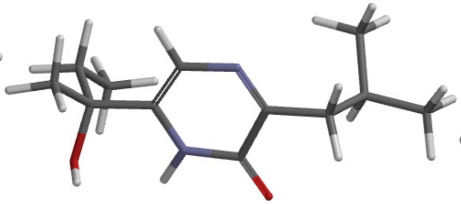

6 (3.7%)

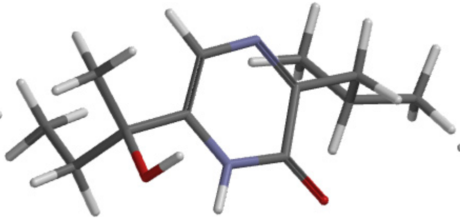

7 (3.6%)

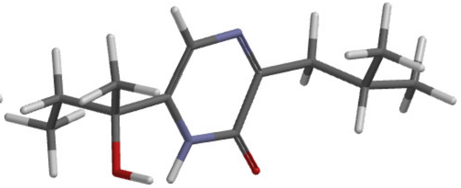

8 (3.5%)

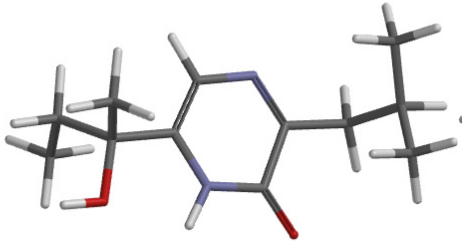

9 (3.5%)

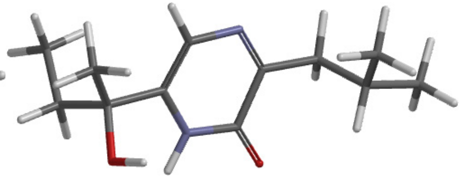

10 (3.4%)

Figure S54. The most stable conformers of 11*R*-**3** calculated at the B3LYP/6-31+G(d) level. Relative populations are in parentheses.

Table S1. X-ray Crystallography Data for **4**.

Empirical formula	$2(C_{12}H_{20}N_2O_2)$
Formula weight	448.60
Temperature/K	249.99(10)
Crystal system	monoclinic
Space group	$P2_1$
a/Å	11.77370(10)
b/Å	6.91360(10)
c/Å	16.5776(2)
α/°	90
β/°	91.3700(10)
γ/°	90
Volume/Å^3	1349.01(3)
Z	2
ρ_{calc}g/cm^3	1.104
μ/mm^{-1}	0.607
F(000)	488.0
Crystal size/mm^3	0.2 × 0.15 × 0.08
Radiation	CuKα (λ = 1.54184)
2Θ range for data collection/°	7.51 to 148.142
Index ranges	$-14 \leq h \leq 14$, $-8 \leq k \leq 8$, $-14 \leq l \leq 19$
Reflections collected	29460
Independent reflections	5242 [R_{int} = 0.0525, R_{sigma} = 0.0221]
Data/restraints/parameters	5242/1/299
Goodness-of-fit on F^2	1.039

Final R indexes [I>=2σ (I)]	R_1 = 0.0570, wR_2 = 0.1619
Final R indexes [all data]	R_1 = 0.0588, wR_2 = 0.1647
Largest diff. peak/hole / e $Å^{-3}$	0.52/-0.22
Flack parameter	-0.04(7)

Table S2.The hepatocellular carcinoma cell line HepG2 activity of manorubin B (**6**)

HepG2								
	DMSO (μL)	manorubin B (**6**) (μmol/L)						
	0.054	0.037	0.111	0.333	1	3	9	27
OD (570nm) [a]	1.44	1.437	1.377	1.294	0.996	0.454	0.388	0.379
Cell viability (100%) [b]		96.3	94.7	91.2	66.3	32.4	24.5	27.7
Inhibition rate (100%) [c]		3.7	5.3	8.8	33.7	67.6	75.5	72.3

[a] Average of three measurements, OD: Optical density, [b] Cell viability = Measurements value (OD)/Control value DMSO (OD) × 100%,

[c] Inhibition rate = 100% – Cell viability.

Table S3. The hepatocellular carcinoma cell line QGY7701 activity of manorubin B (**6**)

QGY7701								
	DMSO (μL)	manorubin B (**6**) (μmol/L)						
	0.054	0.037	0.111	0.333	1	3	9	27
OD (570nm)	1.46	1.320	1.310	1.147	0.423	0.255	0.235	0.188
Cell viability（100%）		90.2	89.5	78.4	28.9	17.4	16.1	12.8
Inhibition rate（100%）		9.8	10.5	21.6	71.1	82.6	83.9	87.2

Table S4. The nasopharyngeal carcinoma cell line SUNE1 activity of lunatinin (**7**)

SUNE1								
	DMSO (μL)	lunatinin (**7**) (μmol/L)						
	0.2	1.5625	3.125	6.25	12.5	25	50	100
OD (570nm)	0.791	0.668	0.672	0.578	0.452	0.391	0.364	0.368
Cell viability（100%）		84.5	84.9	73	57.2	49.5	46.1	46.5
Inhibition rate（100%）		15.5	15.1	27	42.8	50.5	53.9	53.5

Table S5. The hepatocellular carcinoma cell line HepG2 activity of lunatinin (**7**)

HepG2								
	DMSO (μL)	lunatinin (**7**) (μmol/L)						
	0.2	1.5625	3.125	6.25	12.5	25	50	100
OD (570nm)	0.422	0.373	0.347	0.233	0.142	0.111	0.085	0.092
Cell viability（100%）		88.3	82.3	55.3	33.6	26.2	20.1	21.7
Inhibition rate（100%）		11.7	17.7	44.7	66.4	73.8	79.9	78.3

Table S6. The hepatocellular carcinoma cell line QGY7701 activity of lunatinin (**7**)

QGY7701								
	DMSO (μL)	lunatinin (**7**) (μmol/L)						
	0.2	1.5625	3.125	6.25	12.5	25	50	100
OD (570nm)	0.976	0.915	0.758	0.479	0.216	0.162	0.102	0.097
Cell viability（100%）		93.8	77.7	49.1	22.1	16.6	10.4	9.9
Inhibition rate（100%）		6.2	22.3	5.9	77.9	83.4	89.6	90.0
